# Molecular Changes during Germination of Cocoa Beans, Part 1

**DOI:** 10.1021/acs.jafc.4c03523

**Published:** 2024-08-07

**Authors:** Konrad Brückel, Timo D. Stark, Corinna Dawid, Thomas Hofmann

**Affiliations:** † Food Chemistry and Molecular Sensory Science, TUM School of Life Sciences, Technical University of Munich, Lise-Meitner-Straße 34, 85354 Freising, Germany; ‡ Professorship for Functional Phytometabolomics, TUM School of Life Sciences, Technical University of Munich, Lise-Meitner-Straße 34, 85354 Freising, Germany

**Keywords:** MS, profiling, metabolites, S-plot, astringency, taste, HOJA, HMG glucosides

## Abstract

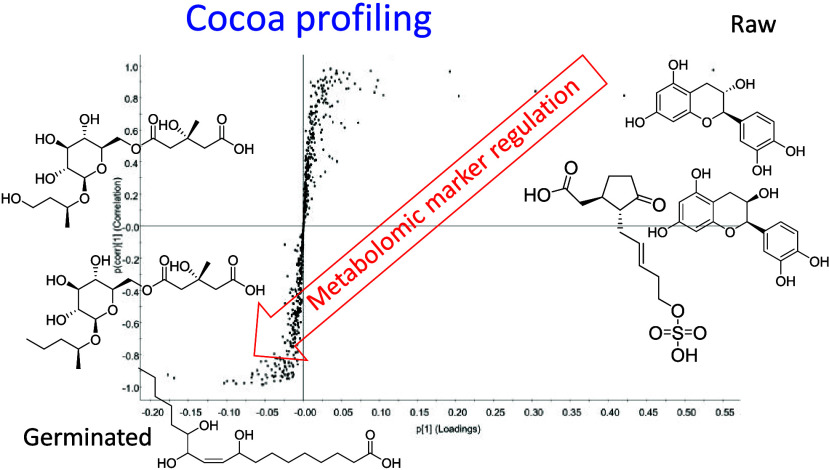

Some germination is known to occur during the process of fermentation in cocoa beans. The impact of this biological process on the course of cocoa fermentation is not known and was thus investigated. In order to determine the impact of germination at the molecular level as well as on flavor, an untargeted metabolomics approach using Ultra Performance Liquid Chromatography-Electrospray Ionization-Time of Flight-Mass Spectrometry (UPLC-ESI-ToF-MS) with simultaneous acquisition of low- and high-collision energy mass spectra (MS^e^) was performed. Extracts of raw and germinated cocoa beans of the same origin were measured and compared for characteristic differences by unsupervised principal component analysis. OPLS-DA revealed 12-hydroxyjasmonic acid (HOJA) sulfate, (+)-catechin and (−)-epicatechin as most down-regulated compounds as well as two hydroxymethylglutaryl (HMG) glucosides A and B among others as decisive up-regulated compounds in the germinated material. Additionally, further HMG glucosides and 12-hydroxyjasmonic acid could be identified in cocoa for the first time by coelution with isolated and synthesized reference compounds. HOJA sulfate, which has been postulated in cocoa, and HOJA were revealed to impart bitter and astringent taste qualities.

## Introduction

Cocoa beans and its derived products such as chocolate and cocoa powder are heavily consumed by humans with annual global harvest reaching about 5 million tons in 2019–2021.^1^ Cocoa beans are typically not consumed raw because of their characteristic high astringency and bitter flavor. Cocoa flavor is usually modulated to reach desired flavor attributes through postharvest processing including but not limited to fermentation, drying, and roasting. Previous studies had highlighted the contribution of cocoa fermentation in the formation of precursors of desirable taste-active compounds.^2−4^ Traditionally, postharvest treatment had comprised a fermentation process, initiated by a natural microflora, followed by drying and roasting before further processing. It has been revealed that during this step, the metabolome is not only affected by yeast and bacteria, but also by the beginning germination of the cocoa bean.^5,6^ However, the impact of germination on cocoa flavor has not been fully elucidated. Several studies investigated the effect of germination on the composition of the proteome and peptidome of cocoa.^7,8^ Misnawi et al. highlighted that several key enzymes in cocoa were still active in unfermented, dried cocoa powder, after an in vitro moisture treatment.^9^ Schlüter, Chetschik et al. revealed the effect of a novel moisture treatment of raw cocoa beans on volatile, odor-active components, in which fermentation was suppressed by pH adjustment and addition of ethanol, thus favoring germination.^10−12^ The resulting chocolate after this postharvest process led to a lower perception of the taste attributes of astringency and bitterness and reduced content of acetic acid upon comparison with chocolate produced from fermented and unfermented beans of the same origin.^13^ Several studies investigated those molecules affecting the unique taste of processed cocoa often associated with bitterness, while taste-active components in raw cocoa have been investigated only rarely. Theobromine and 2,5-diketopiperazines have been described as the most important bitter tastants in processed cocoa.^14−16^ Forsyth and Payne revealed the presence of flavan-3-ols like catechins and procyanidins,^2,17^ which were determined to be responsible for the astringency of raw and fermented beans as well as roasted nibs.^18−20^ Moreover, C-glycosylated flavan-3-ols had been highlighted as relevant for the velvety astringent taste sensation of alkalized cocoa.^21^ Since a degree of germination occurs during the fermentation process, an understanding of its contribution or impact to the changes occurring during fermentation is critical. Due to the similarities of fermentation and germination, as described by Stoll,^22^ knowledge of the germination metabolome will advance the scientific understanding of flavor development during cocoa beans germination and will enable scientific research dedicated to cocoa flavor development and fermentation. In this study, we propose to examine the impact of germination on taste active metabolites by comparing germinated, fermented, and raw cocoa beans. A systematic approach including metabolic profiling via multivariate statistics, fractionation, sensory screening, and chemical identification through synthesis was implemented to identify components that can be associated with astringency and bitterness modulation during the germination and fermentation of cocoa beans.

## Materials and Methods (Including Safety Information)

### Materials

#### Chemicals

The following chemicals were obtained commercially: 9,10,13-(*S*,*S*,*S*)-trihydroxyoctadec-11*E*-enoic acid (= 9,10,13-(11*E*) -THOA) and 9,12,13-(*S*,*S*,*S*)-trihydroxyoctadec-10*E*-enoic acid (= 9,12,13-(10*E*)-THOA) were purchased as ethanolic solutions from Larodan Inc. (Malmö, Sweden). 3-Hydroxy-3-methylglutaric acid glucoside (= HMG glucoside) A was received as a synthesis product from Mars, Incorporated (Hackettstown, NJ). 3-Hydroxy-3-methylglutaric acid (HMG) glucosides (= HMG glucosides) C, D, E, F, G, H, I, J, K, L, M, N and O were purchased from AnalytiCon Discovery GmbH (Potsdam, Germany) as isolates from different plant species. Hexyl sulfate was purchased as sodium salt from Merck. 1-Pentanol (analytic standard) was purchased from Dr. Ehrenstorfer GmbH (Augsburg, Germany), 1,3-butanediol (99%) was purchased from Alfa Aesar (Ward Hill, MA), (meso)-2,3-butanediol (98%) and 1,4-butanediol (99%) were both purchased from Sigma-Aldrich (Schnelldorf, Germany). Chemicals for the synthesis of 12-hydroxyjasmonic acid sulfate (methyl jasmonate, dimethyl sulfide, 3-bromopropan-1-ol, 3,4-dihydro-2*H*-pyran, triphenylphosphine, pyridinium *p*-toluene sulfonate) were purchased from Sigma-Aldrich (Schnelldorf, Germany).

Chromatography solvents, ACN and methanol, for mass spectrometry were purchased from CLN (Niederhummel, Germany) in LC-MS purity. Water as solvent was used after Millipore filtration with an AQUA-Lab – B30 – Integrity system (AQUA-Lab, Ransbach-Baumbach, Germany), aqueous solvents for chromatography were refreshed after 1 week. Formic acid as modifier for chromatography was purchased from Merck (Darmstadt, Germany) in purity >98%. All samples were stored at 5 °C in absence of light. Ground samples and sample solutions were stored at −18 °C until use/measurement.

#### Samples

Cocoa bean and liquor (i.e., ground roasted cocoa beans) samples, as listed in Table S1 in the Supporting Information (S.I.), were received from food industry.

### Profiling

#### Sample Preparation

An exact amount of 1 g of sample (bean homogenate and liquor samples, respectively) was placed into a Precellys sample tube (CK28_15 mL, Bertin Corp, Rockville).

#### Extraction

Methanol/water (70:30, v/v; 7.0 mL each) was added, the tube was closed, cooled at −18 °C for at least 15 min, the Precellys system was precooled to a temperature of max. Ten °C before the tube was put into the Precellys homogenizer (Bertin Corp, Rockville) and the extraction program was started (3 × 20 s rotation at 6200 rpm; intervals between of 20 s; 6 sample tubes parallel in one run). After the extraction step, the tubes were centrifuged at 2415*g* for 3 min and the supernatant was collected. Each sample tube was extracted three times in the way described above, the supernatants of each tube were pooled in a falcon (50 mL), respectively. These solutions were washed with *n*-pentane (distilled; 20 mL; 3 × 30 min at 350 rpm in a lab shaker), the supernatant was collected and the pentane phase was removed. Then, the defatted extracts were membrane filtered and the aliquots (5 μL) were analyzed by means of liquid chromatography/mass spectrometry (LC-MS/MS) using the gradients and mass transitions described below. Biological triplicates were prepared of each sample.

#### Liquid Chromatography/High Resolution Mass Spectrometry (UPLC-ESI-ToF-MS)

A Synapt G2-Si HDMS time-of-flight mass spectrometer (Waters, Milford, MA) was used to acquire electrospray ionization (ESI) mass spectra and product ion spectra. The electrospray was operated in the negative mode and the ToF-MS system was recording the data in the MS^e^ mode. Negative ions were detected at a capillary voltage at −2 kV using the following ion source parameters: source temperature (120 °C), cone voltage (50 V), source offset (40 V), source gas flow (0 mL/min), desolvation temperature (500 °C), cone gas flow (30 L/h), desolvation gas flow (900.0 L/h), nebulizer gas flow (2.5 bar). Survey scans were measured in a calibrated mass range from 50.0 to 1200.0 Da in high resolution mode with a scan time of 0.1 s. The parent survey was performed using collision energy (4.0 eV) and a low transfer MS collision energy (2.0 eV), while the daughter survey was performed using a ramped transfer MS collision energy (20.0–40.0 eV). The data were corrected by lock mass infusion (pentapeptide leucine enkephalin, scan time 0.3 s, interval 15 s). The column oven temperature was set to 40 °C. The samples were separated by means of an Acquity UHPLC Core system (Waters, Milford, MA), consisting of a binary solvent manager including a degasser, a sample manager, a column oven and a tunable ultraviolet detector, and equipped with a ACQUITY UPLC 2.1 mm × 150 mm, 130 Å, 1.7 μm, BEH C18 column (Waters, Milford, MA). Operated with a flow rate of 0.4 mL/min using 0.1% formic acid in water (v/v) as solvent A and 0.1% formic acid in ACN (v/v) as solvent B, chromatography was performed with the following gradient: 1% B held for 1 min, increased in 11 min to 99% B, held 2 min isocratically at 99% B, decreased in 0.2 min to 1% B and held for 0.8 min at 1% B. Data acquisition and instrumental control were performed with MassLynx v4.1 SCN 851 software (Waters, Milford, MA).

#### Measurement

The pooled samples were prepared in triplicates in the following way: aliquots of the first sample workup of all germinated samples were combined to yield the first germinated pool sample, proceeding with the second and the third sample workup in the same manner. Analogously pooled nongerminated (raw and fermented) samples were produced in triplicates consisting of aliquots. Finally, as a quality control for the profiling, a reference pool including aliquots of all measured samples was mixed. All biological triplicates were measured twice to achieve six replicates per sample in total. Furthermore, solvent blanks were measured. All samples were randomized before measurement and quality control samples were measured after eight profiling samples.

### Identification of (+)-Catechin, (−)-Epicatechin, HOJA, HOJA Sulfate, 9,10,13-(11*E*)-THOA and 9,12,13-(10*E*)-THOA and HMG Glucosides A, C, D, E, F, G, H, J, K, N and O

A set of cocoa bean and liquor samples, including raw, fermented and germinated samples of different origins, was selected for identification. The samples were worked up according to the procedure described in the profiling section. Sample solutions were prepared in different dilutions (1:10, 1:100 and 1:1000, v/v) in methanol/water (50:50, v/v, 1.00 mL). Standard stock solutions for catechin, epicatechin, 12-hydroxyjasmonic acid, 12-hydroxyjasmonic acid sulfate, 9,10,13-(11*E*)-THOA, 9,12,13-(10*E*)-THOA and for HMG glucosides A, C, D, E, F, G, H, J, K, N and O were prepared by separately dissolving aliquots of the pure standard compound in methanol/water (50:50, v/v, 1 mL) to achieve concentrations in a range of about 0.2–6.0 mg/mL. Standard compounds were tuned at a Xevo TQ-S mass spectrometer (Waters, Milford, MA). Identification was performed by comparison of mass transitions and retention times found in the sample solutions with those measured for the standard solution. Co-chromatography was used for confirmation.

### Isolation and Identification of HMG Glucoside B

#### Fractionation

Raw cocoa (provided by food industry) was extracted using solvent fractionation followed by GPC fractionation (modified from Stark et al.)^20^ and solid phase extraction (SPE) subfractionation to enrich the HMG glucoside B (see Supporting Information).

#### Enzymatic Assay Adopted from Literature^23,24^

Isolated hydroxymethylglutaryl glucoside A (10.5 μg, ∼24 nmol, 50 μL) was dissolved in water and diluted in sodium acetate buffer (130 μL, 3 g/L, the pH adjusted to 4.8 with glacial acetic acid). After addition of β*-*glucuronidase (from *Helix pomatia*, 150,000 units, 20 μL), the mixture was incubated while slowly stirring it in a lab shaker (37 °C, 5 days). The protein was precipitated by addition of methanol and followed by centrifugation (10 min at 6708*g*). The supernatant was membrane-filtered (0.45 μm) and used for analysis. A second assay was analogously prepared using cocoa isolate from subfraction GPCV/SPE4 (30% methanol).

#### HS-SPME-GC × GC-ToF MS

Different SPME-fibers (Pink Fiber: 65 μm film thickness PDMS/DVB; Blue Fiber: 85 μm film thickness CAR/PDMS, Supelco) were trialed in pre-experiments, in which a Carboxen/Poly(dimethylsiloxane) coating showed best performance for the used standard mix consisting of 1,3-butanediol and 2,3-butanediol (corresponding to the postulated HMG glucoside with *m*/*z* 395) as well as 2-pentanol (corresponding to the already isolated HMG glucoside with *m*/*z* 393). The samples (400 μL) were placed in headspace vials (20 mL) and the vials were capped and placed into the tray of a Combi PAL autosampler (CTC Analytics, Zwingen, Switzerland) held at 20 °C. Extraction for 20 min was performed using 65 μm PDMS/DVB fibers (Supelco, Sigma-Aldrich).

The instrument (Leco, Mönchengladbach, Germany) consisted of a 7890 gas chromatograph (Agilent, Waldbronn, Germany) equipped with a KAS4 injector (Gerstel, Mühlheim/Ruhr, Germany) and a DB-FFAP column (30 m × 0.25 mm i.d., 0.25 μm film; Agilent), a liquid nitrogen-cooled dual-stage quad-jet thermal modulator, a secondary oven mounted inside the primary GC oven and equipped with a DB-5 column (30 cm × 0.15 mm i.d., 0.30 μm film; Agilent), and a Pegasus III ToF MS (Leco, St. Joseph, MI) connected via a heated (250 °C) transfer line. Compounds were desorbed during 1 min at 250 °C. After analysis, the fibers were baked out at 270 °C for 10 min. Helium at 2 mL/min constant flow served as the carrier gas. The temperature of the primary oven was 35 °C for 5 min, ramped at 4°/min to 240 °C, and held at 240 °C for 10 min. The modulation time was 4 s. The temperature of the secondary oven was 70 °C for 2 min, ramped at 4 °C/min to 255 °C, and held at 255 °C for 10 min. The mass spectrometer was operated in the electron ionization (EI) mode at 70 eV, a scan range of *m*/*z* 35–350, and a scan rate of 100 spectra/s. Two-dimensional (2D) chromatograms were plotted using GC Image 2.1b5 (ZOEX Corporation, Houston, TX).

#### Identification of Released Alcohols

An alkane mixture (C_9_ – C_16_), aliquots of both prepared enzymatic assays and a mixture of the assumed alcohols (1,3-butanediol, 2,3-butanediol and 2-pentanol) were measured using the HS-SPME-GC × GC-ToF MS method described above. The retention indices of the measured alcohols were calculated and compared with literature. Unknown compounds were identified by comparing the EI fragments with the NIST database using MS Search v.2.2 (National Institute of Standards and Technology, Gaithersburg, MD).

### Sensory Analysis

#### Training of the Sensory Panel

Twelve individuals (7 males and 5 females, aged 23–34) with no history of known taste disorders, who gave their informed consent to participate in the sensory tests, were trained to evaluate the taste of aqueous solutions (1 mL each) of the following standard taste compounds by using a triangle test as described in the literature:^25^ caffeine (1 mmol/L) for bitter, lactic acid (20 mmol/L) for sour, and sucrose (12.5 mmol/L) for sweet taste. The sensation of the attributes of puckering astringency and velvety, mouth-drying astringency was trained by using gallotannic acid (0.05%) and quercetin-3-*O-*β-d-glucopyranoside (0.002 mmol/L), respectively, in half-tongue tests.^26^ Sensory training and analysis sessions were repeated twice on three different days in a sensory panel room at 22–25 °C.

#### Pretreatment of Fractions

Prior to sensory analysis, the solvents were removed under reduced pressure 30 to 40 mbar and freeze-dried twice. Stark et al. confirmed that this procedure sufficiently removes solvents and buffer compounds.^21^ Sensory analysis was performed using taste dilution analysis (TDA) for evaluation of bitter, sour and sweet taste quality, whereas astringency was evaluated by half-tongue test according to Stark et al.^20^

#### TDA of Fractions

Aliquots of the GPC fractions, respectively, were dissolved in “natural” ratios in 20 mL of bottled water (pH 6.0) and, sequentially diluted 1:1 with bottled water. The serial dilutions of each of these fractions were then presented to the sensory panel in order of ascending concentrations, and each dilution was evaluated by a triangle test. The dilution at which a taste difference between the diluted extract and the blank (reference) could just be detected, was defined as the taste dilution (TD) factor.^25^ Fractions I and II did not contain sufficient material for sensory evaluation. Fractions III to XIII were evaluated at least twice in different sessions and the TD factors were averaged. Fractions XIV to XXIV were evaluated only once.

#### Half-Tongue Test

TD factors, along with human astringency recognition thresholds, were determined by means of the recently developed half-tongue test, using bottled water as the solvent.^27^ Serial 1:1 dilutions of the samples were presented in order of increasing concentrations to a trained panel of 12 persons in three different sessions using the sip-and-spit method, while rinsing with 1% ethanolic solution and waiting between different concentration steps. When the panellist selected the correct solution, i.e., the solution containing the analyte, the next higher concentration step was presented besides one blank as a proof for the correctness of the data. The geometric mean of the first recognized and the last unrecognized concentration was calculated and taken as the individual recognition threshold. The values between individuals and between three separate sessions differed by not more than plus or minus one dilution step; that is a threshold value of 3.0 μmol/L for 12-hydroxyjasmonic acid sulfate in bottled water (pH 6.0) represents a range from 1.5 to 6.0 μmol/L.

#### Determination of Taste Threshold Concentrations

Taste recognition thresholds, defined as the concentrations at which the typical taste qualities of the compounds were just detectable, were determined in bottled water by means of a triangle test.^25^ The values between individuals and between three separate sessions differed by no more than one dilution step; that is, a threshold value of 11.2 μmol/L for the bitter taste of 12-hydroxyjasmonic acid sulfate represents a range from 5.6 to 22.4 μmol/L. Bitterness was evaluated with a standard duo/trio test in Evian water at pH 6.0 using six steps of a 1:1 (v/v) dilution in three different days by 10 to 12 trained panelists. Astringency was separately evaluated in a half-tongue test. Thresholds of 12-hydroxyjasmonic acid were additionally determined at pH 4.0 in order to determine if the degree of dissociation of the carboxy function would have an influence on the taste threshold.

### Synthesis

#### 12-Hydroxyjasmonic Acid

Starting from methyl jasmonate (MeJA, 3.03 g, 13.4 mmol) synthesis of the immediate methyl(Z)-2-(3-oxo-2-(5-((tetrahydro-2*H*-pyran-2-yl)oxy) pent-2-en-1-yl)cyclopentyl) acetate (THP-protected 12-hydroxyjasmonate methyl ester) was performed according to Jimenez-Aleman et al.^28^ The product was purified by flash chromatography on silica (AcOEt/*n*-hexane, 1:2), concentrated under reduced pressure (40 mbar, 40 °C) and freeze-dried, and kept under argon atmosphere before further use, purity (>93.4%), yield (0.72 g, 2.21 mmol). The identity was verified by ^1^H/^13^C NMR, measured in CDCl_3_, and compared with the literature.

THP-protected 12-hydroxyjasmonate methyl ester (0.60 g, 1.84 mmol) was dissolved in MeOH (12 mL). Aqueous 0.3 M potassium hydroxide solution (12 mL, 2.88 mmol) was added, and the solution was stirred for 1 h at room temperature. Hydrolysis was quenched by adjusting the pH to 6 with 0.1 M HCl. The solvents and water were removed under reduced pressure via lyophilization. The yielded intermediate (0.56 g, 1.80 mmol) was dissolved in ethanol (15 mL) and, after addition of pyridinium p-toluene sulfonate (240 mg, 0.96 mmol), was heated to 55 °C and stirred for 2 h. The assay was quenched with water (150 mL) and extracted with ethyl acetate (150 mL). The organic phase was washed with brine, concentrated and purified via RP chromatography (phenyl hexyl column, water/ACN with 0.1% formic acid, with the collection of peaks according to ELSD detection). Starting with 100% aqueous phase, conditions were held for 3 min, increasing to 60% organic phase in 15 min, a further increase to 100% in another 5 min until holding for 5 min and returning to starting conditions for equilibration. The purity of the final product (0.35 g, 1.22 mmol, >81.7%) and its identity as 12-hydroxyjasmonic acid were confirmed by ^1^H/^13^C NMR, measured in MeOH-*d*_4_, by UHPLC-ESI-ToF-MS and by comparison with the literature.^24,29^

#### 12-Hydroxyjasmonic Acid Sulfate

According to a modified procedure, which had been established in pre-experiments, about 10 mg of 12-hydroxyjasmonic acid (81% purity) was dissolved in ACN (3.0 mL) and given into a dry Pyrex bulb with a stir bar (10 mL), about 180 mg of sulfur trioxide pyridine complex as well as 0.5 mL of pyridine were added and flushed with argon. The Pyrex bulb was kept at 105 °C for 1 h in a thermostat-controlled heated metal block. Afterward, the sulfonation assay was diluted with water (16 mL) and the pH was adjusted to pH 8 with aqueous ammonium hydroxide solution (3%). After about 15 min, the solvent was removed under reduced pressure (40 °C, 40 mbar) and concentrated to about 4 mL. The pH was adjusted to 8.0 with aqueous ammonium hydroxide solution (3%) following purification via SPE and preparative/analytical HPLC (see Supporting Information). The yielded product (3.2 mg, 10.3 μmol) was confirmed as 12-hydroxyjasmonic acid sulfate with a sufficient purity (>79.4%) for sensory purpose by ^1^H/^13^C NMR, measured in MeOH-*d*_4_, by UHPLC-ESI-ToF-MS and by comparison with the literature.^29^

### Identification in Cocoa Samples via Liquid Chromatography and Tandem Mass Spectrometry

#### Identification of Marker Candidates

ESI mass spectra and product ion spectra were acquired with a Waters Xevo TQ-S mass spectrometer. The MS/MS system was operated in the MRM mode, detecting negative ions at the following ion source parameters: capillary voltage at 2.00 kV, source offset at 50.0 V, source temperature at 150 °C, desolvation temperature at 600 °C, cone gas flow at 150 L/h, desolvation gas flow at 800 L/h, collision gas flow at 0.15 mL/min and nebulizer gas flow at 7.0 bar. Dwell time was adjusted to 9 ms for each measured transition. The column oven temperature was adjusted to 50 °C. For analysis of the metabolites, the MS/MS parameters were tuned to achieve fragmentation of the [M – H]^−^ molecular ions into specific product ions, with the optimized parameters illustrated in Table S4 (S.I.). For tuning, ACN/water solutions of each analyte and internal standard were introduced by means of flow injection using a syringe pump. The analytical separation using aliquots of 2 μL of the sample solution was performed on an Acquity UHPLC I-Class System (Waters, Milford, MA) comprising a binary solvent manager, sample manager, and a column oven fitted with an ACQUITY UPLC 2.1 mm × 150 mm, 130 Å, 1.7 μm, BEH C18 column (Waters, Manchester, U.K.), coupled to a Waters Xevo TQ-S mass spectrometer (Waters, Milford, MA). The system was run with the MassLynx 4.1 software (Waters), and the data processing and analysis were executed with TargetLynx (Waters).

Operated with a constant flow rate of 400 μL/min, the mobile phase was mixed from solvent A (0.1% formic acid in water) and solvent B (0.1% formic acid in ACN) using the following gradient: starting with 5%, solvent B was increased to 30% in 10 min and furthermore increased to 99% within 2 min to be kept constant at 99% for two more minutes before returning to starting conditions in 1 min and equilibrating for 1 min.

### Screening and Identification of Further HMG Glucosides

A QTRAP 6500+ mass spectrometer associated with an ExionLC (Sciex, Darmstadt, Germany) was used to acquire electrospray ionization (ESI) mass spectra and product ion spectra of HMG gluc A, B, C, D, E, F, G, H, I, J, K, L, M, N and O in order to verify the findings at the Waters Xevo TQS system, where due to a lack of sensitivity the confirmation had been unclear. The MS/MS system was operated in the multiple reaction monitoring (MRM) mode detecting negative and positive ions in the scheduled MRM mode. Negative ions were detected at an ion spray voltage at −4500 V (ESI-) and the following ion source parameters: curtain gas (35 psi), temperature (550 °C), gas 1 (55 psi), gas 2 (65 psi), collision-activated dissociation (−3 V), and entrance potential (−10 V). The samples were separated by an ExionLC UHPLC with a Kinetex 2.1 mm × 100 mm, 100 Å, 1.7 μm, C18 column (Phenomenex, Aschaffenburg, Germany). The column oven temperature was adjusted to 40 °C. The gradients and solvents used were identical to those used for the Waters system. Data acquisition and instrumental control were performed with Analyst 1.6.3 software (Sciex, Darmstadt, Germany). Data evaluation/integration was done by MultiQuant software (Sciex), calculations of regression and analyte concentrations were performed with Microsoft Excel.

### Confirmation of Standard Compounds by HRMS

#### QToF-MS

Standard solutions were additionally measured at a 6600 Sciex QToF-MS device to confirm their identity. Parameters can be found in the Supporting Information.

### Nuclear Magnetic Resonance (NMR) Spectroscopy

The identity of the standard compounds was confirmed by ^1^H-/^13^C NMR and COSY, HMBC and HSQC experiments, which were performed on a Bruker AMX 400-III spectrometer (Bruker, Rheinstetten, Germany). The evaluation of the experiments was carried out using Topspin 4.0.7 NMR software (Bruker, Rheinstetten, Germany). DMSO-*d*_6_ and MeOH-*d*_4_ were used as solvents, and tetramethylsilane (TMS) was used as the internal standard.

### Identified Compounds

#### 12-Hydroxyjasmonic Acid

##### HRMS (ESI^–^), *m*/*z* 225.1128 (100%, [M – H]^−^), 59.0138 (31%)

^1^H NMR (400 MHz, CD_3_OD): δ 1.56 (l H, m, H-5α), 1.95–2.54 (10 H, m, H-1, H-2, H-4, H-5β, H-6α, H-8), 2.69 (1 H, m, H-6β), 3.57 (2 H, t, *J* = 6.8), 5.50 (2 H, m, H-9 and H-10).^13^C NMR (400 MHz, CD_3_OD): δ 27.3 (C-8), 29.0 (C-5), 32.7 (C-11), 39.5 (C-4), 40.1 (C-1), 40.6 (C-6), 56.0 (C-2), 63.5 (C-12), 129.9 (C-9 and C-10),, 177.0 (CO_2_H), 222.6 (C-3).

##### 12-Hydroxyjasmonic Acid Sulfate, No. 6 (Figure 2)

HRMS (ESI^–^), *m*/*z* 305.0695 (29%, [M – H]^−^), 96.9599 (100%, [HSO_4_]^−^), 225.1128 (6%, [M-H_2_SO_3_]^−^), 59.0138 (6%).^1^H NMR (400 MHz, CD_3_OD): δ 1.56 (l H, m, H-5α), 1.94–2.50 (8 H, m, H-1, H-2, H-4, H-5β, H-6α, H-8), 2.48 (2 H, dt, *J*_1_ = 6.7, *J*_2_ = 6.7, H-11), 2.67 (l H, dd, *J*_1_ = 14.3, *J*_2_ = 3.8 Hz, H-6β), 3.99 (2 H, t, *J* = 6.8 Hz, H-12), 5.49 (2 H, m, *J*_1_ = 9.1, H-9 and H-10).^13^C NMR (400 MHz, CD_3_OD): δ 27.3 (C-8),29.0 (C-5), 29.5 (C-11), 39.5 (C-4), 40.1 (C-l), 40.6 (C-6), 55.9 (C-2), 69.3 (C-12), 129.0 (C-10), 130.5 (C-9), 177.0 (CO_2_H), 222.6 (C-3).

##### 9,10,13-(11*E*)-THOA and 9,12,13-(10*E*)-THOA No. 5 (Figure 2)

*HRMS* (ESI-), *m*/*z* 329.2307 (100%, [M – H]^−^), 229.1416 (45%), 211.1310 (86%), 183.1358 (16%), 171.0998 (80%), 139.11 (24%), 127.1097 (12%), 99.0789 (10%)

##### Postulated Pentyl Glucoside Sulfate, No. 1 (Figure 2)

HRMS (ESI^–^), *m*/*z* 329.0912 (precursor), 329.0898 (67%, [M – H]^−^), 241.0027 (5%, [C_6_H_10_O_8_S]^−^), 167.0376 (5%), 150.9685 (5%), 138.9695 (7%, [C_2_H_3_O_5_S]^−^), 122.9733 (7%, [C_2_H_3_O_4_S]^−^), 101.0257 (12%, [C_4_H_5_O_3_]^−^), 96.9595 (100%, [HSO_4_]^−^), 95.9495 (5%), 85.0251 (5%), 79.9554 (7%, [SO_3_]^−^), 71.0112 (12%), 59.0112 (7%), 55.0177 (7%).

##### HMG Gluc A, No. 2 (Figure 2)

HRMS (ESI^–^), *m*/*z* 393.1753 (14%, [M – H]^−^), 250.1349 (19%), 249.1374 (77%, [M-C_6_H_8_O_4_]^−^), 161.0451 (53%, [C_6_H_9_O_5_]^−^), 125.0239 (72%), 113.024 (35%), 101.0257 (100%, [C_4_H_5_O_3_]^−^), 99.0455 (65%), 85.0286 (26%), 71.0133 (23%), 59.016 (75%), 57.0384 (64%).

##### HMG Gluc B, No. 3 (Figure 2)

HRMS (ESI^–^), *m*/*z* 395.1524 ([M – H]^−^), 293.1297 (30%, [M-C_4_H_6_O_3_]^−^), 251.1118 (100%, [M-C_6_H_8_O_4_]^−^), 161.0447 (30%, [C_6_H_9_O_5_]^−^), 159.0252 (15%), 125.0234 (46%), 113.0210 (15%), 101.0222 (46%, [C_4_H_5_O_3_]^−^), 99.0426 (87%), 71.0143 (15%), 59.0121 (85%), 57.0330 (87%), 55.0168 (15%).

##### Postulated New HMG Gluc P, No. 4 (Figure 2)

HRMS (ESI^–^), *m*/*z* 407.1545 ([M – H]^−^), 263.1121 (35%, [M-C_6_H_8_O_4_]^−^), 161.0435 (34%, [C_6_H_9_O_5_]^−^), 159.028 (9%), 125.0222 (30%), 119.0317 (17%), 113.0235 (22%), 101.0228 (42%, [C_4_H_5_O_3_]^−^), 99.0435 (44%), 99.0079 (9%), 89.0233 (17%), 85.0278 (13%), 83.0133 (9%), 81.0326 (9%), 73.0274 (17%), 71.0136 (27%), 59.0128 (77%), 57.0336 (100%), 55.0542 (13%).

##### HMG Gluc C

HRMS (ESI^–^), *m*/*z* 365.1447 ([M – H]^−^), 221.0989 (26%, [M-C_6_H_8_O_4_]^−^), 161.0431 (11%, [C_6_H_9_O_5_]^−^), 125.0223 (13%), 117.9337 (7%), 113.0222 (8%), 101.0229 (28%, [C_4_H_5_O_3_]^−^), 99.0434 (29%), 85.0279 (7%), 83.0115 (7%), 73.0274 (9%), 71.0123 (12%), 59.0128 (59%), 57.0333 (100%).

##### HMG Gluc D

HRMS (ESI^–^), *m*/*z* 431.1181 ([M – H]^−^), 125.0212 (100%, [M – H]^−^), 101.0212 (7%, [C_4_H_5_O_3_]^−^), 99.0420 (18%), 97.0266 (9%), 57.0323 (52%).

##### HMG Gluc E

HRMS (ESI^–^), *m*/*z* 413.1452 ([M – H]^−^), 269.1001 (39%, [M-C_6_H_8_O_4_]^−^), 161.0422 (23%, [C_6_H_9_O_5_]^−^), 125.0215 (29%), 113.0212 (1%), 101.0218 (28%, [C_4_H_5_O_3_]^−^), 99.0425 (56%), 71.0113 (10%), 59.0119 (59%), 57.0325 (100%).

##### HMG Gluc F

HRMS (ESI^–^), *m*/*z* 435.2231 ([M – H]^−^), 292.1795 (6%), 291.1765 (70%, [M-C_6_H_8_O_4_]^−^), 161.0414 (17%, [C_6_H_9_O_5_]^−^), 125.0209 (23%), 113.0209 (9%), 101.0214 (33%, [C_4_H_5_O_3_]^−^), 99.0421 (59%), 71.0110 (11%), 59.0116 (57%), 57.0325 (100%).

##### HMG Gluc G

HRMS (ESI^–^), *m*/*z* 427.1615 ([M – H]^−^), 125.0211 (36%), 119.0312 (6%), 113.0212 (13%), 101.0213 (12%, [C_4_H_5_O_3_]^−^), 99.0423 (55%), 89.0216 (14%), 71.0112 (7%), 59.0117 (54%), 57.0324 (100%), 55.0530 (5%).

##### HMG Gluc H

HRMS (ESI^–^), *m*/*z* 351.1281 ([M – H]^−^), 207.0837 (41%, [M-C_6_H_8_O_4_]^−^), 161.0417 (12%, [C_6_H_9_O_5_]^−^), 125.0212 (19%), 113.0212 (9%), 101.0215 (24%, [C_4_H_5_O_3_]^−^), 99.042 (23%), 85.0268 (5%), 73.0269 (5%), 71.0111 (9%), 59.0115 (53%), 57.0323 (100%),

##### HMG Gluc I

HRMS (ESI^–^), *m*/*z* 515.1765 ([M – H]^−^), 209.0789 (41%), 178.055 (8%), 177.0537 (100%), 162.0288 (8%), 125.0214 (13%), 99.0424 (15%), 57.0325 (16%),

##### HMG Gluc J

HRMS (ESI^–^), *m*/*z* 471.1835 (9%, [M – H]^−^), 369.1523 (9%, [M-C_4_H_6_O_3_]^−^), 328.145 (16%), 327.1433 (100%, [M-C_6_H_8_O_4_]^−^), 310.1352 (8%), 309.1318 (77%), 165.0893 (54%), 125.0215 (18%), 101.0217 (16%, [C_4_H_5_O_3_]^−^), 99.0424 (22%), 59.0118 (9%), 57.0326 (40%).

##### HMG Gluc K

HRMS (ESI^–^), *m*/*z* 515.2443 (41%, [M – H]^−^), 413.2125 (25%, [M-C_4_H_6_O_3_]^−^), 372.2064 (15%), 371.2022 (96%, [M-C_6_H_8_O_4_]^−^), 353.1918 (14%), 161.0414 (27%, [C_6_H_9_O_5_]^−^), 125.0211 (67%), 113.0211 (11%), 101.0215 (36%, [C_4_H_5_O_3_]^−^), 99.0424 (100%), 71.0111 (11%), 59.0119 (97%), 57.0324 (74%).

##### HMG Gluc L

HRMS (ESI^–^), *m*/*z* 638.1433 (13%), 637.1383 (66%, [M – H]^−^), 575.1368 (15%), 536.1084 (15%), 535.1047 (71%, [M-C_4_H_6_O_3_]^−^), 494.0977 (16%), 493.0942 (100%, [M-C_6_H_8_O_4_]^−^), 331.0408 (54%), 330.0339 (68%), 316.0179 (17%), 315.0104 (21%),

##### HMG Gluc M

HRMS (ESI^–^), *m*/*z* 651.1515 (30%, [M – H]^−^), 589.1513 (10%), 550.1229 (9%), 549.1185 (46%, [M-C_4_H_6_O_3_]^−^), 508.1111 (7%), 507.1085 (42%, [M-C_6_H_8_O_4_]^−^), 346.0594 (13%), 345.0565 (100%), 344.0482 (26%), 331.0368 (8%), 330.0327 (49%), 329.0253 (15%).

##### HMG Gluc N

HRMS (ESI^–^), *m*/*z* 529.2271 (51%, [M – H]^−^), 427.1938 (14%, [M-C_4_H_6_O_3_]^−^), 385.1829 (32%, [M-C_6_H_8_O_4_]^−^), 367.1731 (42%), 205.12 (25%), 153.089 (100%), 152.0812 (16%), 125.0217 (35%), 101.0219 (17%, [C_4_H_5_O_3_]^−^), 99.0427 (56%), 59.0119 (27%), 57.0326 (31%).

##### HMG Gluc O

HRMS (ESI^–^), *m*/*z* 576.1990 (10%), 575.1944 (53%, [M – H]^−^), 473.1610 (44%, [M-C_4_H_6_O_3_]^−^), 432.1554 (16%), 431.1522 (100%, [M-C_6_H_8_O_4_]^−^), 299.1093 (11%), 191.0523 (19%), 161.0418 (12%, [C_6_H_9_O_5_]^−^), 149.0421 (21%), 99.0059 (10%), 89.0217 (9%), 57.0322 (8%).

##### (+)-Catechin, No. 8 (Figure 2)

HRMS (ESI^–^), *m*/*z* 289.0694 (6%, [M – H]^−^), 203.0695 (21%), 159.0425 (22%), 151.0377 (27%), 137.0223 (28%), 125.0222 (38%), 123.043 (100%), 122.0342 (17%), 121.0275 (26%), 109.0277 (78%), 97.0281 (21%), 83.0122 (18%), 57.0334 (21%).

##### (−)-Epicatechin, No. 7 (Figure 2)

HRMS (ESI^–^), *m*/*z* 289.0702 (6%, [M – H]^−^), 188.0461 (21%), 159.0437 (24%), 151.0374 (24%), 137.0219 (27%), 135.0424 (22%), 125.023 (36%), 123.044 (100%), 122.036 (23%), 109.0284 (95%), 97.0278 (40%), 95.0492 (27%), 57.0339 (23%).

### Screening and Quantification of Known Taste Active Compounds by ^1^H NMR Spectroscopy According to Hammerl et al.^30^

#### Preparation of the Samples

About 5 mg of the freeze-dried GPC-fractions were accurately weighed into glass vials, dissolved (ultrasonication) in a mixture of 60 μL of the NMR buffer with 540 μL D_2_O, quantitatively transferred into 5 mm × 178 mm NMR tubes (USC tubes, Bruker, Faellanden, Switzerland) and stored at 5 °C until measurement.

#### Preparation of the NMR Buffer

KH_2_PO_4_ (10.2 g) in D_2_O (40 mL), adding KOH (1.5 g), TMSP-*d*_4_ (50 mg), and NaN_3_ (5 mg) followed by pH adjustment to 7.0 with a solution of KOH (4 mol/L) in D_2_O and filled up to 50 mL with D_2_O.

#### Screening

^1^H/^13^C experiments on reference compounds and GPC fractions were recorded at 300 K on a Bruker AVANCE NEO 500 MHz system (Bruker, Rheinstetten, Germany) equipped with a Cryo-Probe (CP 2.1 TCI 500 S2 H–C/N-D-05 Z XT) and Topspin 4.0.7 software. A database created by Hammerl et al.,^30^ containing 117 compounds, was used for screening and identification of known taste active compounds. The identifiable compounds were additionally quantified by quantitative ^1^H NMR spectroscopy at a 400 MHz system according to Frank et al.^31^ These measurements were performed at 298 K using a Bruker AV III system (Bruker, Rheinstetten, Germany) operating at a frequency of 400.13 MHz, equipped with a Z-gradient 5 mm multinuclear observe probe (BBFOplus). Water signals resulting from traces were decoupled.

## Results/Discussion

### Profiling

**Figure 1:**
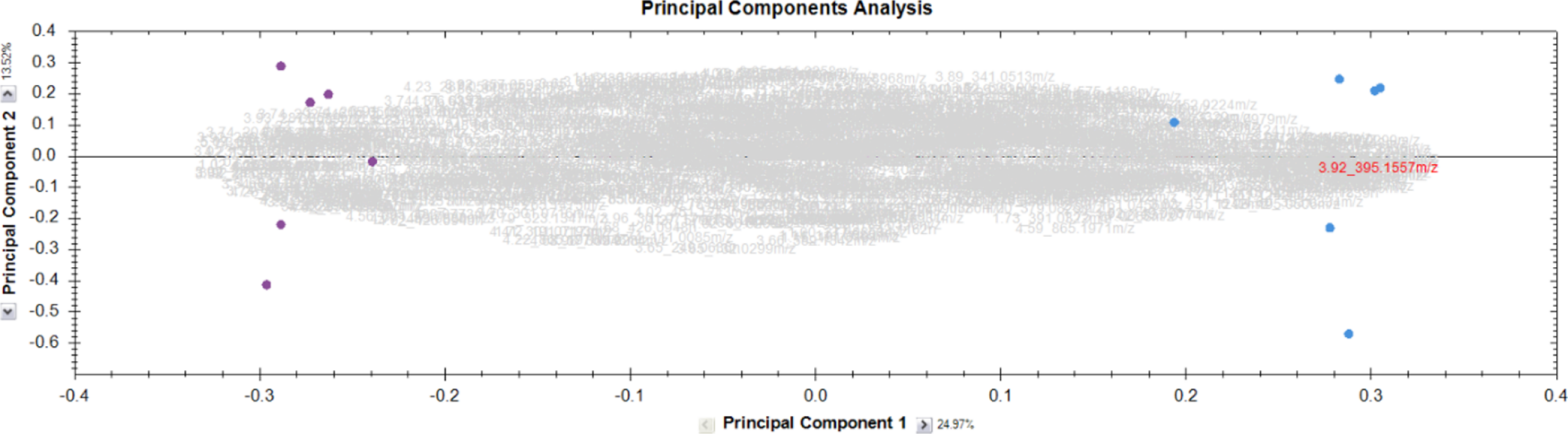
PCA biplot of UHPLC-ESI-ToF-MS full scan analysis comparing germinated (blue) and nongerminated (purple) pool samples (each dot represents one measurement of the pooled samples); *m*/*z* 395.1552 (rt = 3.92 min) is highlighted (in red) as characteristic compound of the germinated samples.

**Figure 2:**
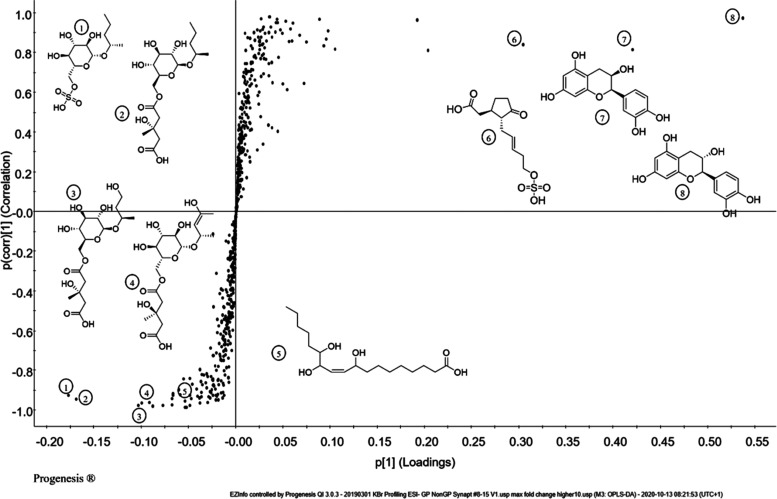
S-plot of pool germinated (−1) versus pool corresponding raw (1) samples with identified marker candidates.

In a first approach those metabolites were determined which are affected most by germination and, therefore, could be used as markers for this postharvest process. Thus, sets of samples comprising raw and germinated samples were compared by means of a nontargeted profiling via Ultra Performance Liquid Chromatography coupled with Electrospray Ionization Time-of-Flight Mass Spectrometry (UPLC-ESI-ToF-MS). PCA (principal components analysis) was performed for pooled samples (comparing germinated and nongerminated samples, depicted in Figure 1) as well as for each cocoa variety by Progenesis Studio, respectively, and S-plots were produced by EZ-info software. Figure 2 highlights an example of an S-plot for the comparison of pooled germinated and nongerminated samples. Accurate mass to charge ratios found were used to establish possible molecular formulas. Table 1 indicates an overview of the most promising marker candidates found by PCA and S-plot analysis of UHPLC-ESI-ToF-MS measurements of the samples mentioned above. Compounds with *m*/*z* 393.1768, 395.1552, 407.1555, 305.0697, 289.0719, 329.0912, and 329.2328 could be determined as presumable marker candidates derived from the S-plot analysis. The chemical identification and synthesis for each of these compounds is described below.

**Table 1:** Marker Candidates Derived from S-Plot Analysis of Germinated and Corresponding Nongerminated Pool Samples; Most Likely Molecular Formulas and Compounds Predicted by MassLynx Elemental Composition Tool, Fragmentation, and Literature Search

							suggested compound	
no. in S-plot	*m*/*z* in ESI-	*t*_R_ (min)	calculated mass	Δ*m* (mDa)	Δ*m* (ppm)	suggested formula (M – H)	IUPAC name	abbreviation
1	329.0912	4.22	329.0906	0.6	1.8	C_11_H_21_O_9_S	((2*R*,3*S*,4*S*,5*R*,6*R*)-3,4,5-trihydroxy-6-(((*S*)-pentan-2-yl)oxy)tetrahydro-2*H*-pyran-2-yl)methyl sulfate	pentyl gluc sulfate
2	393.1768	5.17	393.1761	0.7	1.8	C_17_H_29_O_10_	(*R*)-3-hydroxy-3-methyl-5-oxo-5-(((2*R*,3*S*,4*S*,5*R*,6*R*)-3,4,5-trihydroxy-6-(((*S*)-pentan-2-yl)oxy)tetrahydro-2*H*-pyran-2-yl)methoxy)pentanoic acid	HMG gluc A
3	395.1552	3.92:3.75	395.1553	0.1	0.3	C_16_H_28_O_11_	(*R*)-3-hydroxy-3-methyl-5-oxo-5-(((2*R*,3*S*,4*S*,5*R*,6*R*)-3,4,5-trihydroxy-6-(((*S*)-4-hydroxybutan-2-yl)oxy)tetrahydro-2*H*-pyran-2-yl)methoxy)pentanoic acid	HMG gluc B
4	407.1555	4.10	407.1553	0.2	0.5	C_17_H_27_O_11_	(*R*)-3-hydroxy-3-methyl-5-oxo-5-(((2*R*,3*S*,4*S*,5*R*,6*R*)-3,4,5-trihydroxy-6-(((*S*,*E*)-4-hydroxypent-3-en-2-yl)oxy)tetrahydro-2*H*-pyran-2-yl)methoxy)pentanoic acid	HMG gluc P
5	329.2328	7.07	329.2328	0	0	C_18_H_33_O_5_	(*Z*)-9,12,13-trihydroxyoctadec-10-enoic acid	THOA
6	305.0697	4.30	305.0695	0.2	0.7	C_12_H_17_O_7_S	2-((1*R*,2*R*)-3-oxo-2-((*E*)-5-(sulfooxy)pent-2-en-1-yl)cyclopentyl)acetic acid	HOJA sulfate
7	289.0719	4.23	289.0712	0.7	2.4	C_15_H_13_O_6_	2-hydroxy-4-((2*S*,3*R*)-3,5,7-trihydroxychroman-2-yl)phenolate	EC
8	289.0717	3.92	289.0712	0.5	1.7	C_15_H_13_O_6_	2-hydroxy-4-((2*R*,3*R*)-3,5,7-trihydroxychroman-2-yl)phenolate	Cat

### Identification of Supposed Marker Candidates

#### Identification of Postulated HOJA/HOJA Sulfate

The potential marker compound with the molecular formula of C_12_H_17_O_7_S seemed most likely to correspond to 12-hydroxy jasmonate sulfate, which was described in cocoa by Patras and Milev.^32,33^ As mentioned in patents by Hurst,^34,35^ treatment of cocoa beans by fermentation presumably reduces its content. According to Miersch,^36^ 12-hydroxy jasmonate sulfate and 12-hydroxy jasmonate, among other jasmonate derivatives, play an important role in plant growth and germination. Thus, a synthesis approach, derived from the procedure used by Jimenez-Aleman et al.,^28^ was developed with methyl jasmonate as a starting material (Figure S3, S.I.), in order to gain 12-hydroxyjasmonic acid. In a second synthesis, the hydroxy moiety was sulfonated with sulfur trioxide using a modified in-house method. After purification by preparative HPLC, a yield of 4.0 mg (79.4% purity by qNMR) was attained (Figure S4, S.I.), which was used for sensory evaluation of the taste thresholds as well as for spiking experiments. Furthermore, coelution experiments with an aqueous extract of raw cocoa and a solution of the synthesized HOJA sulfate could unequivocally confirm the identity of this marker candidate. In a similar manner, HOJA could be identified and confirmed in the cocoa samples chosen for screening.

#### Identification of Postulated Trihydroxy Octadecenoic Acids

According to previous profiling experiments (Table 1), *m*/*z* ratios of 329.2328 had been considered as marker candidates. The suggested molecular formula (C_18_H_33_O_5_) revealed possible isomers of THOAs. Consequently, commercially available isomers were tuned at the LC-MS/MS system and the presence of 9,10,13-(11*E*)-THOA and 9,12,13-(10*E*)-THOA (both in *S*,*S*,*S* configuration) could be confirmed in the chosen samples by coelution experiments with reference compounds.

#### Identification of (+)-Catechin and (−)-Epicatechin

According to the literature,^2,17^ (+)-catechin and (−)-epicatechin belong to the more abundant phenolic compounds in cocoa. The highest p(1) scores in the S-plot (Figure 2) were found for *m*/*z* ratios 289.072 at 3.92 and 4.23 min (Table 1). The suggested molecular formula C_15_H_13_O_6_ could be assigned to (+)-catechin and (−)-epicatechin. Co-chromatography with reference compounds revealed (+)-catechin at 3.92 min and (−)-epicatechin at 4.23 min.

#### Identification of HMG Glucosides A and B

**Figure 3:**
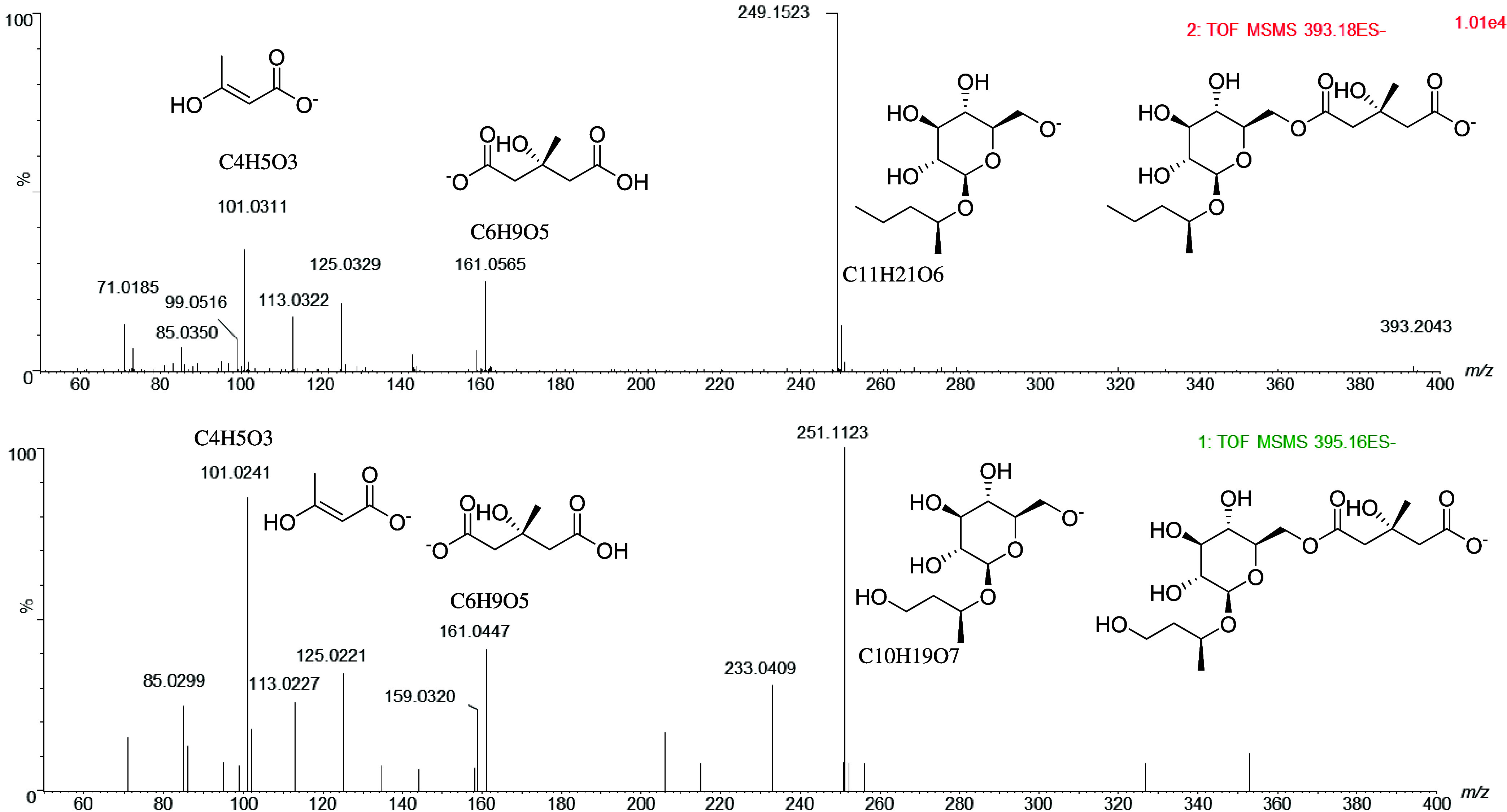
MS Daughter scans received from UHPLC-qToF measurement with aligned fragments of postulated HMG glucosides A (top) and B (bottom).

For the molecular formula C_17_H_30_O_10_ literature research suggested a hydroxymethylglutaryl glucoside (HMG gluc).^37,38^ Enhanced generation of fragments was necessary for structure elucidation in order to distinguish different possible compounds. To verify the supposed HMG gluc with *m*/*z* 393, a standard compound (HMG gluc A) was used to confirm the retention time and fragmentation of the supposed marker compound. The identity and purity of the standard compound were additionally determined by NMR measurements as well as UHPLC-ESI-ToF-MS measurement. The identity of HMG gluc A (*m*/*z* 393, no. 2 in Table 1 and Figure 2) could be confirmed in the liquor samples due to the same retention time and MRM transitions found in the reference run and the sample runs. MS^2^-experiments using qToF-MS (Synapt, Waters) using *m*/*z* 393 and 395 (no. 3 in Table 1 and Figure 2) as the precursors resulted in similar fragmentation patterns for both of these compounds, thus indicating related structures (Figure 3). Hydroxymethylglutaryl glucoside A generated major fragments 249 and 161, but fragment 251 could not be explained by an analog compound with *m*/*z* 395. Thus, further investigation of the literature was necessary. However, the fragment with *m*/*z* 251 retained for the precursor with *m*/*z* 395 could be explained by an exchange of a methyl group against a hydroxy group in the terpenoid moiety, which would correspond with the most probable molecular formula of C_10_H_19_O_7_ determined for the accurate mass of 251.1123. In order to enrich this assumed new HMG glucoside B, solvent fractionation according to Stark et al. was applied (depicted in Figure S5, S.I.).^20^ In a second step, the remaining water-soluble compounds were fractionated by GPC, with the chromatogram presented in Figure S 6 (S.I.). Analysis of the fractions by UHPLC-ESI-ToF-MS revealed highest yields of the corresponding *m*/*z* 395 in fractions IV and V (illustrated in Figure S7, S.I.). GPC fraction V was used for further isolation by means of solid-phase extraction. This fractionation resulted in the highest yields in SPE fractions 4 and 5. The chromatograms (Figure S8, S.I.) depict the extracted *m*/*z* of 395.155 in all SPE fractions. To obtain a preliminary confirmation of the structure and especially of the moiety linked to the anomeric carbon of the glucose of the HMG glucoside, an enzymatic assay was developed to release this moiety and determine its structure. For the postulated candidate with *m*/*z* of 395 (ESI neg) this moiety seemed most likely to consist of butanediols. Two possible isomers 1,3-butanediol and 2,3-butanediol were used to develop a useful assay to determine the alcoholic moiety after release from the marker candidate. Thus, it was decided to establish a GC method for analysis of the released moieties without derivatization. Due to the buffer salts present in the enzymatic assay, a headspace SPME method was used, and comprehensive two-dimensional gas chromatography (HS-SPME-GC × GC-ToF MS) was used to attain cleaner mass spectra. Pre-experiments with a mix of 1,3-butanediol and 2,3-butanediol (corresponding to the postulated HMG glucoside with *m*/*z* 395) as well as 2-pentanol (corresponding to the already isolated HMG glucoside with *m*/*z* 393) were used to determine optimum fiber material and equilibration conditions as well as the GC gradient and temperature offset of the second column oven. Using this optimized method, a proof of concept was performed first, wherein isolate of HMG glucoside with *m*/*z* 393 was hydrolyzed in the enzymatic assay and analyzed by HS-SPME-GC × GC-ToF MS. In the 2D plot, the corresponding 2-pentanol could be identified, proving that this approach offered sufficient sensitivity and selectivity. Consequently, the SPE extracts 4 and 5 gained from GPC fraction V were subjected to this combined assay. In Figure S9 (S.I.), 2D peaks for all three of the measured alcohols were assigned according to their mass spectra in EI mode. The horizontal axis illustrates the first-dimension separation on the polar DB FFAP column, the vertical axis shows the second-dimension separation on the short nonpolar VF5 column. The proof of concept is depicted in Figure S10 (S.I.), where the enzymatic release of 2-pentanol could be detected from the HMG glucoside A standard with *m*/*z* 393. The chromatogram after enzymatic release from fraction GPCV/SPE4, in which isomers of the postulated HMG glucoside B could be isolated (Figures S7 and S8, S.I.), is depicted in Figure S11 (S.I.). In this run, 2D peaks (areas of higher concentration/peaks in the plotting of 2D chromatography) related to 2-pentanol and 2,3-butanediol could be identified. 1,3-butanediol, however, was not detected in the assay containing the SPE subfraction 4. One can conclude that, the release of 2,3-butanediol from the purified isolate along with that of 2-pentanol from the HMG glucoside A standard, confirms the structure of the assumed HMG glucoside B. Given these results, 64 different stereoisomers can be postulated with (α-l/β-d-) galactose and glucose, which are shown in Figures S12–15 (S.I.).

### Screening of Commercially Available HMG Glucosides

Generally, the data collected thus far indicated that HMG glucosides might be affected by the cocoa process and serve as potential marker compounds for germination. However, previous experiments with HMG glucosides A and B had shown that in contrast to the more abundant HMG gluc A, there was a low concentration of HMG gluc B and that further HMG glucosides may be present in even lower concentrations and, therefore, have not been detected by the nontargeted approach.

Due to their possibly low concentration in cocoa, similar to the HMG gluc B, isolation from cocoa was not deemed feasible. However, during further research, commercial sources of a variety of HMG glucosides could be found. After confirming the purity of these structures by qNMR, these compounds were tuned at the UHPLC-MS/MS system and identification in a test set of cocoa samples was performed by coelution experiments.

**Figure 4:**
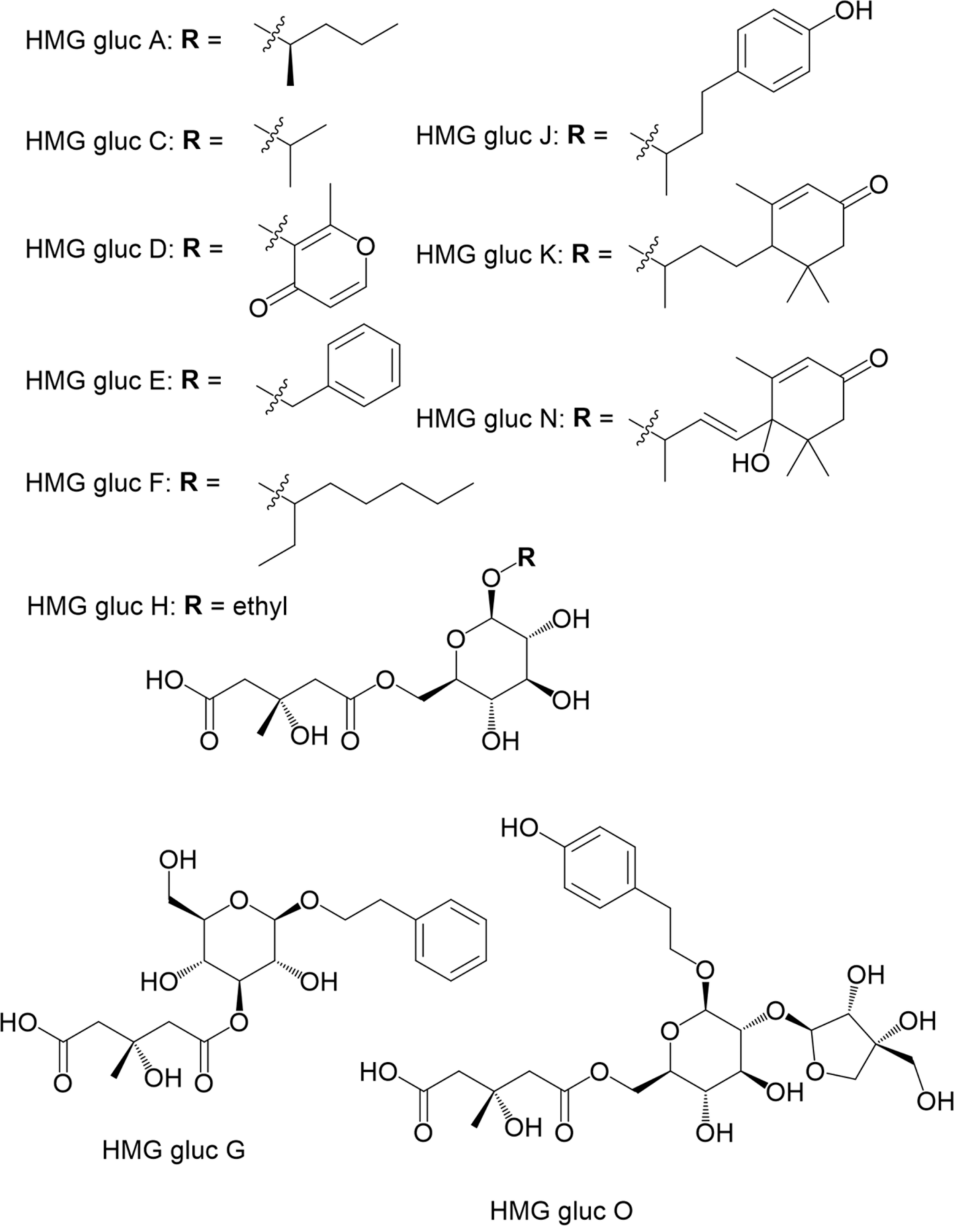
HMG glucosides identified in cocoa by commercial standard compounds.

Figure 4 depicts those structures which could be identified in cocoa samples and their occurrence in literature. Esters of HMG gluc C conjugated with two different dihydroxy prenyl furanocoumarin moieties, respectively (citrusosides B and C), were found in extracts of *Citrus hystrix* fruits by Youkwan et al.^39^ The isopropyl glycoside moiety, which is connected to the HMG moiety in HMG gluc C, was first identified in fennel by Kitajima et al.^40^ HMG gluc D (licoagroside B) was first described in *Glycyrrhiza glabra* hairy root cultures by Li et al.,^41^ in chickpea by Mekky et al.,^42^ in *Ononis arvensisL.* by Gampe et al.^43^ and in further plants. HMG gluc E was described in *Hylocereus undatus* by Wu et al.^44^ and in *Roylea cinerea* (Lamiaceae) by Sharma et al.^45^ HMG gluc G was also identified in *Hylocereus undatus*(44) and in *Mimusops elengi*(46) where an inhibitory effect on the enzymatic activity of hyaluronidase was detected. HMG gluc J was found in the bark of *Betula platyphylla* by Kim et al.^47^ An isomer of HMG gluc K with the HMG moiety acylated in glucose-3 position, was described in roots of leek by Schliemann et al.^48^ HMG gluc M was found in Citrus by Sawabe et al.^49^ Although HMG gluc N has not been described itself, it consists of roseoside, which was initially identified by Bhakuni et al. in *Vinca rosea*,^50^ acylated with HMG. HMG gluc O was found in pea nut germs by Kitagawa et al.^51^ However, it is not clear, which physiological role these compounds could have.

### Summary Identification

According to the previous identification experiments, the most promising marker candidates indicated by PCA and S-plot could be identified, which are depicted in Figure 2. Marker candidates, outstanding for the germinated samples, are situated on the bottom left, while marker candidates, which represented raw samples, appear on the top right corner of the S-plot. Compounds, characteristic for germinated samples, number 2 (HMG gluc A) and number 3 (HMG gluc B), could be identified with standard compounds, whereas number 4 was postulated as another HMG gluc derivative due to its similar fragmentation pattern as well as number 1, which according to its accurate mass and fragmentation pattern was postulated as a derivative of HMG gluc A with a sulfate group replacing the HMG moiety. Number 5 could be identified as trihydroxy octadecenoic acid. Compounds characteristic of raw samples were (+)-catechin (No. 8), (−)-epicatechin (No. 7) and 12-hydroxyjasmonic acid sulfate (No. 6).

### Taste Contribution of Marker Compounds

#### Solvent-Guided Fractionation in Accordance with the Literature

In a first step, raw cocoa beans were peeled, the shell was discarded and the kernel was examined. After freezing in liquid nitrogen, the sample material was grinded to powder and extracted by a variety of solvents of different polarity. These different solvent fractions were freeze-dried, redissolved in water and again freeze-dried, and yields were determined by weighing (Figure S5, S.I.). Fractions I and V combined accounted for more than 80% (m/m) of the overall yield, while fractions II, III and IV were comparatively low. However, in comparison with the results obtained by Stark et al.^20^ for fermented and roasted cocoa nibs, these remaining fractions are twice as high in their combined yield, which highlights the differences between raw and processed/roasted cocoa beans. While in the processed nibs the share of pentane extractables was increased to 45.8% (found by Stark in fermented and roasted nibs), in the raw cocoa this yield was reduced to 39.3%. Of the remaining extractables, Stark et al. had found lower yields in the processed material than this work detected in the raw cocoa beans, with DCM extractables 1.3% (Stark: 0.3%), ethyl acetate extractables 4.0% (Stark: 0.7%) and water extractables 11.7% (Stark: 6.8%).

#### Profile Analysis of Solvent Fractions

Aqueous solutions of these fractions were again rated by sensory profile analysis to gain an insight into the polarity of the most potent taste-active compounds. As depicted in Figure S19 (S.I.), the highest scores were found for astringency, bitterness and sourness in the aqueous extract, lower scores were found for these in the ethyl acetate extract and in the dichloromethane extract. The pentane extract did not impart any noteworthy taste quality (besides some slight cocoa-buttery fatty impression) and neither did the insoluble residue, thus indicating that the extraction of most taste-active compounds had been completed and that most relevant taste-active compounds feature high polarity. Due to the rather low taste activity found in the pentane and the ethyl acetate extract, further isolation, and fractionation experiments where focused only on water extractables.

#### Fractionation of the Aqueous Extract by Gel Permeation Chromatography

GPC fractionation (Figure S6, S.I.) yielded 24 fractions, which were used for TDA experiments after suspending in water, removal of solvents in high vacuum (<5 mPa) and lyophilization in duplicate.

#### Taste Dilution Analysis of GPC Fractions

The fractions obtained after GPC and post-treatment were evaluated by TDA with respect to the taste qualities bitter, sour and sweet (Table 2). GPC fractions I and II did not contain yields after lyophilization and thus were not evaluated by TDA. The taste quality astringency was evaluated in separate half-tongue tests described by Scharbert et al.^26^ As depicted in Figure S20 (S.I.) the attribute “sweet” was found highest in fractions III to V, whereas sourness was described highest in fractions V and VI. Fraction V moreover imparted a relatively high TD factor for bitterness, whereas for astringency highest intensities were found in GPC fractions XI, XVI, and XX.

**Table 2:** Yields, Taste Qualities, and Taste Dilution (TD) Factors of GPC Fractions Isolated from Aqueous Fraction of Raw Cocoa Bean Extract

fraction^a^	yield^b^ (mg)	taste quality^c^	TD factor^c^	bitter and astringent compounds identified^d^
I	<1			
II	<1			
III	6.4	bitter	64	kaempferol-3-glc
		sweet	64	
IV	75.7	astringent	2	
		bitter	32	
		sweet	16	
V	861.0	astringent	64	HMG glucoside A, HOJA sulfate, sucrose^e^, citric acid^e^
		bitter	128	
		sour	256	
		sweet	128	
VI	45.7	astringent	2	HOJA sulfate, HMG glucoside A, caffeoyl-Ser, sucrose^e^, formic acid^e^, citric acid^e^, succinic acid^e^
		bitter	512	
		sour	256	
VII	33.3	astringent	4	cinnamoyl-Asp, caffeoyl-Pro, feruloyl-Asp, *p*-coumaroyl-Glu, feruloyl-Glu
		bitter	32	
		sour	32	
		sweet	16	
VIII	129.7	astringent	8	theobromine, caffeine, theophylline
		bitter	64	
		sour	64	
		sweet	1	
IX	11.6	astringent	128	caffeoyl-Met, Caffeoyl-Ser
		bitter	32	
		sweet	1	
X	14.9	astringent	64	rutin
		bitter	64	
		sweet	8	
XI	15.5	astringent	256	procyanidin A2
		bitter	64	
		sweet	1	
XII	14.6	astringent	32	catechin-6,8-diglcp, catechin-6-C-glcp, isorhoifoline
		bitter	64	
		sour	16	
		sweet	1	
XIII	7.7	bitter	64	resveratrol, *p*-coumaroyl-Tyr/-Dopa, naringenin-7-glc
		sweet	1	
XIV	12.7	astringent	8	vitexin, orientin, apigenine-7-glc, kaempferol-7-neohesperidosid
		bitter	128	
		sweet	1	
XV	40.2	astringent	128	kaempferol-3-glc, quercitrin, luteolin-7-glc, catechin, epicatechin
		bitter	16	
		sweet	1	
XVI	55.8	astringent	256	caffeoyl-Trp, quercitrin, procyanidins B2/A2
		bitter	16	
		sweet	1	
XVII	10.2	bitter	128	procyanidins A2/B2/C1
		sweet	1	
XVIII	1.7	astringent	<1	procyanidins A2/B2/C1
		bitter	32	
		sweet	1	
XIX	87.2	astringent	128	caffeoyl-Pro, procyanidins A2/B2/C1
		bitter	32	
		sweet	1	
XX	97.7	astringent	256	procyanidins A2/B2/C1
		bitter	32	
		sour	32	
		sweet	1	
XXI	67.5	astringent	128	procyanidins A2/B2/C1
		bitter	32	
		sweet	1	
XXII	26.2	bitter	128	caffeoyl-Ser, procyanidins A2/B2/C1
		sweet	1	
XXIII	57.6	bitter	16	procyanidins A2/B2/C1
		sour	64	
		sweet	1	
XXIV	222.5	astringent	64	procyanidins A2/B2/C1
		bitter	32	
		sweet	1	
XXV	118.8	nd	<1	

a Number of GPC fraction referring to Figure S6 (S.I.).

b Yields were determined by weight after lyophilization.

c The taste quality and the TD factor were determined by using a half tongue test for astringency and a triangle test for the other attributes.

d Compounds were identified by UHPLC-MS/MS with reference compounds.

e Compounds were identified by ^1^H NMR using library screening.

#### Contribution of Known Taste-Active Compounds

Quantification of known bitter and astringent tastants in the GPC fractions used for TDA revealed that O-glucosides and procyanidins are eluted in GPC fractions X and higher, whereas acid amides were located in fractions VII, VIII and higher (Table 2). Consequently, the high TD factors for astringency, bitterness and sourness found by TDA in fractions V and VI can hardly be explained by the already known tastants but probably by unknown tastants in these two fractions. In order to identify small molecules (with a molecular weight below 50 Da), which were not found by mass spectrometry without targeted analysis enabled by chemical derivatization,^52,53^ and polar or ionic compounds, which were not separated sufficiently by RP-chromatography, NMR spectroscopy screening with the GPC fractions V and VI was performed according to Hammerl et al.^30^^1^H NMR spectra are depicted in Figure S22 (S.I.), the compounds identified are listed in Table S6 (S.I.). Taste-active compounds formic acid, citric acid, acetic acid, lactic acid, succinic acid and glutaric acid connected with a sour/salty taste impression could be identified, as well as sucrose and l-alanine (sweet taste impression) and l-aspartic acid (umami taste impression). l-Aspartic and glutaric acid could not be determined by quantitative ^1^H NMR spectroscopy in any of the fractions due to low signal/noise ratios. For the other compounds, contents and DoT factors were calculated. Upon comparison with the results of the TDA (Table 2), the high DoT factors of sucrose and citric acid in GPC fraction V explain the high TD factors found for attributes sweet and sour, whereas in the GPC fraction VI a combination of succinic acid, formic acid and citric acid might account for the high TD factor of the attribute sour. However, the high TD factors of bitter and astringent could not be explained by these already known compounds, as the e.g., the known astringent caffeoyl-serine and other astringent compounds present in these GPC fractions were concentrated below their individual threshold concentrations.

#### Influence of Jasmonate Derivatives on Bitter and Astringent Taste

Previous measurements had revealed the presence of the postulated 12-hydroxyjasmonic acid sulfate in raw and fermented cocoa beans. To determine if this compound might contribute to the strong bitter taste found in GPC fraction VI, this compound was tuned at the Waters TQ-S UHPLC-MS/MS system and semiquantitatively measured. It could be found in highest abundance in the GPC fraction VI (Figure S23, S.I.). After synthesis and purification, this compound could be confirmed as 12-hydroxyjasmonic acid sulfate. To evaluate its taste impact, a taste dilution analysis of a solution of 12-hydroxyjamonic acid sulfate and the assumed precursor compound 12-hydroxyjamonic acid were staged to determine taste threshold values (Table 3).

**Table 3:** Taste Thresholds and DoT Factors of HOJA and HOJA Sulfate

compound	descriptor	threshold in Evian water pH 6.0 (μMol/L)	concentration in sample #84 (μMol/L)	DoT factor in sample #84
12-HOJA	bitter	46.0	13.0	0.28
	astringent	1.0		13.0
12-HOJA sulfate	bitter	11.2	1240	111
	astringent	3.0		414
epicatechin	astringent	930 (Scharbert et al.)	57,800	62.2

Table 3 depicts taste thresholds of 12-hydrojasmonic acid sulfate, 12-hydroxyjasmonic acid and (−)-epicatechin as well as the concentration and the DoT factor in a raw cocoa bean sample (#84, S.I.). Due to the high DoT factors for bitterness and astringency found for 12-hydroxyjasmonic acid sulfate, the overall taste impression of the raw cocoa might be highly influenced by this compound. Additional TDA experiments with solutions at pH 4.0 did not indicate pH dependence of the taste thresholds of both jasmonate derivatives within this range, which however is in contrast to previous findings on the pH dependence of astringent sensation in organic acids.^54^ In a further sensorial evaluation, the type of astringency was described as rather velvety (4 of 6 panelists) than puckering (2 of 6 panelists) upon comparison with (−)-epicatechin as reference for puckering astringency and quercetin-*O*-glucoside as reference for velvety astringency. Previous experiments by Stark et al. had discovered that DoT factors alone may not account for the actual taste impact of a compound, due to saturation effects and solubility limitations.^55^ To compare the effect of HOJA sulfate on astringency with that of (−)-epicatechin, a solution of (−)-epicatechin in Evian pH 6.0 (360 μmol/L) was tasted against the same epicatechin solution spiked with 12-hydroxyjasmonic acid sulfate (3.9 μmol/L) in a half-tongue setup. Out of 20 tests, an increased intensity of the astringency could only be found in 13 cases for the spiked solution. On a level of significance of 0.05 this means that the impact of 12-hydroxyjasmonic acid sulfate on astringency at a DoT factor of about 1 is not significant in the presence of (−)-epicatechin in concentrations even lower than a DoT factor of 1. However, due to the much higher DoT factors of more than 400 found for HOJA sulfate in the raw cocoa sample (Table 3) and of about 60 found for (−)-epicatechin, HOJA sulfate might still be a significant contributor to astringency in the natural ratios.

### Outlook

Metabolomic experiments have revealed several compounds, which appear to correlate to germination of cocoa material, by comparing germinated samples with their raw equivalents via principal components analysis and S-plot analysis. Some of these compounds have been revealed to be flavor active and to contribute to the overall taste of cocoa. Among these, besides the known bitter tastants (+)-catechin and (−)-epicatechin, the newly described 12-hydroxyjasmonic acid sulfate could be confirmed as a characteristic compound, which appeared to be reduced by germination. On the other hand, derivatives of 3-hydroxy-3-methylglutaric acid glucoside as well as isomers of trihydroxyoctadecenoic acid seem to be increased by this process. Due to the findings that HMG glucosides appeared to be influenced by germination, an additional screening for the commercially available HMG glucosides known from the literature was performed, in which several new HMG glucosides could be identified in cocoa for the first time. Therefore, it is considered necessary to evaluate their significance as possible marker compounds unique to the cocoa process used, which would be based on a comparison of raw, germinated and fermented samples. Consequently, accurate quantification will be needed in subsequent experiments due to a higher sensitivity, selectivity and robustness compared to the ToF-MS instruments used for the profiling. Solvent guided fractionation of raw cocoa beans confirmed previous results obtained by Stark et al.^20^ for fermented and roasted nibs from West Africa, namely that the most bitter and astringent taste activity could be found in the aqueous extract after solvent-guided fractionation. Within the aqueous extract, HMG glucoside A and HOJA sulfate were detected in GPC fractions with the highest perception of bitterness as well as astringency, which could not be explained by the presence of known taste active compounds in these fractions. The low threshold concentrations and high DoT factors of HOJA sulfate and HOJA furthermore indicate a major role of these compounds in the overall taste of raw cocoa. The astringency-enhancing quality of HMG glucoside A, as described by Didzbalis,^38^ might contribute to the astringent taste impression as well.

## Supporting Information Available

Description of the GPC fractionation procedure, results of PCA and trend plots of marker compounds; tuning parameters, retention times and mass transitions of identified marker candidates; results of solvent guided fractionation, GPC and SPE fractionation as well as identification of known taste-active compounds; chromatograms of HS-SPME-GC × GC-ToF MS assays after enzymatic release; chromatograms of UHPLC-MS/MS runs of marker compounds; UHPLC-ESI-ToF-MS, MS^e^, 1D- and 2D-NMR spectra of the identified marker compounds and HMG glucosides (PDF)

## Abbreviations Used

ACN: acetonitrile
Cat: (+)-catechin
*c*: mass concentration
DB: dried cocoa beans
DMSO: dimethyl sulfoxide
D_2_O: deuterium oxide
DoT: dose-over-threshold
EC: (−)-epicatechin
ESI: electrospray ionization
F: fermented
FA: formic acid
*g*: unit of gravity (relative centrifugal force)
G: germinated
GC × GC: comprehensive two-dimensional gas chromatography
GC: gas chromatography
GPC: gel permeation chromatography
HILIC: hydrophilic interaction chromatography
HMG: 3,3-hydroxymethyl glutaryl/glutarate
HOJA: 12-hydroxyjasmonic acid
HPLC: high-performance liquid chromatography
HS-SPME: headspace solid-phase microextraction sampling
IS: internal standard
KPI: key process indicator
rpm: rotations per minute
LA: Latin American
Liq: cocoa liquor
LOD: limit of detection
LOQ: limit of quantification
MeOH: methanol
MF: microfermented
MRM: multiple reaction monitoring (mode)
MS: mass spectrometry
MS^e^: pseudo MS^2^ scan using high collision energy
MS/MS: tandem mass spectrometry
MS^2^: mass spectrometric experiment measuring fragment ions generated from a specific precursor
NMR: nuclear magnetic resonance spectroscopy
OPLS-DA: orthogonal partial least-squares discriminant analysis
PCA: principal component analysis
qNMR: quantitative NMR
R: recovery
S.I.: supporting Information
SPE: solid-phase extraction
SPME: solid-phase microextraction
TDA: taste dilution analysis
THOA: trihydroxy octadecenoic acid
ToF-MS: time-of-flight mass spectrometry
UHPLC: ultrahigh-performance liquid chromatography
v/v: volume fraction
μm: micrometer
μL: microliter
