# Supplementary material for: Molecular Changes during Germination of Cocoa Beans, Part 1
Source: J Agric Food Chem. 2024 Aug 7;72(33):18606–18. doi: 10.1021/acs.jafc.4c03523 (PMC11342359; doi:10.1021/acs.jafc.4c03523)
Supplement: Supplementary file 1 — jf4c03523_si_001.pdf [file jf4c03523_si_001.pdf]

Supporting Information:

Molecular changes during germination of cocoa beans, Part 1

Konrad Brückel[†], Timo D. Stark[*†], Corinna Dawid[†,++] and Thomas Hofmann[†]

[†] Food Chemistry and Molecular Sensory Science, TUM School of Life Sciences, Technical University of Munich, Lise-Meitner-Straße 34, 85354 Freising, Germany

[++]Professorship for Functional Phytometabolomics, TUM School of Life Sciences, Technical University of Munich, Lise-Meitner-Straße 34, 85354 Freising, Germany

[*]Corresponding author

Materials and methods:

Cocoa samples:

Table S 1: Cocoa bean and liquor samples used

Batch	Sample No.(#)	Sample Description
1	10	Cocoa Liquor from unfermented non-germinated South East Asian Type 1a beans
	8	Cocoa Liquor from unfermented germinated South East Asian Type 1a beans
	11	Cocoa Liquor from unfermented non-germinated South East Asian Type 1b beans
	9	Cocoa Liquor from unfermented germinated South East Asian Type 1b beans
	13	Cocoa Liquor from fermented non-germinated South East Asian Type 1 beans
	12	Cocoa Liquor from fermented germinated South East Asian Type 1 beans
	15	Cocoa Liquor from low-fermented Latin American Type 1 beans
	14	Cocoa Liquor from high-fermented Latin American Type 1 beans
2	84	Unfermented dried Latin American Type 1 beans
	85	Alternatively fermented dried Latin American Type 1 beans
	86	Cocoa Liquor from unfermented Latin American Type 1 beans
	87	Cocoa Liquor from alternatively fermented Latin American Type 1 beans
3	94	Cocoa liquor from alternatively fermented (96 h) beans, high-roasted Latin American Type 4 beans
	95	Cocoa liquor from alternatively fermented (96 h) beans, unroasted Latin American Type 4 beans
	96	Cocoa liquor from alternatively fermented (0 h) beans, high-roasted Latin American Type 4 beans
	97	Cocoa liquor from alternatively fermented (0 h) beans, unroasted Latin American Type 4 beans

Batch 1 was used for the profiling, batches 2 and 3 were used for identification experiments.

Fractionation of aqueous extract of raw cocoa beans

Sequential solvent extraction

Raw beans (100 g; CCN51 provided by food industry) were peeled by hand, frozen in liquid nitrogen, crushed in a grinding mill, and then extracted with *n*-pentane (5 × 300 mL) at room temperature for 30 min. After centrifugation, the residual cocoa material was then extracted five times with a mixture (7:3, v/v; 300 mL each) of acetone and water for 45 min at room temperature with stirring. After filtration, the liquid layer was freed from acetone under reduced pressure at 30 °C, and the aqueous solution obtained was extracted with dichloromethane (5 × 150 mL) to remove theobromine and caffeine and then freeze dried to give the acetone/water extract.

Gel permeation chromatography (GPC)

A glass column with an inner diameter of 5 cm and a length of about 50 cm was packed with Sephadex LH-20 (27 – 163 μm, Sigma-Aldrich) as a slurry in methanol to yield a total column bed of 45 cm. A constant flow rate of 2.1 mL/min was applied by a peristaltic pump P-1, GE Healthcare Bio-Science AB (Uppsala, Sweden) and the effluent was guided through a Jasco UV detector equipped with a preparative cell (diameter 1,0 mm) measuring absorption at 270 nm wavelength. Fractions were collected by an Ultrorac Fraction Collector 2070 II (LKB, Bromma, Sweden) into 20 mL glass vials. 1.5 g of aqueous extract were dissolved in 80 mL of methanol/water 1:1 (v/v) in an ultrasonic bath and given onto the column bed dropwise. A gradient was applied according to Table S 2 (Supporting Information). 24 Fractions were collected (each peak and intervals between two peaks),

which were used for TDA experiments after suspending in water, removal of solvents in high vacuum (< 5 mPa) and two-fold lyophilization.

Table S 2: GPC elution gradient

**Elution solvent (premixed and degassed)**	**Elution time (h)**
Solvent 1; water (milli-Q) pH 3,5 (0.1 % formic acid) + Methanol (LC-Grade) **(1+1)**	4
Solvent 2; water (milli-Q) pH 3,5 (0.1 % formic acid) + Methanol (LC-Grade) **(3+7)**	14
Solvent 3; water (milli-Q) pH 3,5 (0.1 % formic acid) + Methanol (LC-Grade) **(1+9)**	8
Solvent 4; Methanol (LC-Grade) **100 %**	16.5
Solvent 5; Methanol (LC-Grade) + Acetone (GC Grade) **(9+1)**	5

Solid phase extraction (SPE)

533 mg of GPC fraction V were dissolved in 20 mL of eluent A (0.1 % formic acid in water), given onto a Strata C18-E SPE-cartridge (sorbent mass/volume, 5 g/20 mL; Phenomenex, Aschaffenburg, Germany) and eluted with mixtures (20 mL) of eluent A and increasing ratio (Table S 3) of eluent B (0.1 % formic acid in methanol (HPLC-grade)), fractions (20 mL) were collected and lyophilized after removal of solvents in high vacuum (< 5 mPa).

Table S 3: Solid phase extraction of GPC fraction V (533 mg), Gradient starting with 100 % of Eluent A (0.1 % Formic acid in water (Millipore® quality)) and increasing ratio of Eluent B (0.1 % Formic acid in Methanol (HPLC-grade)), yields determined after lyophilization.

**Fraction**	**Yield (mg)**	**Eluent B (0.1 % Formic acid in MeOH)**	**Volume eluent**
1	425.3	0%	20ml
2	10.5	10%	20ml
3	8.3	20%	20ml
4	3.8	30%	20ml
5	3	40%	20ml
6	1.3	50%	20ml
7	1.2	60%	20ml
8	0.5	70%	20ml
9	0.4	80%	20ml
10	0	90%	20ml
11	0.9	100%	20ml

Synthesis of HOJA sulfate:

SPE-purification. The synthesis assay was given onto two SPE cartridges (Chromabond C18ec, 6 mL, 1 g) and each eluted with water, followed by methanol/water 1/1 (v/v,) and methanol (HPLC grade). Each bed volume eluted was collected. Aliquots (200 µL) were transferred into HPLC vial inlets for UPLC-MS measurement. Fractions were concentrated under reduced pressure (40 °C, 40 mbar), in which solvents were removed, and lyophilized. Dry matters were redissolved in MeOH and transferred in pre-weighed glasses and yields were determined after removal of MeOH under a stream of nitrogen.

Preparative/analytical HPLC. The high-pressure liquid chromatography (HPLC) apparatus consisted of an AS-2055 Plus autosampler (Jasco, Groß-Umstadt, Germany), two PU-2087 Plus pumps (Jasco, Groß-Umstadt, Germany), a Degasys DG-1310 degasser (Uniflows, Tokyo, Japan), a MD-2010 Plus diode array detector (Jasco, Groß-Umstadt, Germany) connected to a Sedex LT-ELSD Model 85 detector (Sedere, Alfortville, France) by a split valve. For analytical measurements, the autosampler contained a 100 μL sample loop. For preparative scale, a six-way Rheodyne valve was used connected to a 2 mL sample loop. All modules were connected to a control unit and operated by Chrompass software (Jasco, Groß-Umstadt, Germany). The preparative column used was a Luna phenylhexyl column (250 × 21.2 mm) by Phenomenex (Aschaffenburg, Germany) operated with a constant flow of 21 mL/min.

The SPE fractions with highest yields of product (fractions 1 and 2) were purified via RP chromatography (phenyl hexyl column, water/acetonitrile gradient). Starting from 100 % water, which was kept for 5 min, the acetonitrile proportion of the eluent was increased to 15 % in 5 min, increased to 100 % in 5 min, keeping these conditions for 5 min, before returning to starting conditions and equilibrating for 5 min. Fractions were collected for each peak according to UV absorption at 200 and 220 nm, concentrated under reduced pressure and lyophilized.

Results:

Profiling:

PCA (pooled germinated samples in purple, pooled non-germinated samples in blue)

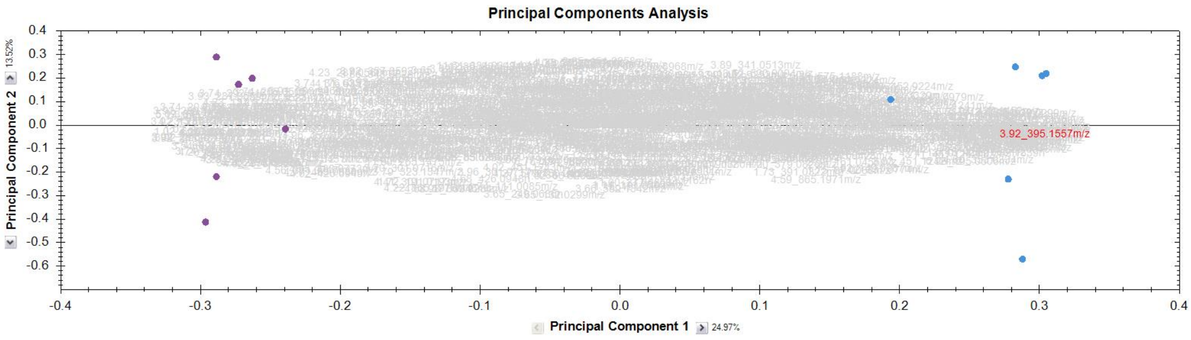

Figure S 1: PCA biplot comparing germinated (blue) and non-germinated (purple) pool samples (each dot is representing one measurement of the pooled samples); m/z 395.1552 (rt = 3.92 min) is highlighted (in red) as characteristic compound of the germinated samples.

Trend plot (*m/z* 395.1557, t_R 3.92 min)

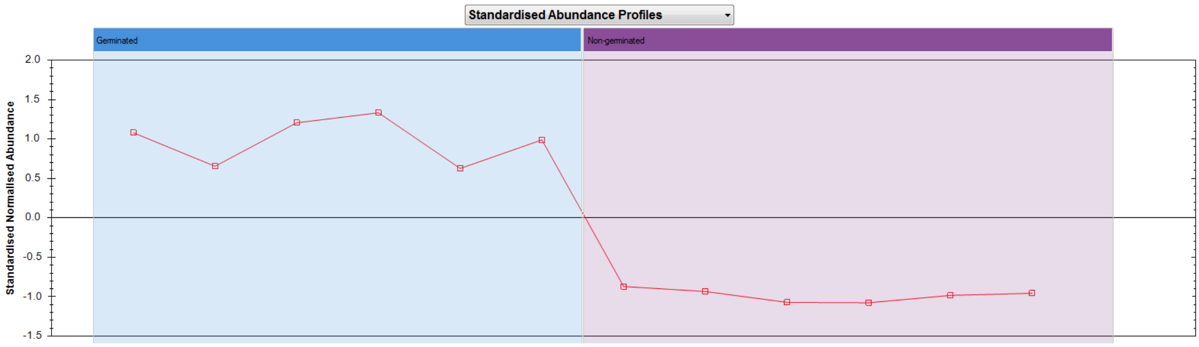

Figure S 2: Trend plot comparing abundance of m/z 395.1552 (rt = 3.92 min) in germinated (blue) and non-germinated (purple) pool samples

Identification of presumable marker candidates

Table S 4: Marker compounds identified after tuning at the Waters Xevo TQS system.

**Analyte**	**Retention time** (min)	**M-H** *(m/z)*	**Daughter** *(m/z)*	**CV** (V)	**CE daughter 1** (V)
HOJA	5.3	225.0	59.0	36	20
			97.1		24
			147.1		22
HOJA sulfate	4.2	304.9	96.9	36	22
			225.2		20
HMG gluc A	7.4 / (7.7)	393.1	99.1	18.0	14
			249.2		18
			125.1		12
			291.3		10
HMG gluc B	2.3 / 3.2 / 3.7	395.0	99.1	34	14
			251.2		14
			293.2		14
			125.1		16
HMG gluc C	4.1 / (4.5)	364.9	99.0	2	14
			125.1		14
			221.2		18
			263.2		8
9,10,13-(11E)-THOA	11.9	329.1	171.1	22	24
			229.2		21
9,12,13-(10E)-THOA	11.7	329.1	211.2	22	22
			229.2		21
Cat	3.3	289.1	245.0	48	14
			203.0		20
EC	4.2	289.1	245.0	48	14
			109.0		24

12-Hydroxyjasmonic acid (HOJA), 12-Hydroxyjasmonic acid sulfate (HOJA sulfate), Hydroxymethyl glutaroyl glucoside A (HMG gluc A), Hydroxymethyl glutaroyl glucoside B (HMG gluc B), Hydroxymethyl glutaroyl glucoside C (HMG gluc C), 9,10,13-(11E)-Trihydroxy octadecenoic acid (9,10,13,(11E)-THOA, 9,12,13-(10E)-Trihydroxy octadecenoic acid (9,12,13,(10E)-THOA, Catechin (Cat) and Epicatechin (EC) with the respective retention times using the standard gradient; retention times of additional isomers found are shown in brackets; mother ion (M-H), and daughter ions with their respective mass/charge ratios *m/z* and respective tuning parameters Cone Voltage (CV) and Collision Energy (CE).

Table S 5: Further HMG glucosides identified after tuning at AB Sciex 6500 system

**Analyte**	**Retention time** (min)	**M-H** *(m/z)*	**Daughter** *(m/z)*	**Dwell time** (ms)	**DP** (V)	**CE** (V)	**CXP** (V)
HMG gluc D	4.5	430.9	124.9	8	-35	-24	-15
			98.9	8	-35	-22	-11
HMG gluc E	6.8	413.0	268.9	8	-15	-24	-25
			99.0	8	-15	-22	-7
HMG gluc F	11.2	435.1	291.0	8	-45	-26	-35
			98.8	8	-45	-22	-11
HMG gluc G	7.3	427.0	99.0	8	-35	-20	-11
			142.9	8	-35	-16	-9
HMG gluc H	3.1	351.0	207.0	8	-5	-26	-31
			98.8	8	-5	-18	-13
HMG gluc J	7.1	471.0	326.9	8	-35	-28	-43
			369.1	8	-35	-22	-39
HMG gluc K	9.4	515.1	371.1	8	-70	-28	-35
			413.0	8	-70	-20	-47
HMG gluc L	7.5	637.0	329.7	8	-90	-50	-35
			492.9	8	-90	-36	-27
HMG gluc M	8.5	651.1	329.4	8	-95	-60	-47
			344.5	8	-95	-42	-31
HMG gluc N	6.6	529.1	385.0	8	-75	-26	-49
			367.0	8	-75	-30	-11
HMG gluc O	6.0	575.1	431.0	8	-40	-36	-49
			473.0	8	-40	-30	-17

Hydroxymethyl glutaroyl glucosides (=HMG gluc) A, C, D, E, F, G, H, J, K, L, M, N and O identified with the respective retention times found, mother ion (M-H), and daughter ions with their respective mass/charge ratios *m/z* and respective tuning parameters Dwell Time, Declustering Potential (DP), Collision Energy (CE) and Cell Exit Potential (CXP).

Synthesis of HOJA sulfate

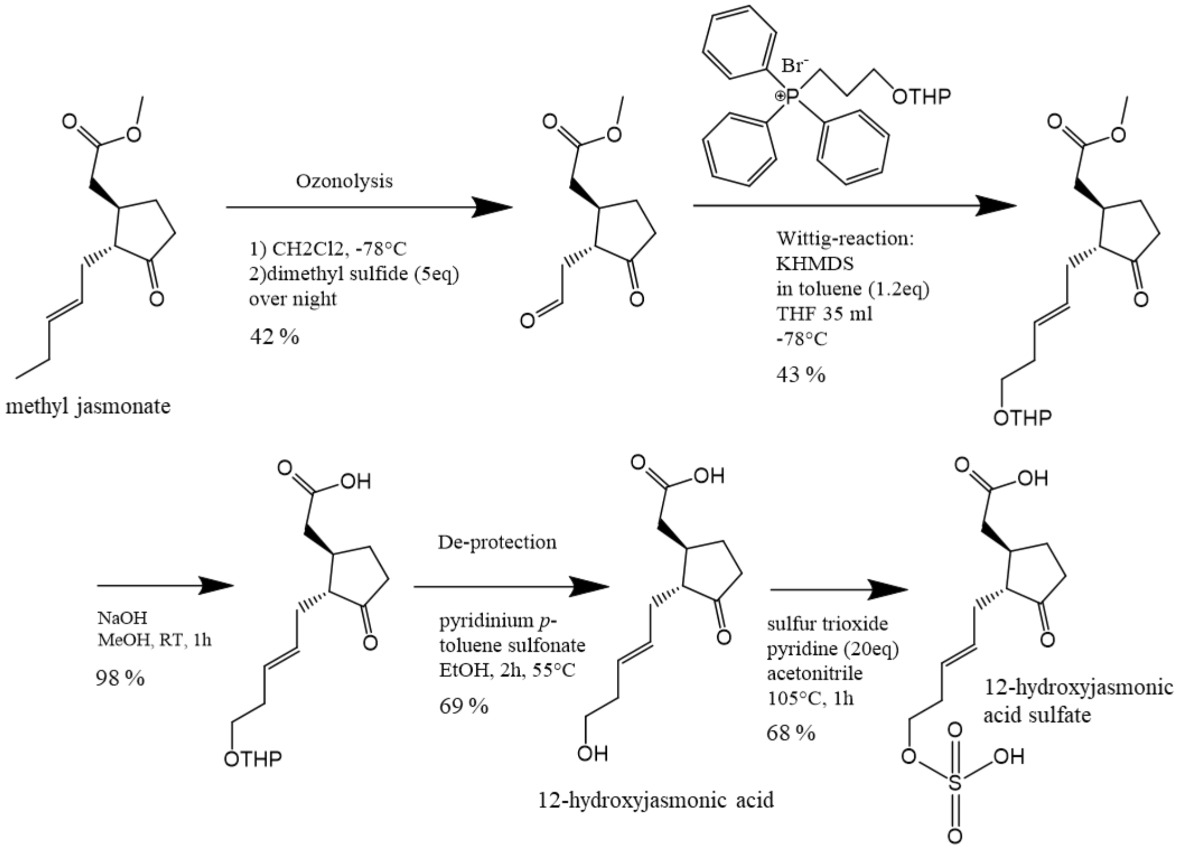

Figure S 3: Synthesis of 12-hydroxyjasmonic acid sulfate, Ozonolysis, synthesis of Wittig reagent, Wittig reaction and Deprotection according to Jimenez-Aleman et al., hydrolysis and sulfonation according to inhouse-method.

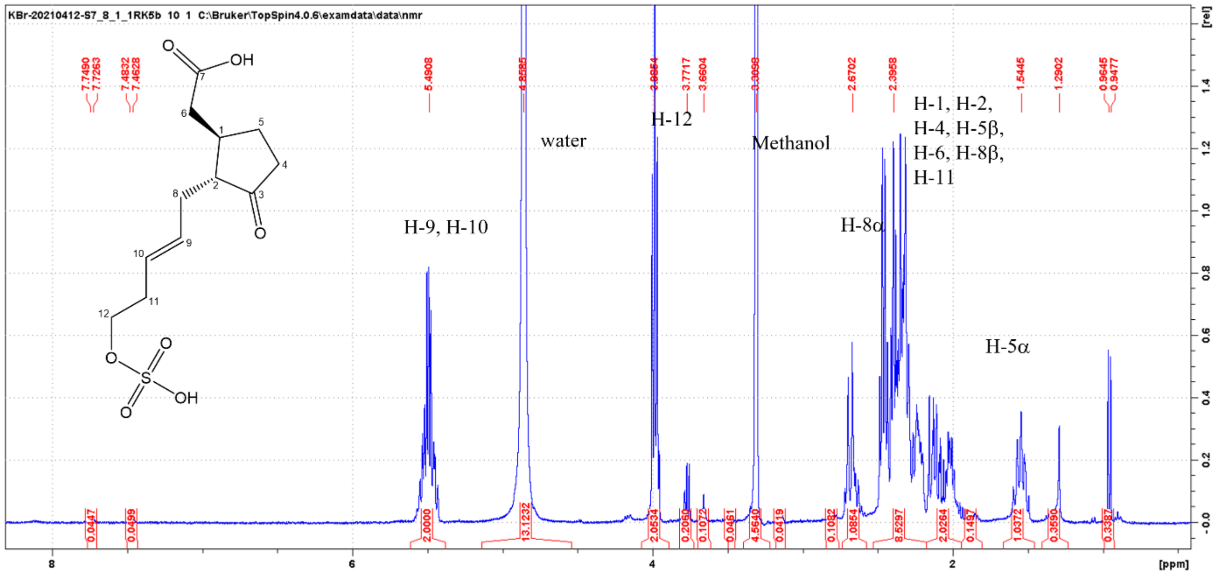

Figure S 4: H-NMR (MeOD) of purified (SPE and HPLC) sulfonation product after modified workup.

Solvent-guided fractionation of raw cocoa:

Sample	yield (g/100g)
fraction I	39.3
fraction II	1.3
fraction III	4.0
fraction IV	11.7
fraction V	43.7

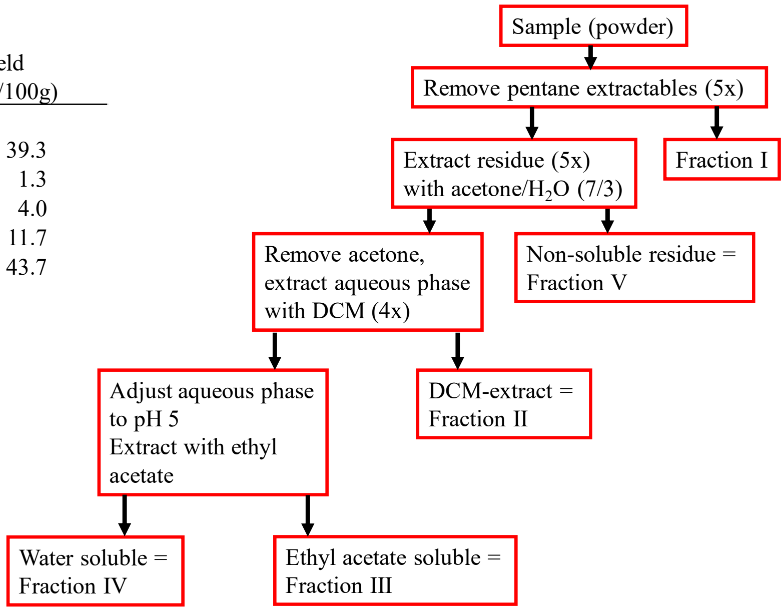

Figure S 5: Overview of solvent-guided fractionation of raw cocoa beans

GPC fractionation:

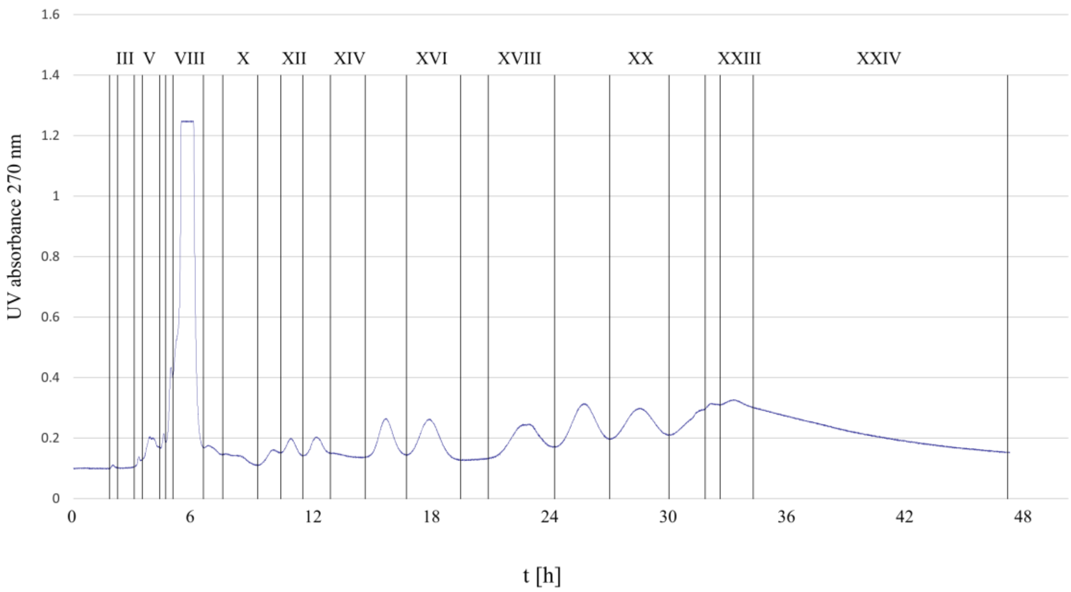

Figure S 6: Gel permeation chromatography (GPC) fractionation of the aqueous extract of raw dried cocoa beans.

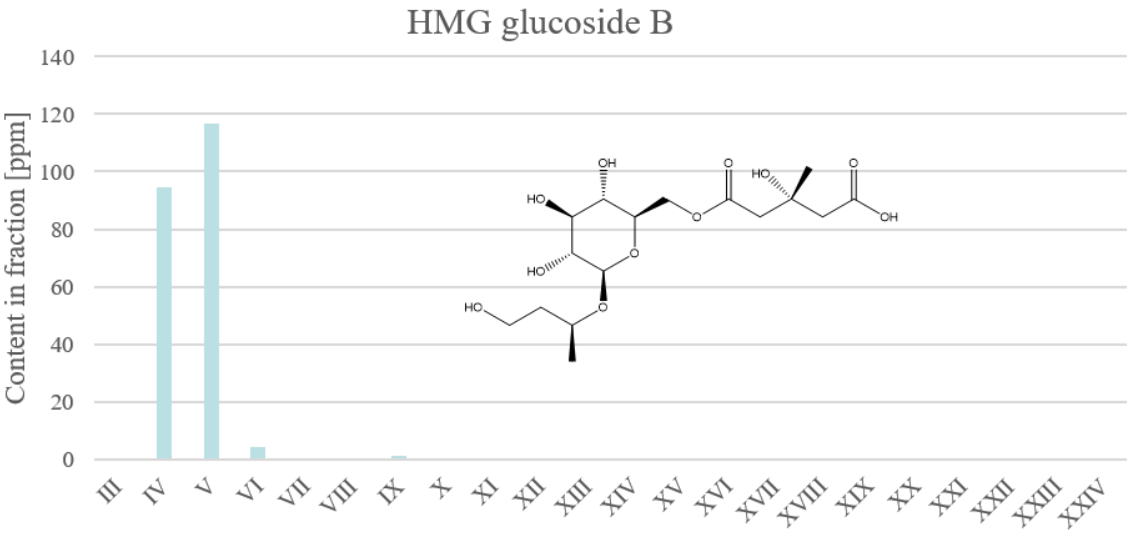

Figure S 7: Content of HMG glucoside B determined by external calibration with HMG glucoside A standard in lyophilized GPC fractions

SPE-subfractionation of GPC fraction V:

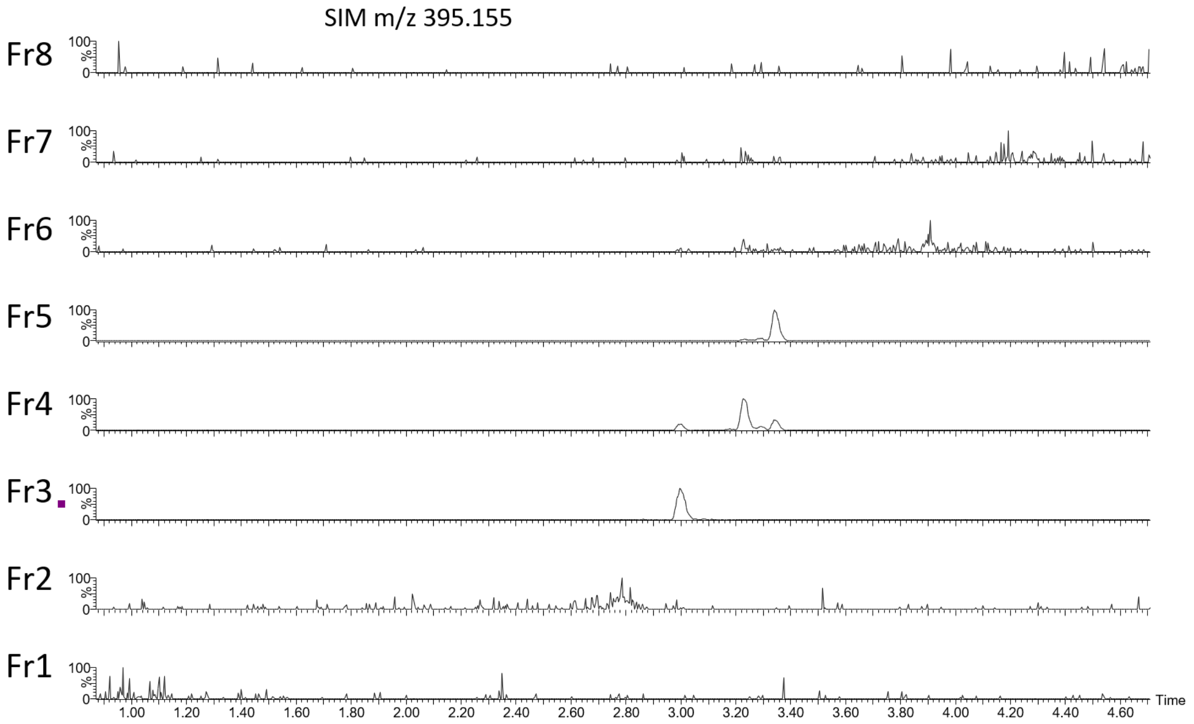

Figure S 8: Single Ion traces of UPLC-ToF-MS measurements (ESI neg) of SPE-subfractions of GPC fraction V.

Identification of HMG gluc B

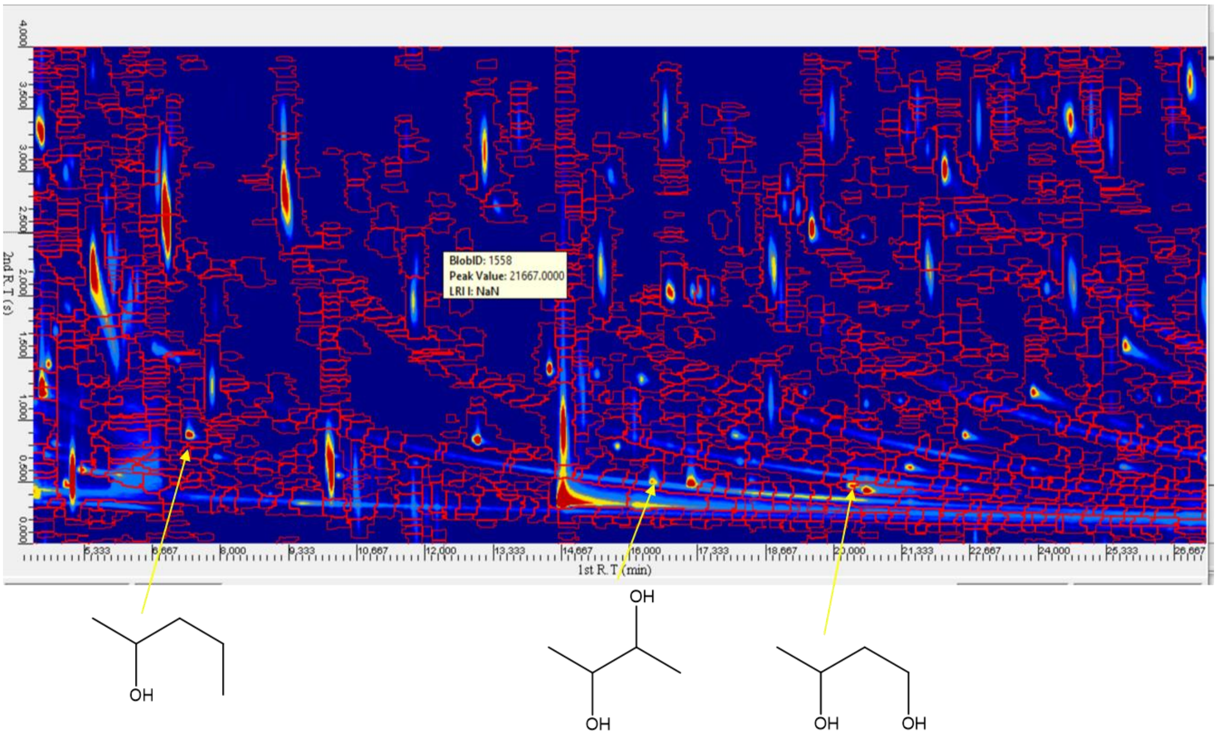

Figure S 9: Mix of 1,3-butandiol, 2,3-butandiol and 2-pentanol standards in buffer. Two-dimensional plot (1st separation horizontal on axis, 2nd separation vertical on axis) with high TIC (total ion current) intensities colored red and low intensities colored blue. Peaks (in red, so-called "Blobs") were identified by retention indices and EI-MS reference libraries (NIST library).

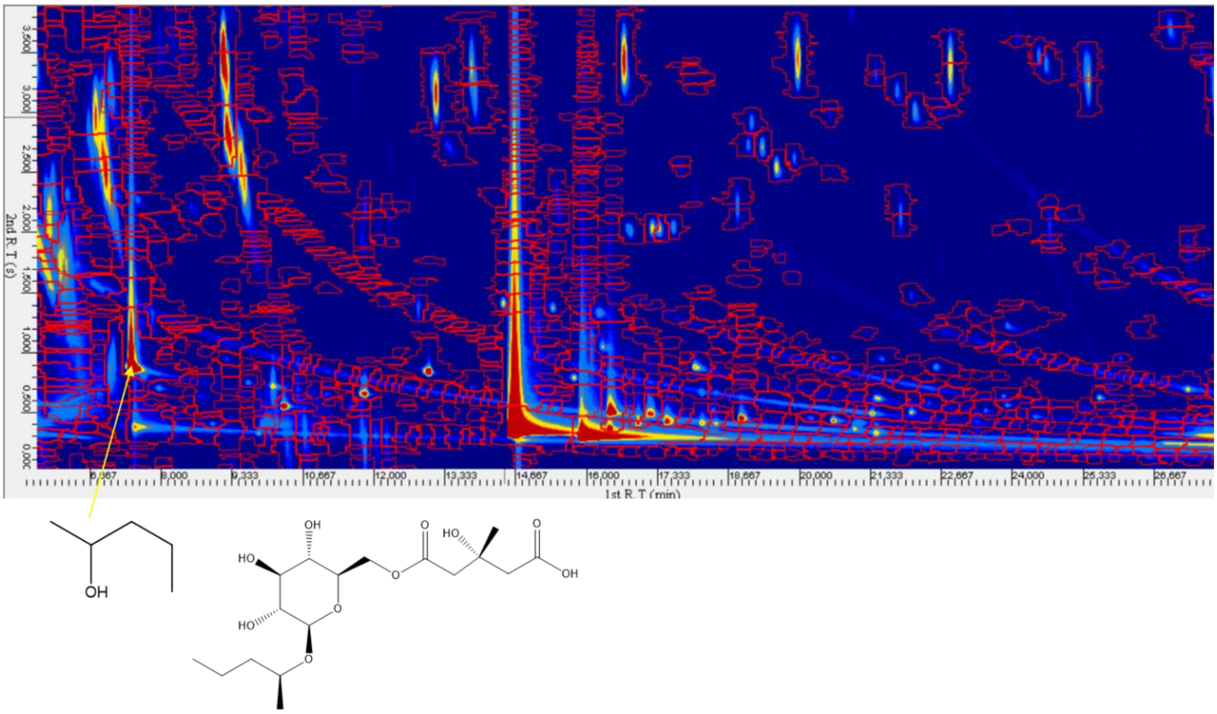

Figure S 10: Hydroxymethylglutaryl glucoside standard after hydrolysis. Two-dimensional plot (1st separation horizontal on axis, 2nd separation vertical on axis) with high TIC (total ion current) intensities colored red and low intensities colored blue. Peaks (in red, so-called "Blobs") were identified by retention indices and EI-MS reference libraries (NIST library).

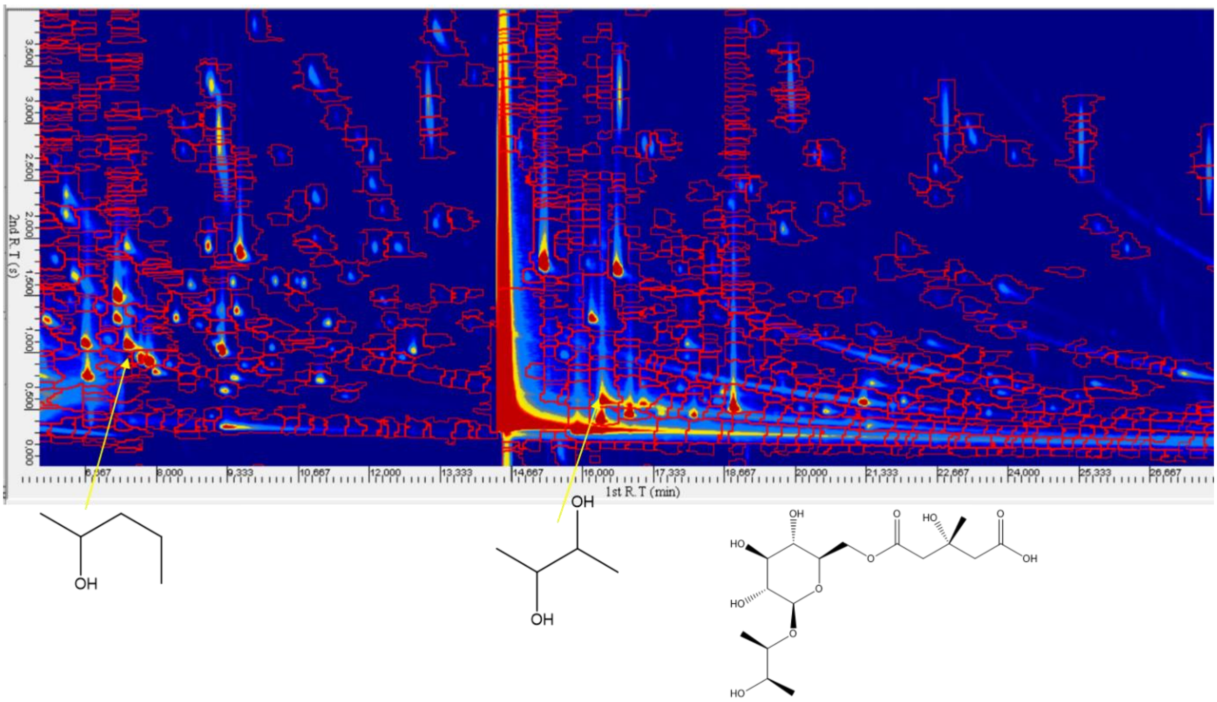

Figure S 11: Fraction GPCV/SPE4 after hydrolysis. Two-dimensional plot (1st separation horizontal on axis, 2nd separation vertical on axis) with high TIC (total ion current) intensities colored red and low intensities colored blue. Peaks (in red, so-called "Blobs") were identified by retention indices and EI-MS reference libraries (NIST library).

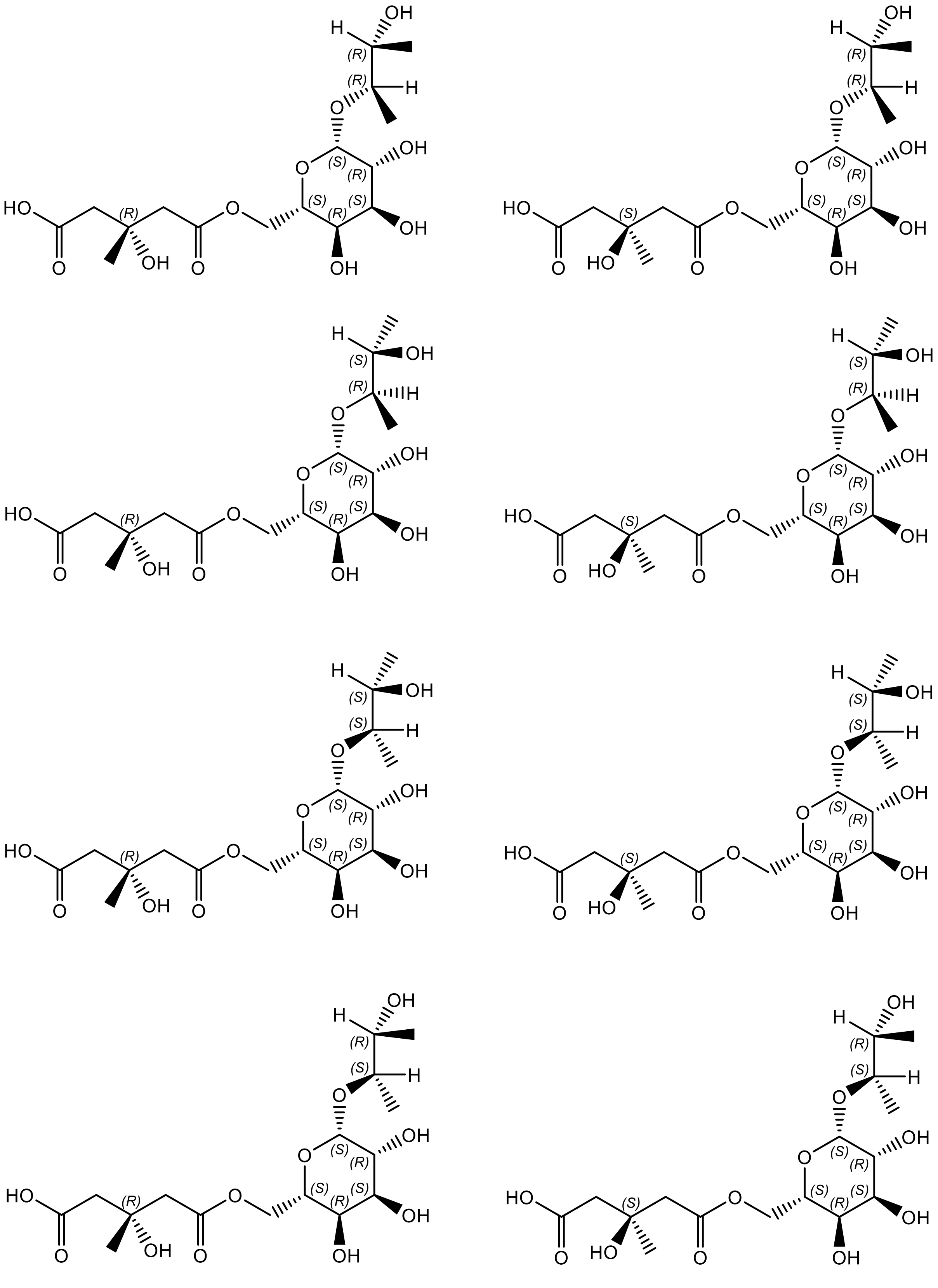

Figure S 12: postulated HMG gluc B isomer (α-L-galactopyranoside)

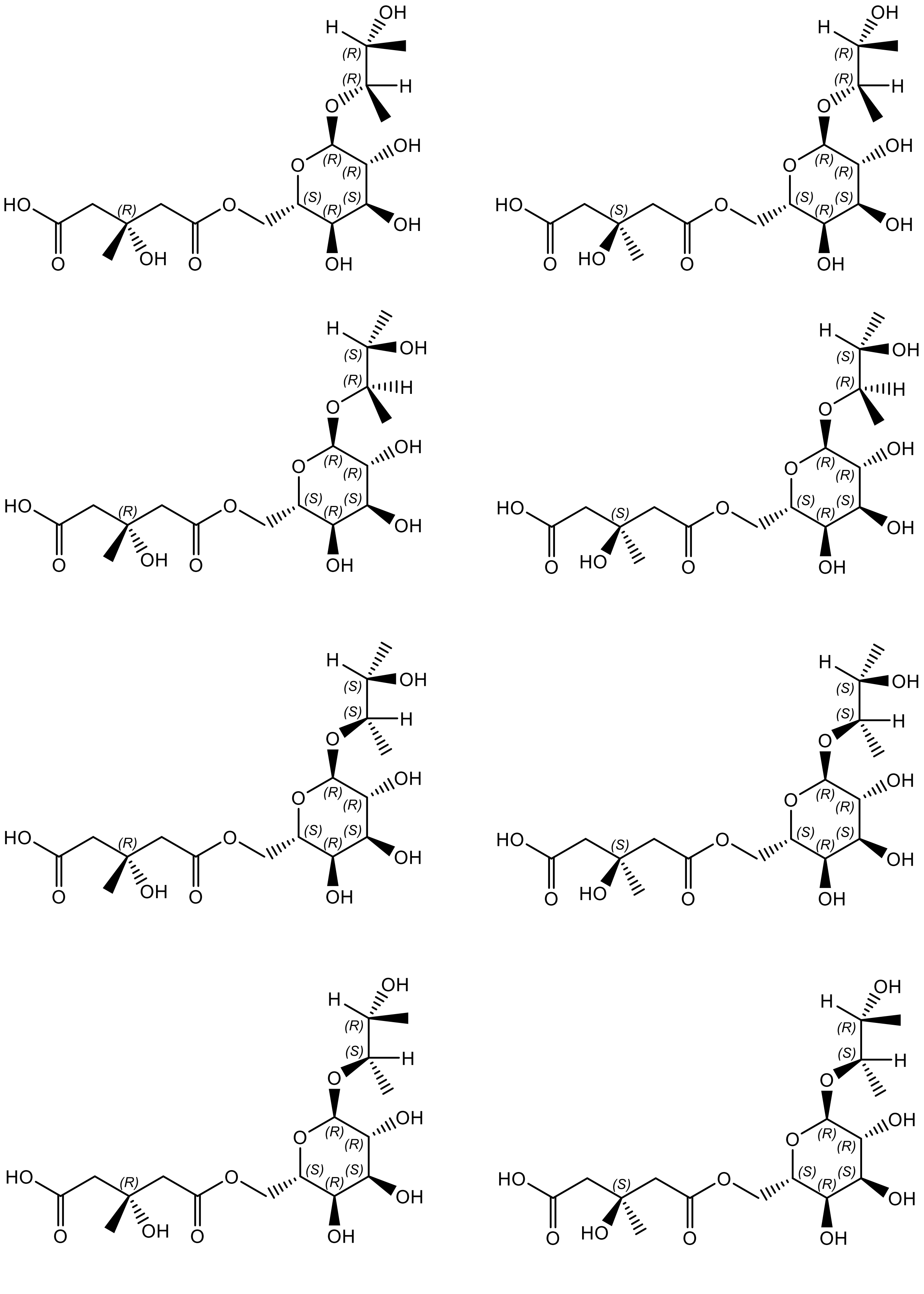

Figure S 13: postulated HMG gluc B isomers (β-D-galactopyranoside)

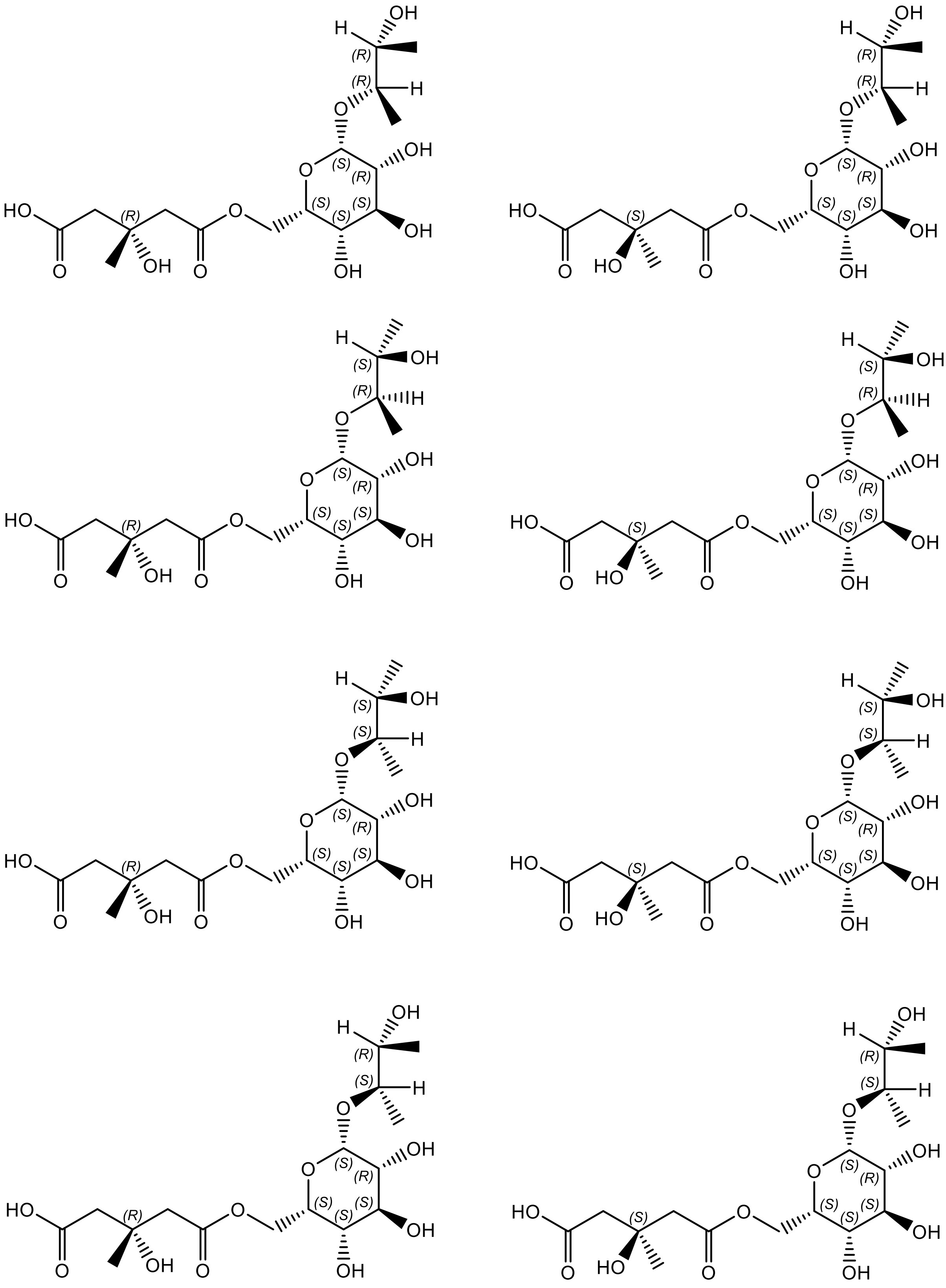

Figure S 14: postulated HMG gluc B isomers (α-L-glucopyranoside)

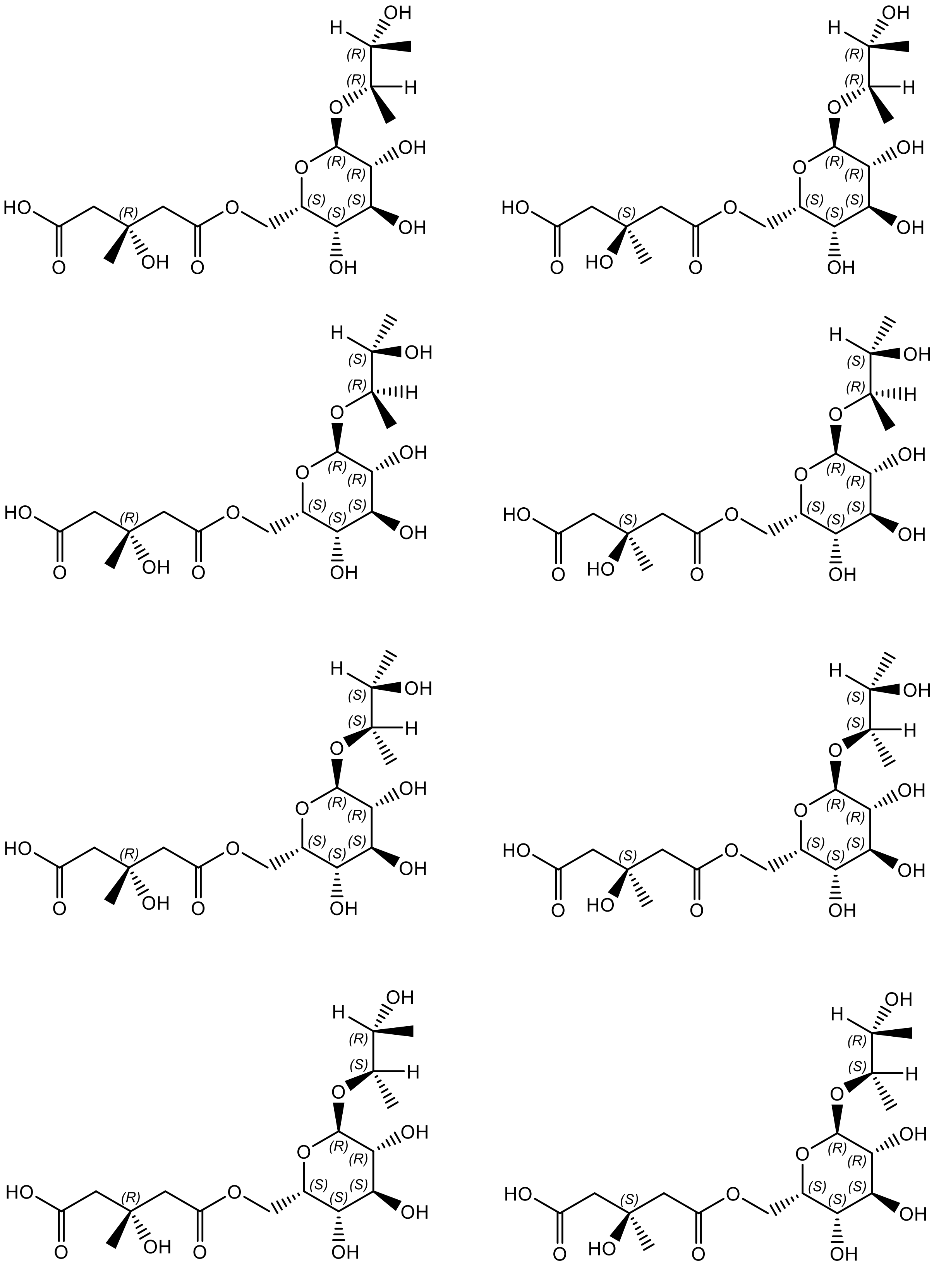

Figure S 15: postulated HMG gluc B isomers (β-D-glucopyranoside)

Identification of THOA isomers

Spiking of commercial standard compounds to Cocoa liquor extract (Extract prepared from sample #94)

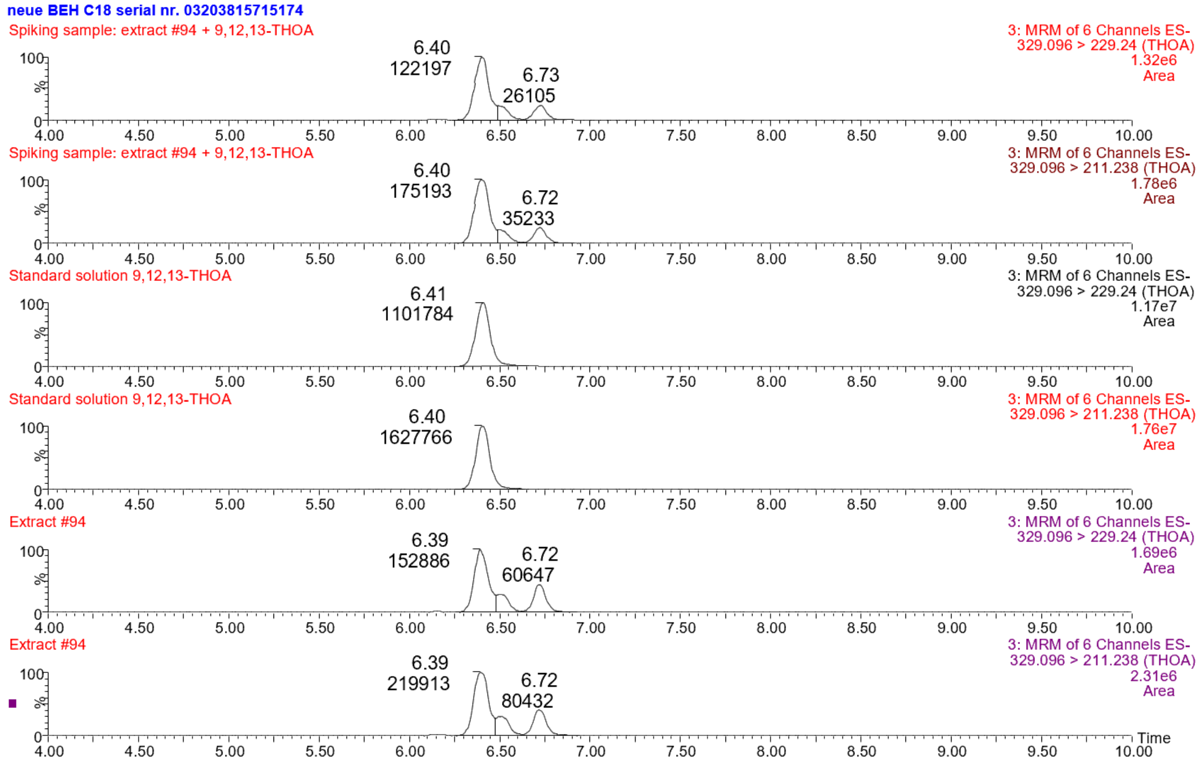

Figure S 16: MRMs measured for cocoa liquor sample #94 before and after spiking (1:1; v/v) with commercial standard compound of 9,12,13-S,S,S-Trihydroxyoctadec-10E-enoic acid.

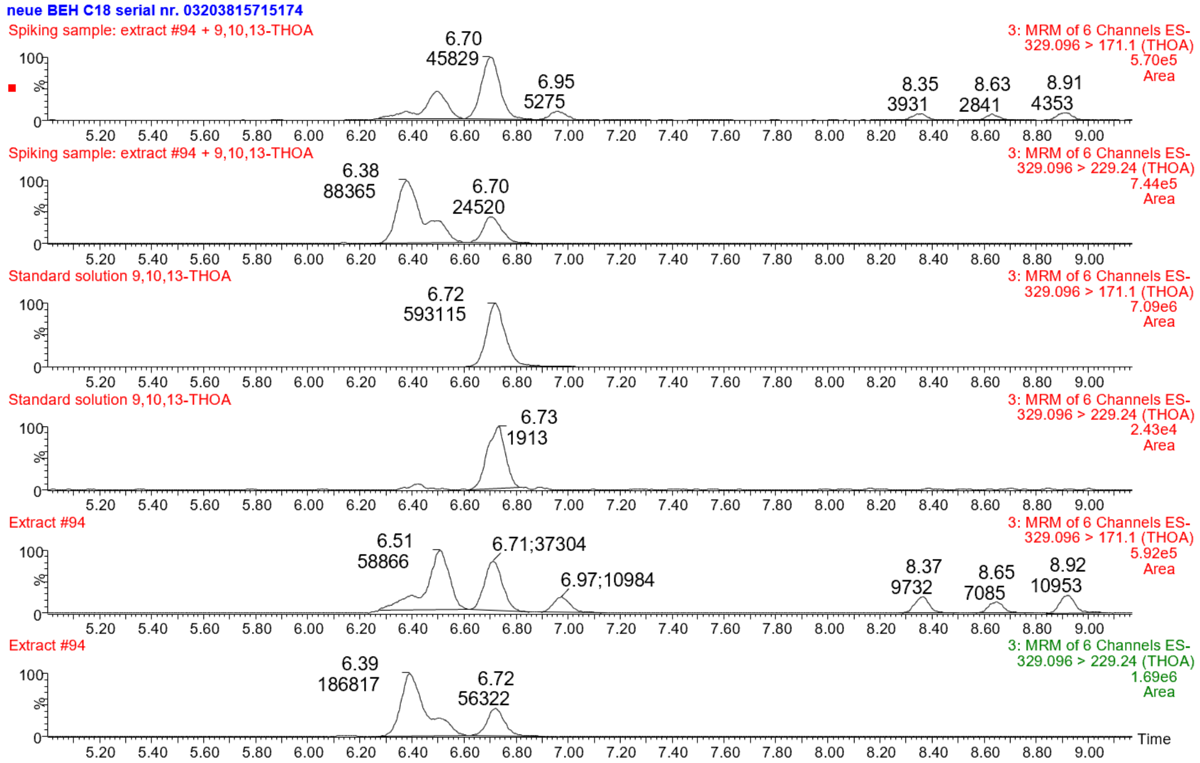

Figure S 17: MRMs measured for cocoa liquor sample #94 before and after spiking (1:1; v/v) with commercial standard compound of 9,10,13-S,S,S-Trihydroxyoctadec-11E-enoic acid.

Summary: Chromatograms of all identified marker compounds

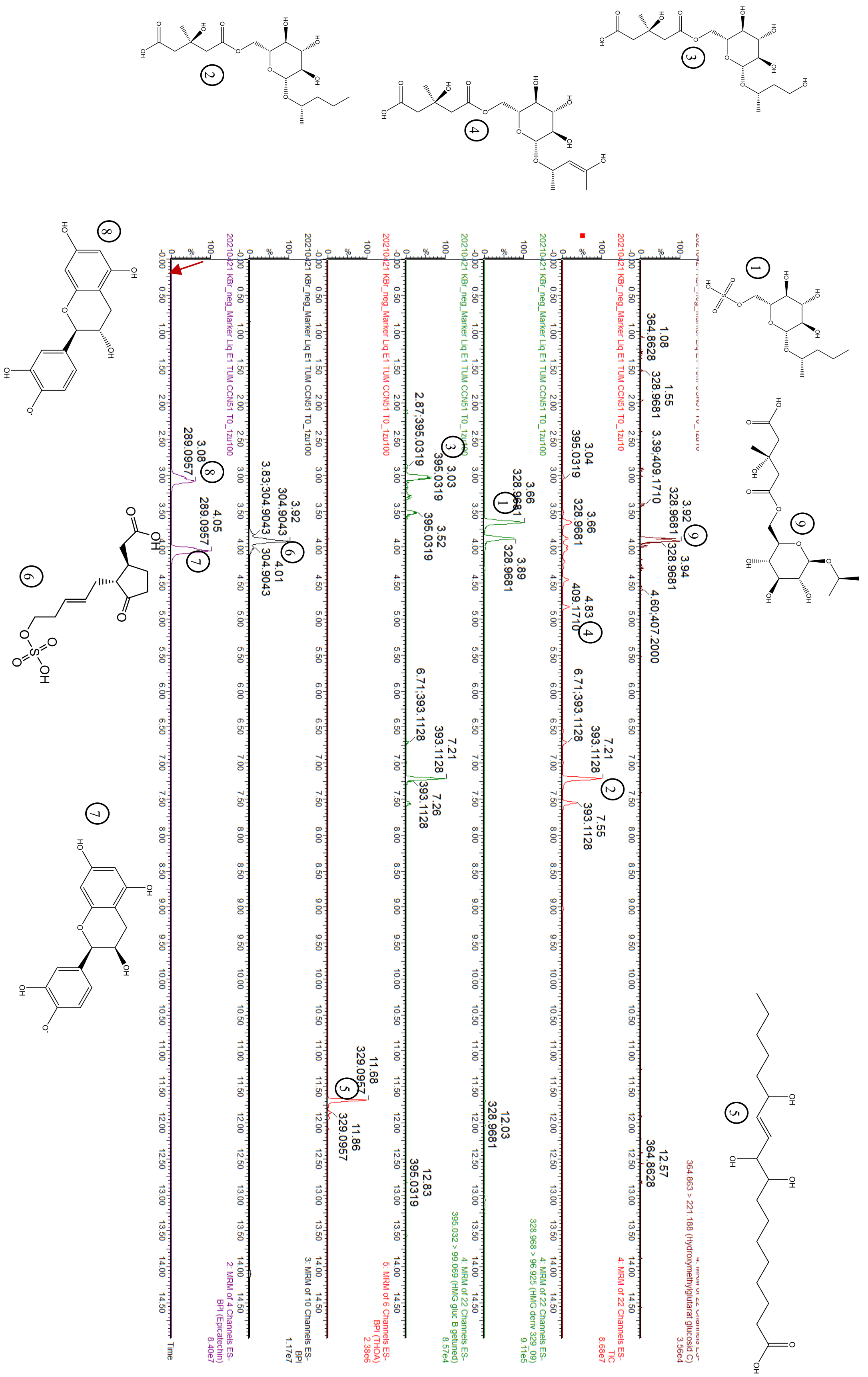

Figure S 18: MRM chromatograms of marker compounds, measured in the extract of raw cocoa bean liquor at the waters system after compound optimization.

Taste contribution of marker compounds

Results of Taste analysis

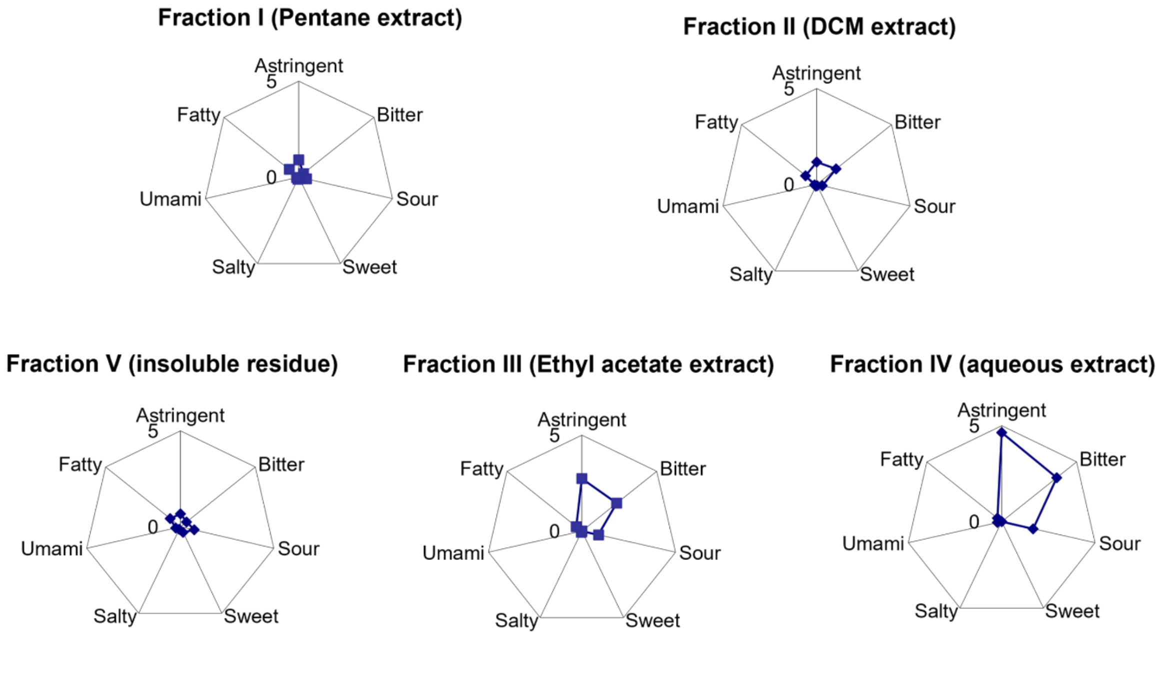

Figure S 19: Taste profiles of solvent fractions.

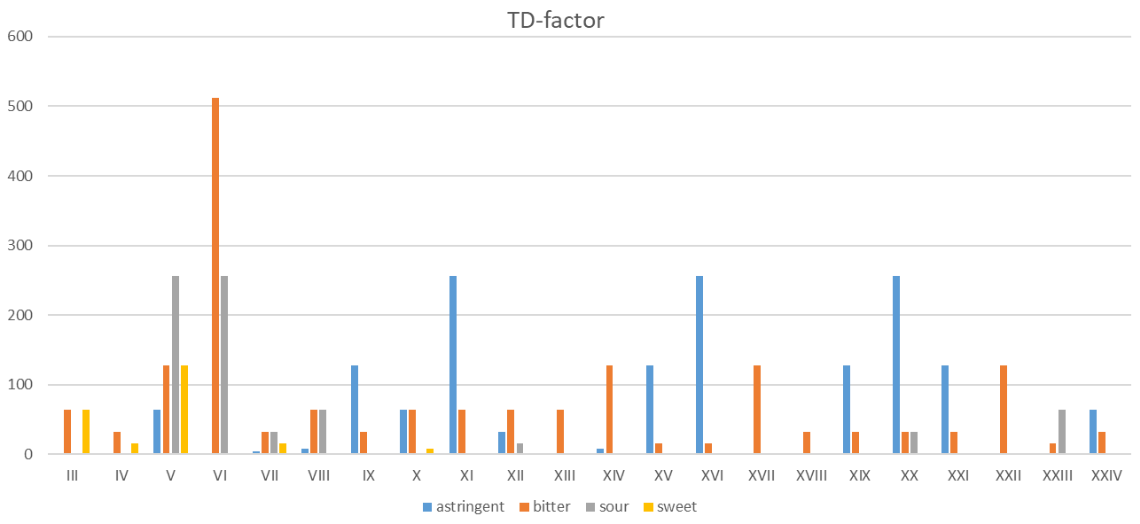

Figure S 20: Taste dilution analysis (TDA) of the GPC fractions collected. Bitterness, Sourness and Sweetness were evaluated in a conventional TDA whereas Astringency was evaluated by half-tongue test.

Quantification of known taste-active compounds in GPC-fractions

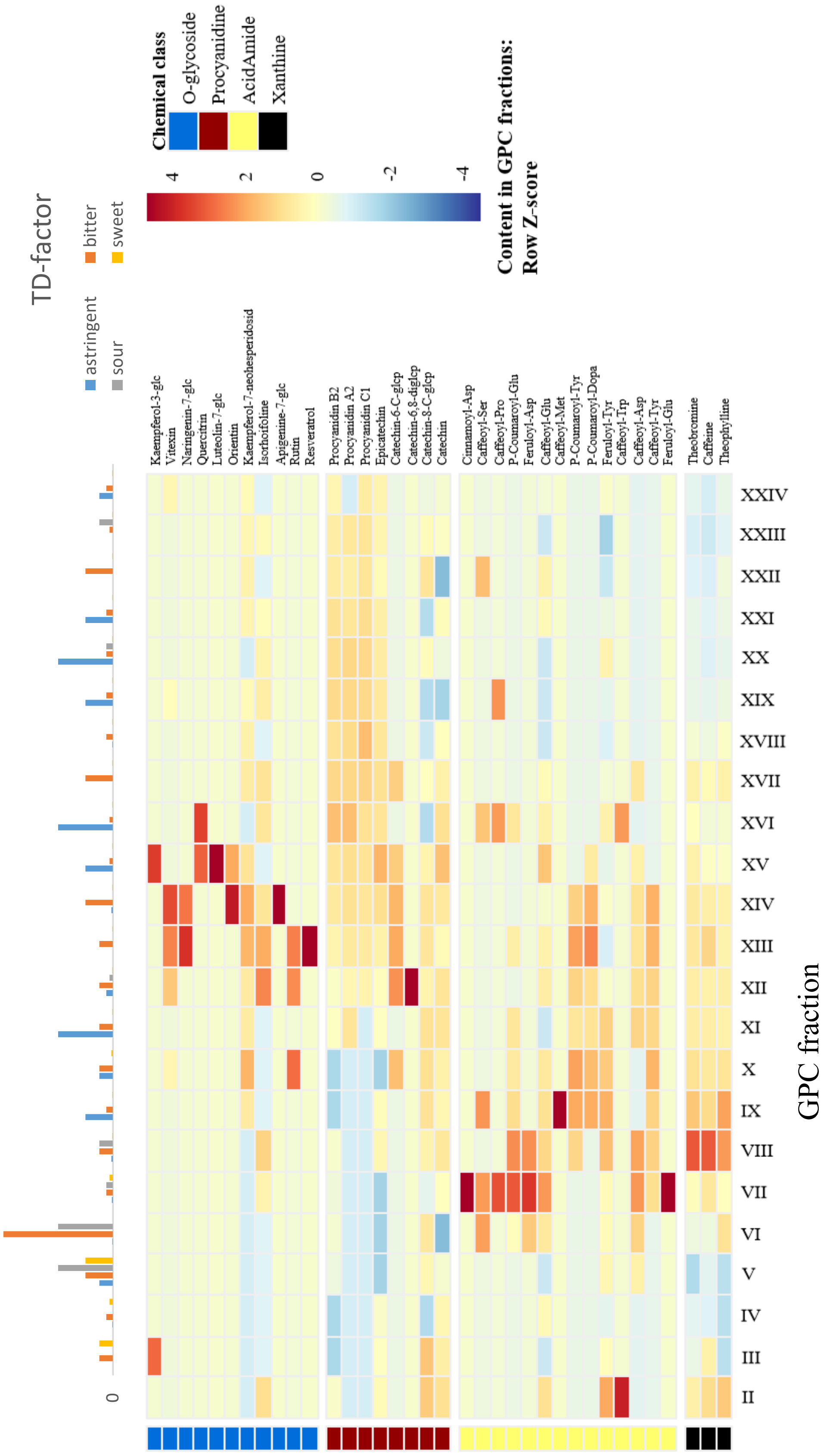

Figure S 21: TD factors and heatmap of normalized contents of known taste active compounds in cocoa quantified in GPC-fractions.

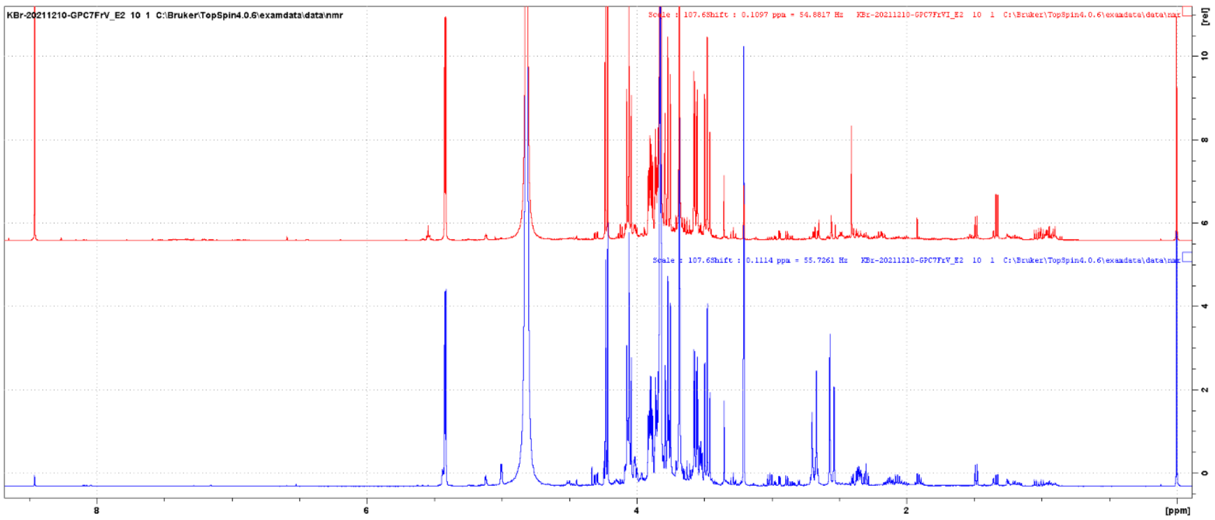

Figure S 22: ^{1}H-qNMR spectra of GPC fractions V (bottom, blue) and VI (top, red) measured in NMR-buffer (pH 7).

Table S 6: Taste-active compounds identified and quantified by ^{1}H NMR spectroscopy; identification was performed by library screening[5], quantification only if within specifications (signal/noise ratio > 3 ; n.q.: not quantified).

Compound	Taste quality	Threshold [µmol/kg]	GPC fraction V		GPC fraction VI	
			content [mmol/kg]	DoT factor	content [mmol/kg]	DoT factor
sucrose	sweet	6800 [a]	1362	200	1601	235
L-alanine	sweet	12000 [b]	46.0	3.84	71.1	5.93
formic acid	sour/salty	4338 [c]	8.75	2.02	222	51.2
citric acid	sour/salty	2600 [c]	546	210	144	55.5
acetic acid	sour/salty	3100 [b]	n.q.	n.q.	26.3	8.47
lactic acid	sour/salty	11890 [d,e]	27.1	2.28	94.0	7.90
succinic acid	sour/salty	900 [c]	n.q.	n.q.	93.6	104
L-aspartic acid	umami	600 [d]	n.q.	n.q.	n.q.	n.q.
glutaric acid	sour/salty	3125 [c]	n.q.	n.q.	n.q.	n.q.

[a] Petty et al. 2020
[b] Warendorf 1991
[c] Hufnagel et al. 2008
[d] Toelstede et al. 2008
[e] Sodium salt

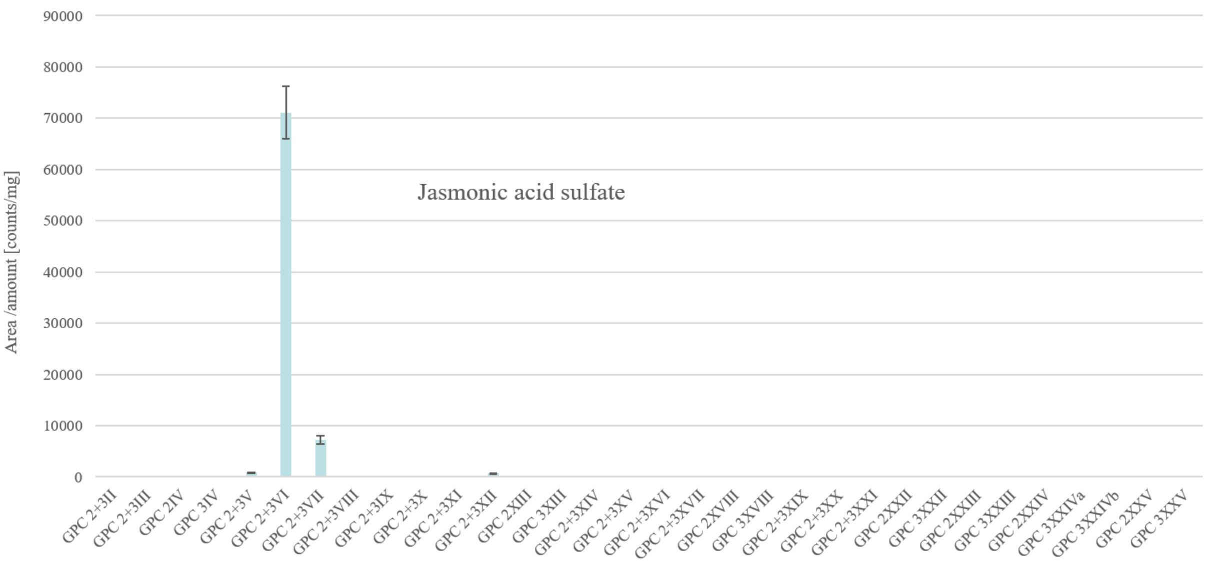

Figure S 23: Areas of HOJA sulfate normalized to natural ratios of GPC fractions, triple measurement

Mass spectra (UPLC-ESI-qTOF-MS) of standard compounds

HMG gluc C

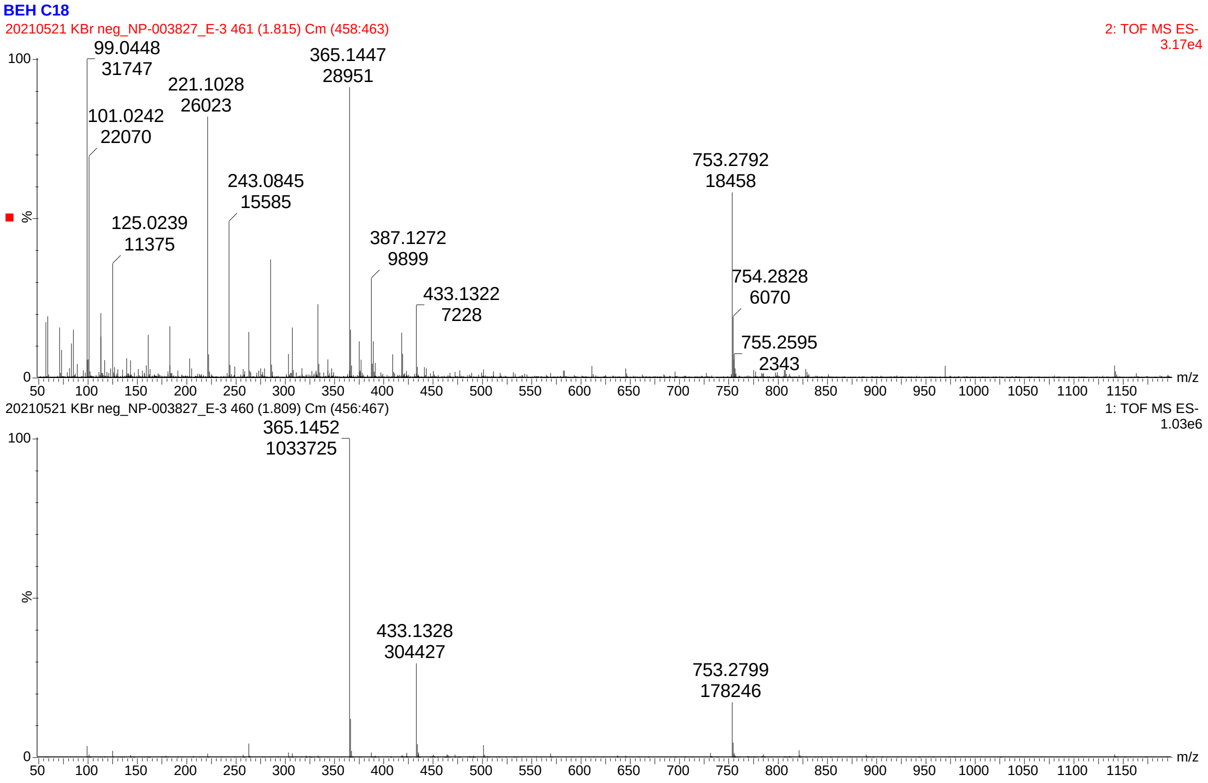

Figure S24: High collision energy MS^E (top) and low collision energy MS^E (bottom) of standard solution.

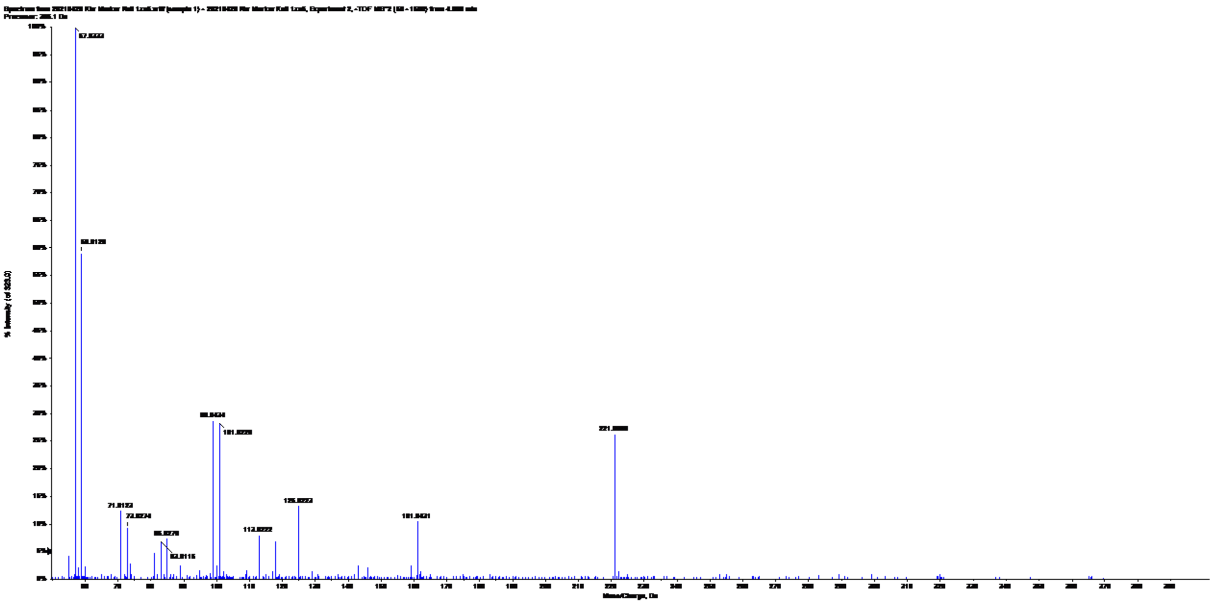

Figure S25: MS^2 spectrum of standard solution.

HMG gluc A

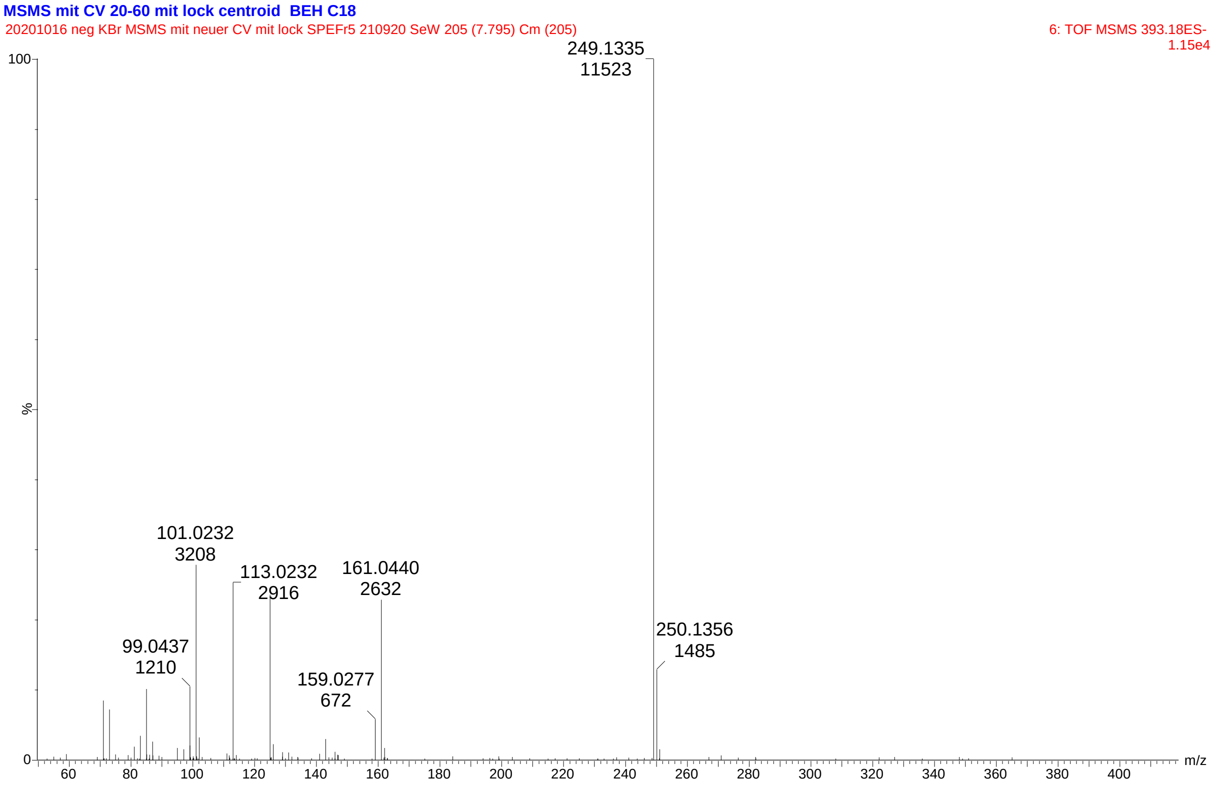

Figure S26: ToF-MS/MS spectrum with precursor m/z 393.175 of SPE-enriched acetone/water-extract of raw cocoa beans.

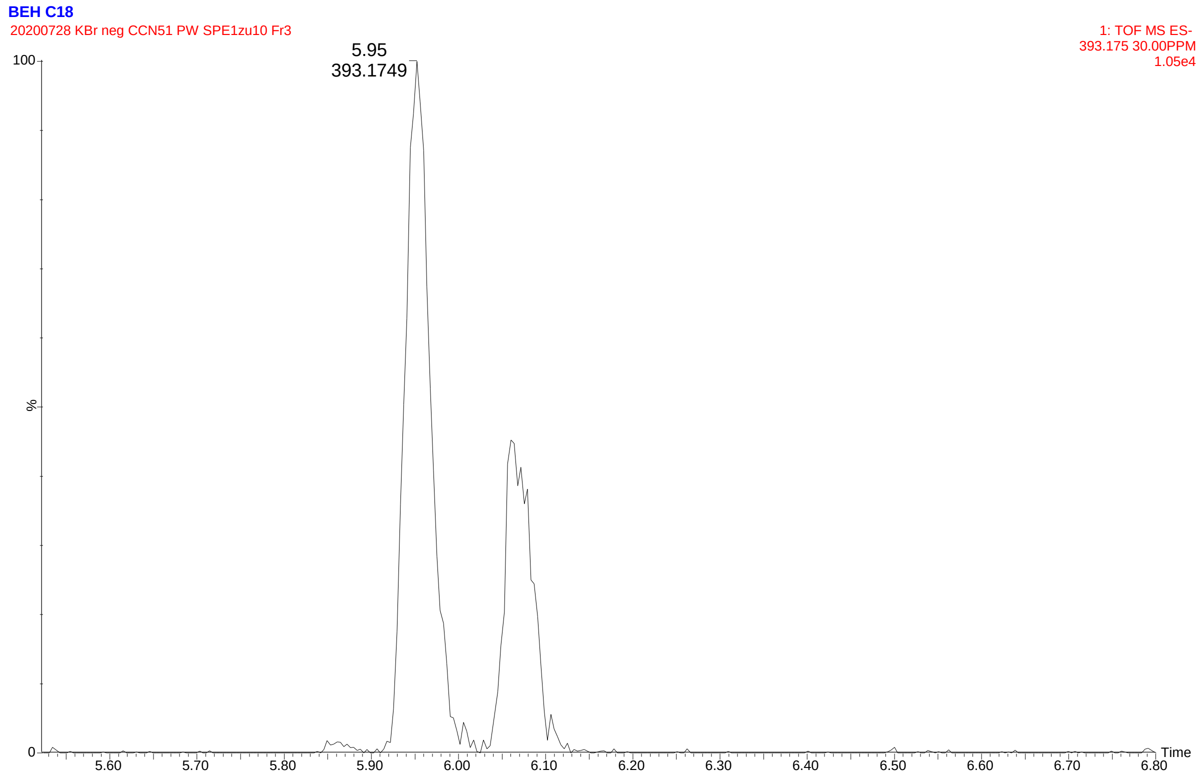

Figure S27: Extracted trace of m/z 393.175 (+/-30 ppm) from UPLC-ToF-MS chromatogram (in MS^E mode) with low collision energy, measured in SPE enriched acetone/water-extract of raw cocoa beans.

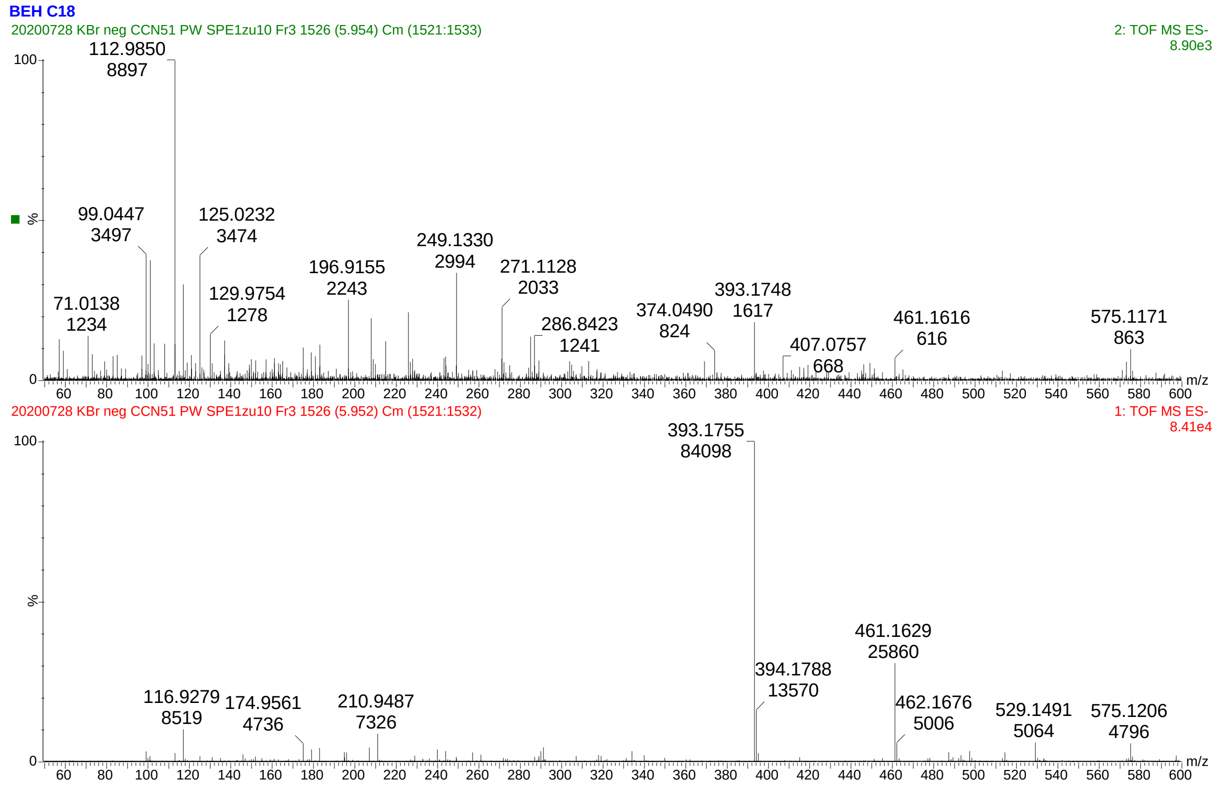

Figure S28: ToF-MS^E spectra of first peak (5.95 min) with high CE (top) and low CE (bottom).

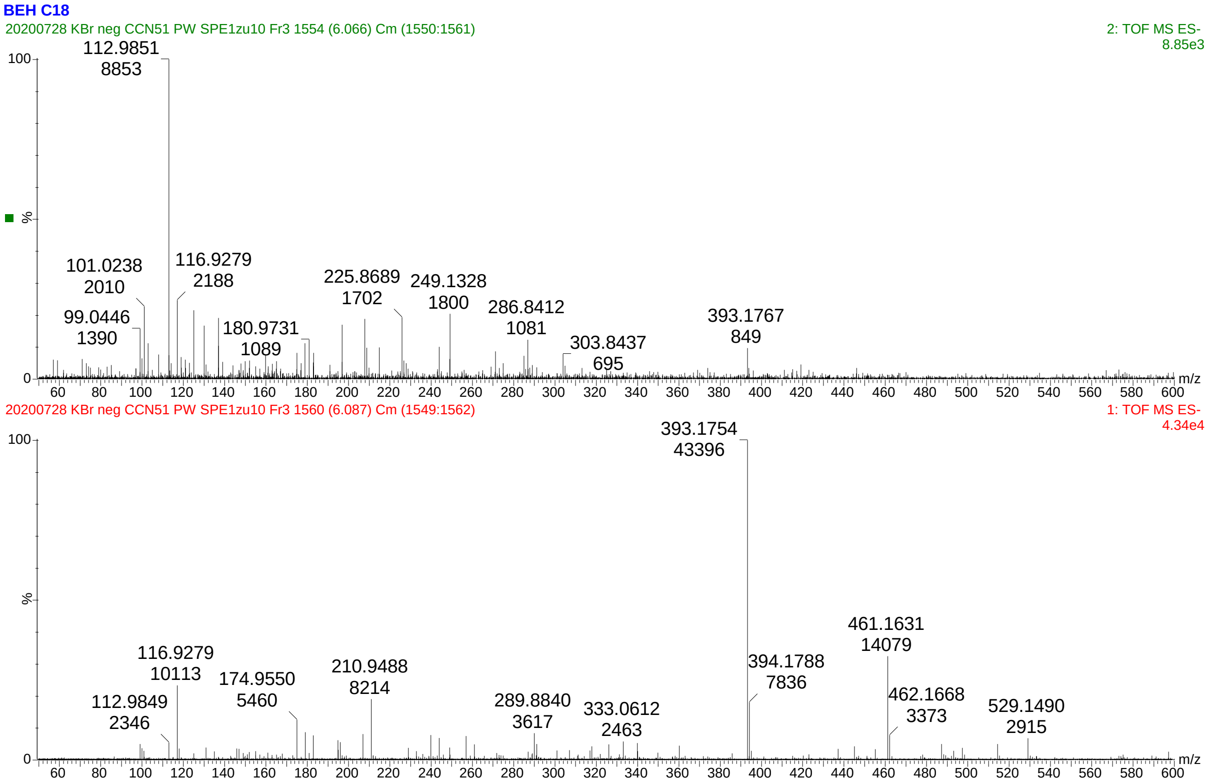

Figure S29: ToF-MS^E spectra of second peak (6.09 min) with high CE (top) and low CE (bottom).

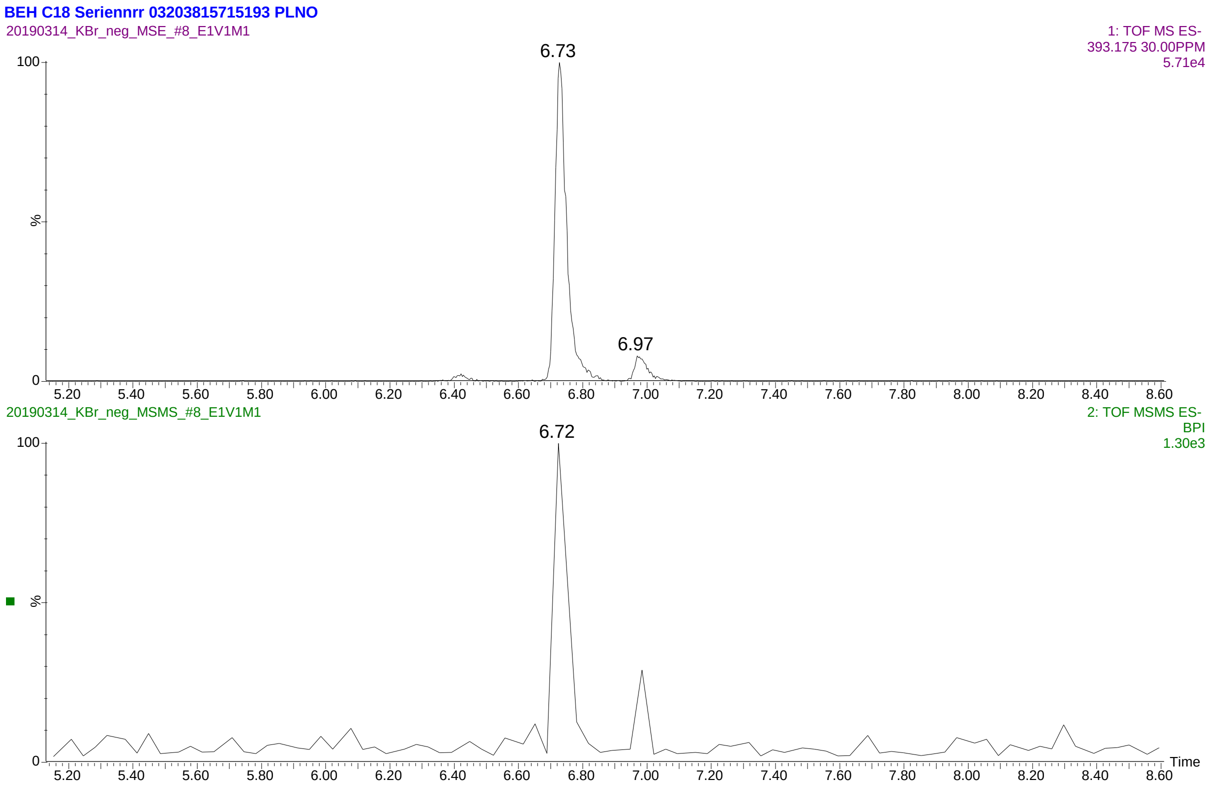

Figure S30: Extracted trace of m/z 393.175 (+/-30 ppm) from UPLC-ToF-MS chromatogram (top, in MS^E mode) with low collision energy and BPI chromatogram of UPLC-ToF-MS run (bottom, in MS^2 scan mode) of methanol/water extract from cocoa sample #8.

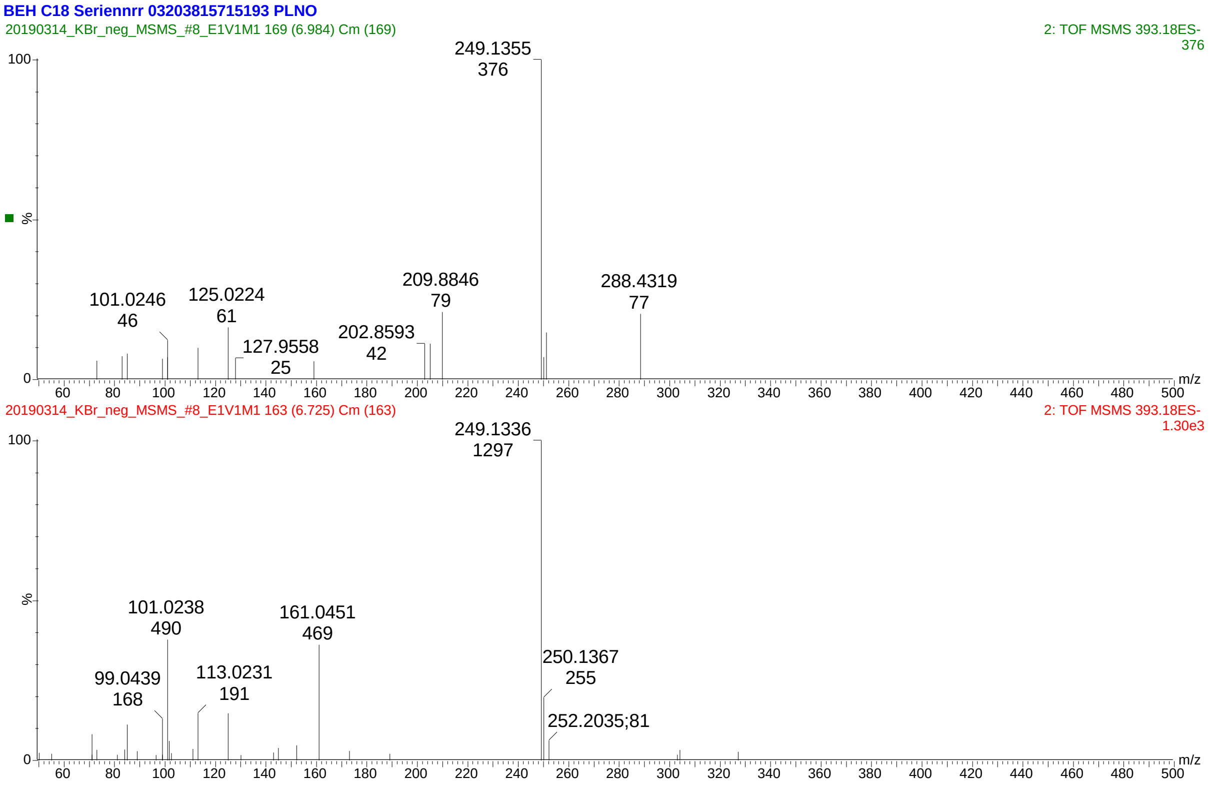

Figure S31: ToF-MS/MS spectra with precursor m/z 393.175 of peaks at 6.98 min (top) and at 6.73 min (bottom) of methanol/water extract from cocoa sample #8.

Comparison HMG gluc A and HMG gluc B:

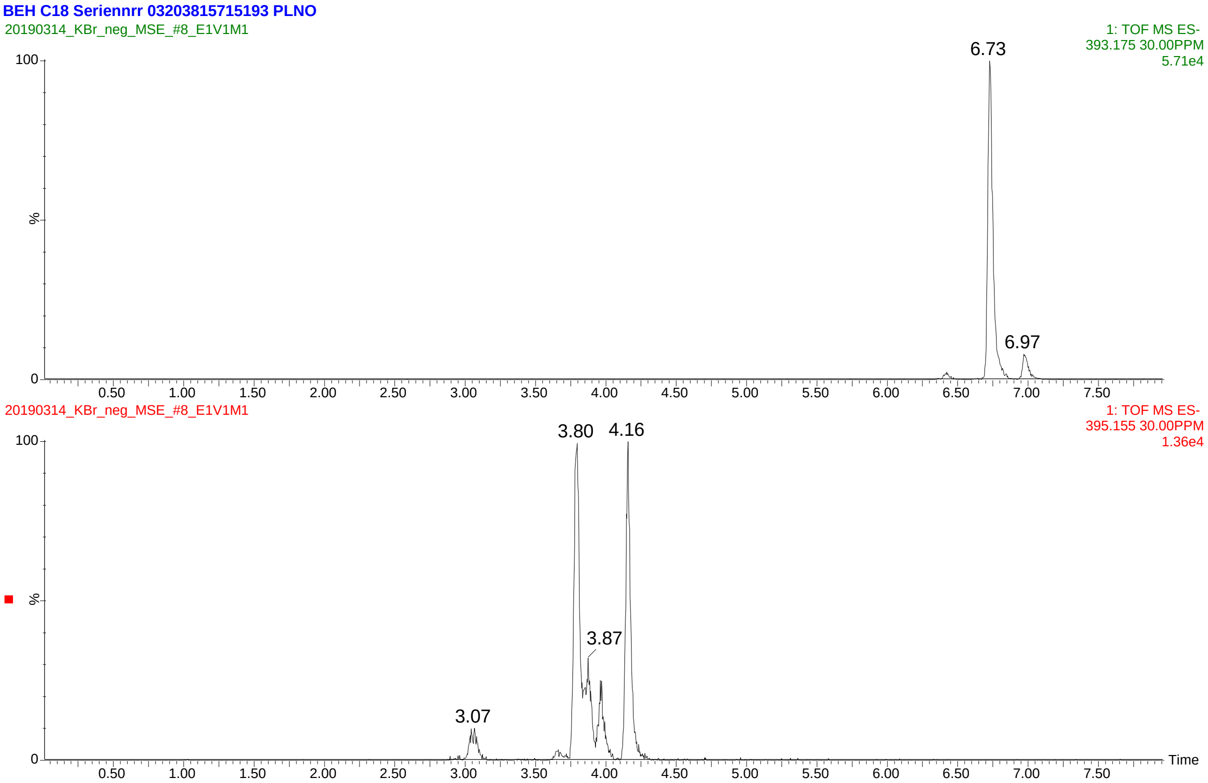

Figure S32: Extracted traces of m/z 393.175 (+/-30 ppm) (top) and of m/z 395.155 (+/- 30ppm) (bottom) from UPLC-ToF-MS chromatogram (in MS^E mode) with low collision energy of methanol/water extract from cocoa sample #8

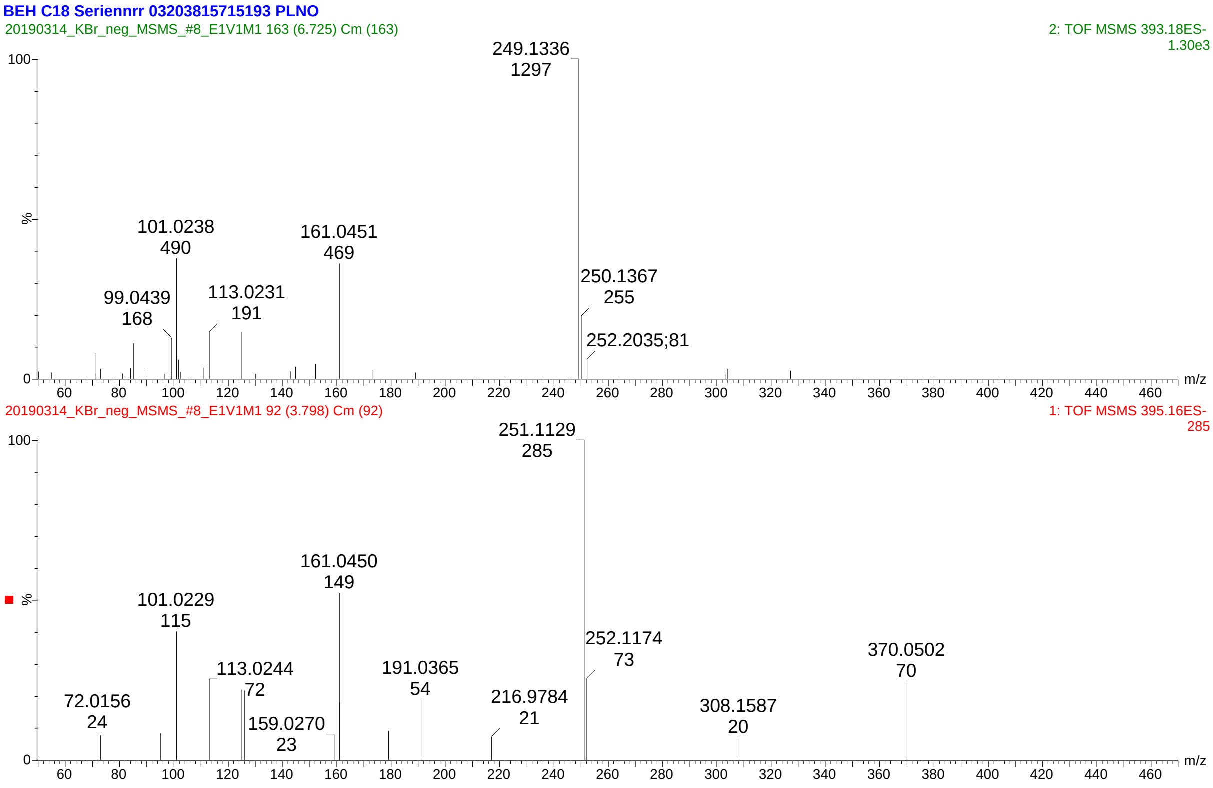

Figure S33: ToF-MS/MS spectra with precursors m/z 393.175 at 6.73 min (top) and m/z 393.155 at 3.80 min (bottom) of methanol/water extract from cocoa sample #8.

HMG gluc B

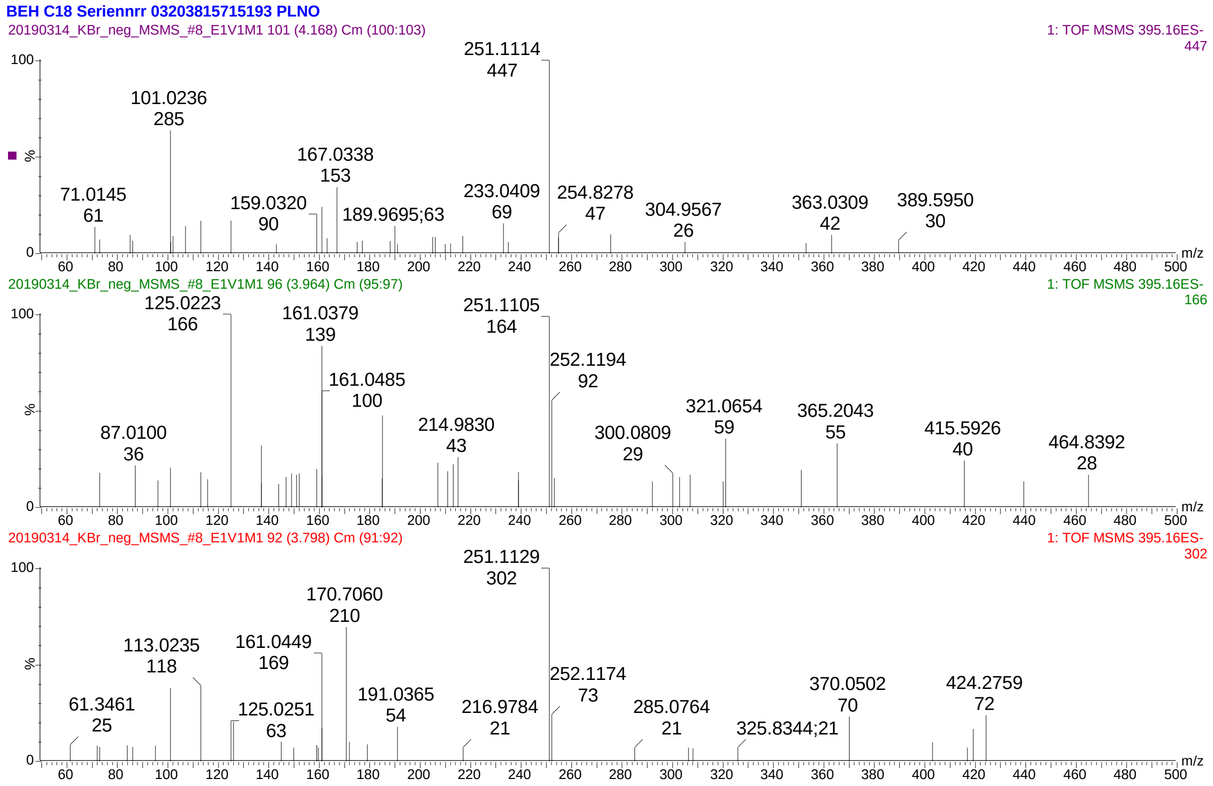

Figure S34: ToF-MS/MS spectra with precursor m/z 395.16 of peaks at 4.17 min (top), at 3.97 min (Centre) and at 3.80 min (bottom) of methanol/water extract from cocoa sample #8.

Enrichment by SPE

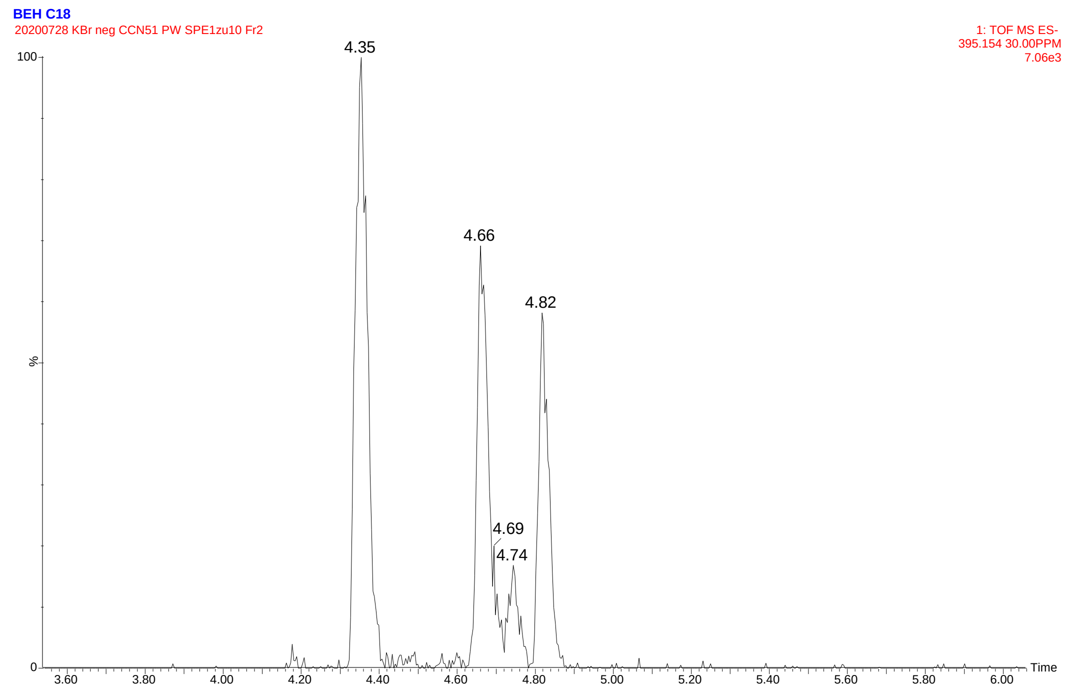

Figure S35: Extracted trace of m/z 395.154 (+/-30 ppm) from UPLC-ToF-MS chromatogram (in MS^E mode) with low collision energy, measured in SPE enriched acetone/water-extract of raw cocoa beans.

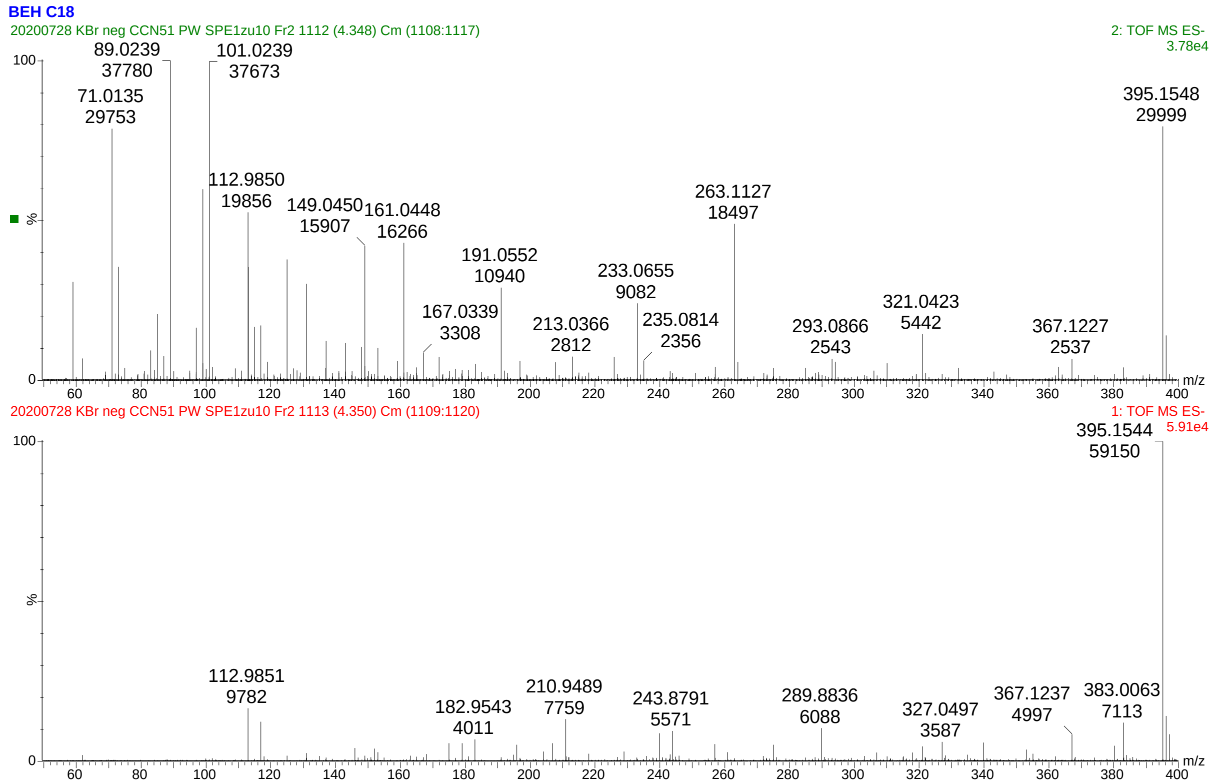

Figure S36: ToF-MS^E spectra of first peak (4.35 min) with high CE (top) and low CE (bottom).

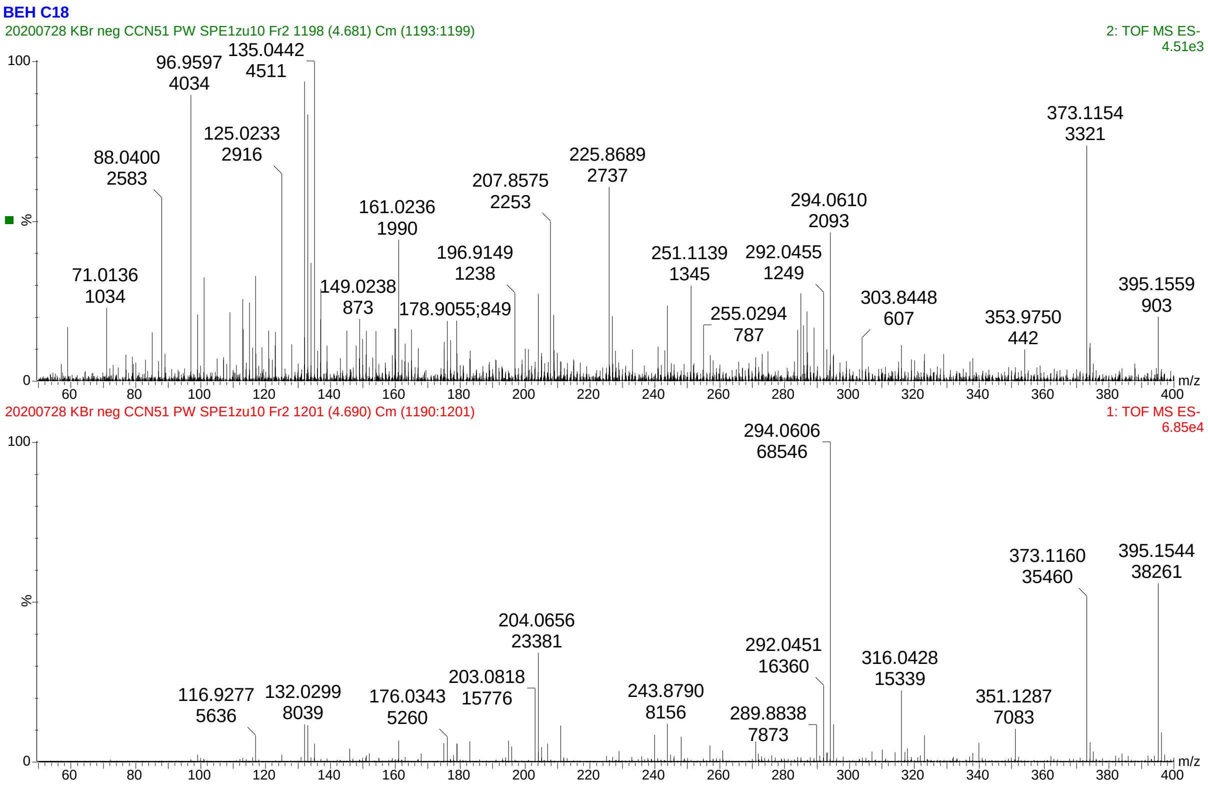

Figure S37: ToF-MS^E spectra of 1st peak (4.68 min) with high CE (top) and low CE (bottom).

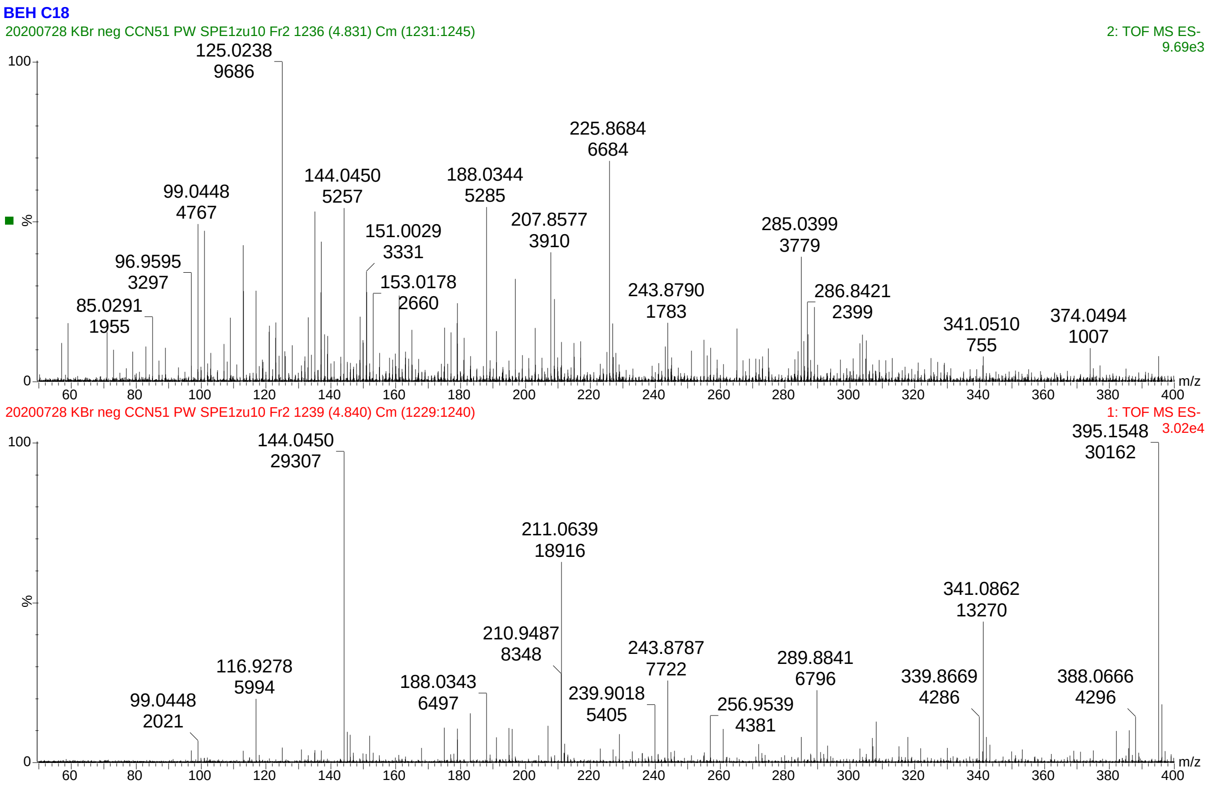

Figure S38: ToF-MS^E spectra of 2nd peak (4.84 min) with high CE (top) and low CE (bottom).

Enrichment by Prep HPLC

Isomer 1

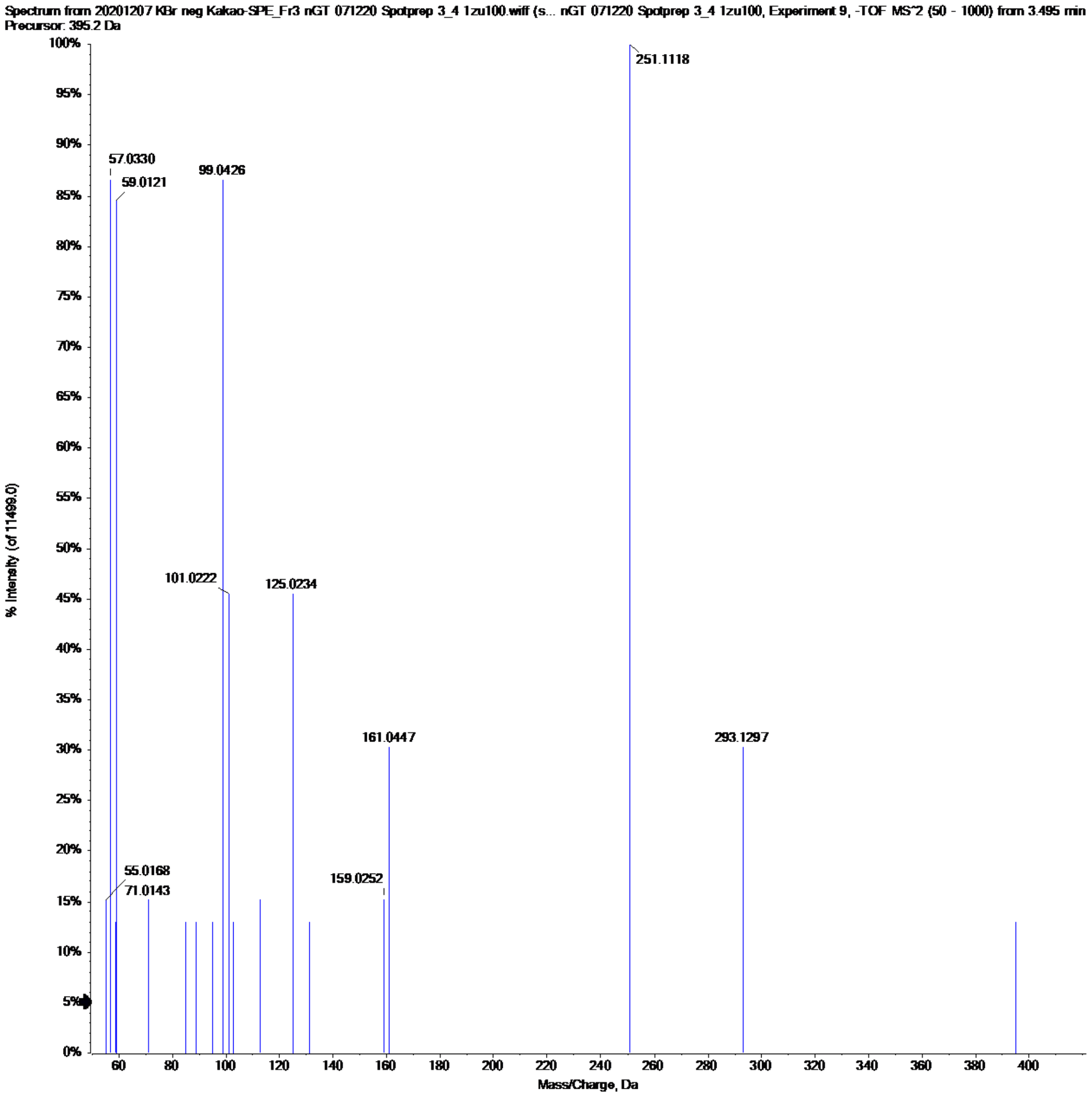

Figure S39: qToF-MS/MS spectrum of first isomer (3.5 min) with precursors m/z 395.2, measured in SPE/ HPLC enriched acetone/water-extract of raw cocoa beans.

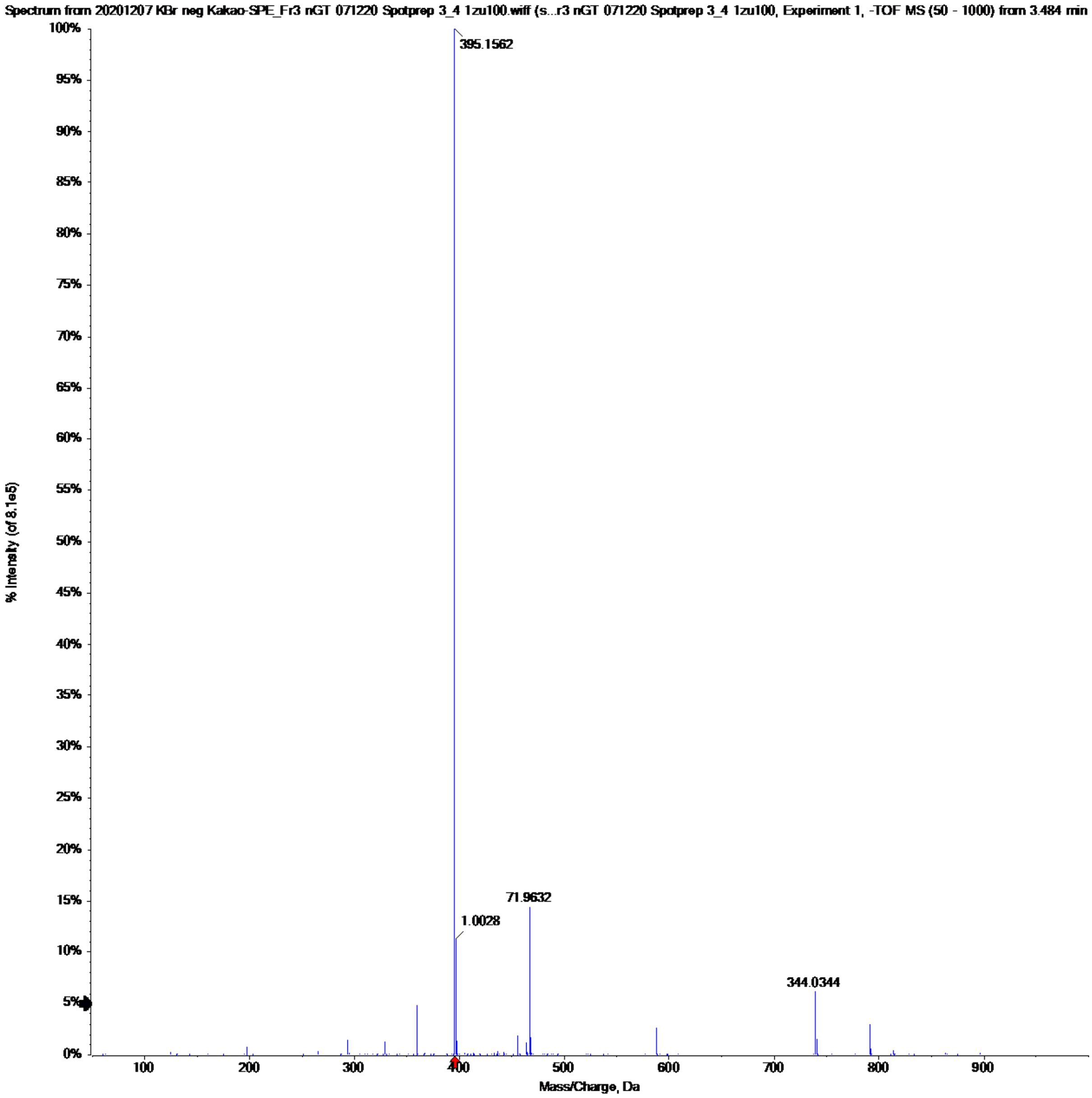

Figure S40: qToF-MS spectrum (survey scan) of first isomer (3.5 min), measured in SPE/ HPLC enriched acetone/water-extract of raw cocoa beans.

Isomer 2

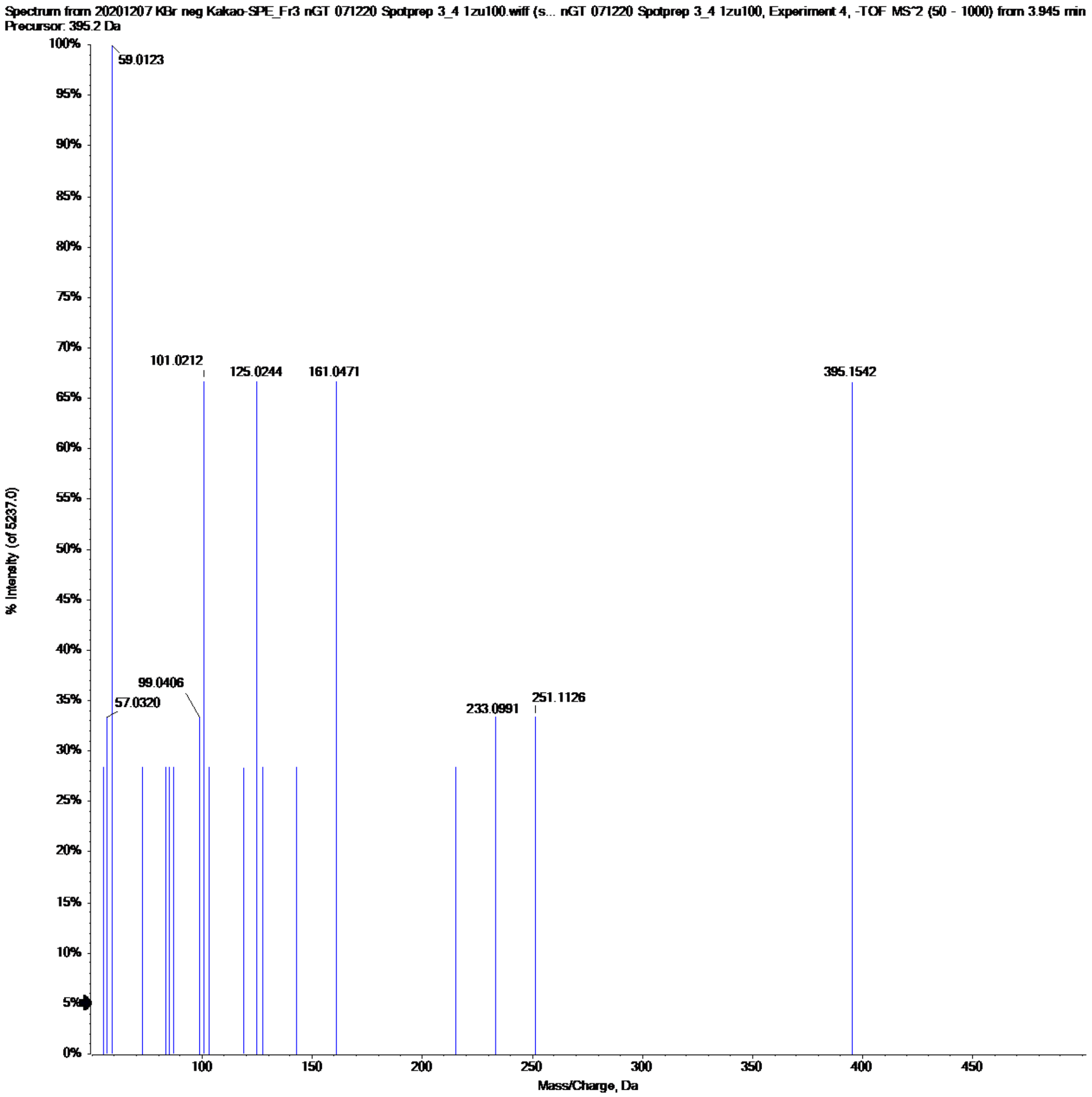

Figure S41: qToF-MS/MS spectrum of second isomer (3.9 min) with precursors m/z 395.2, measured in SPE/ HPLC enriched acetone/water-extract of raw cocoa beans.

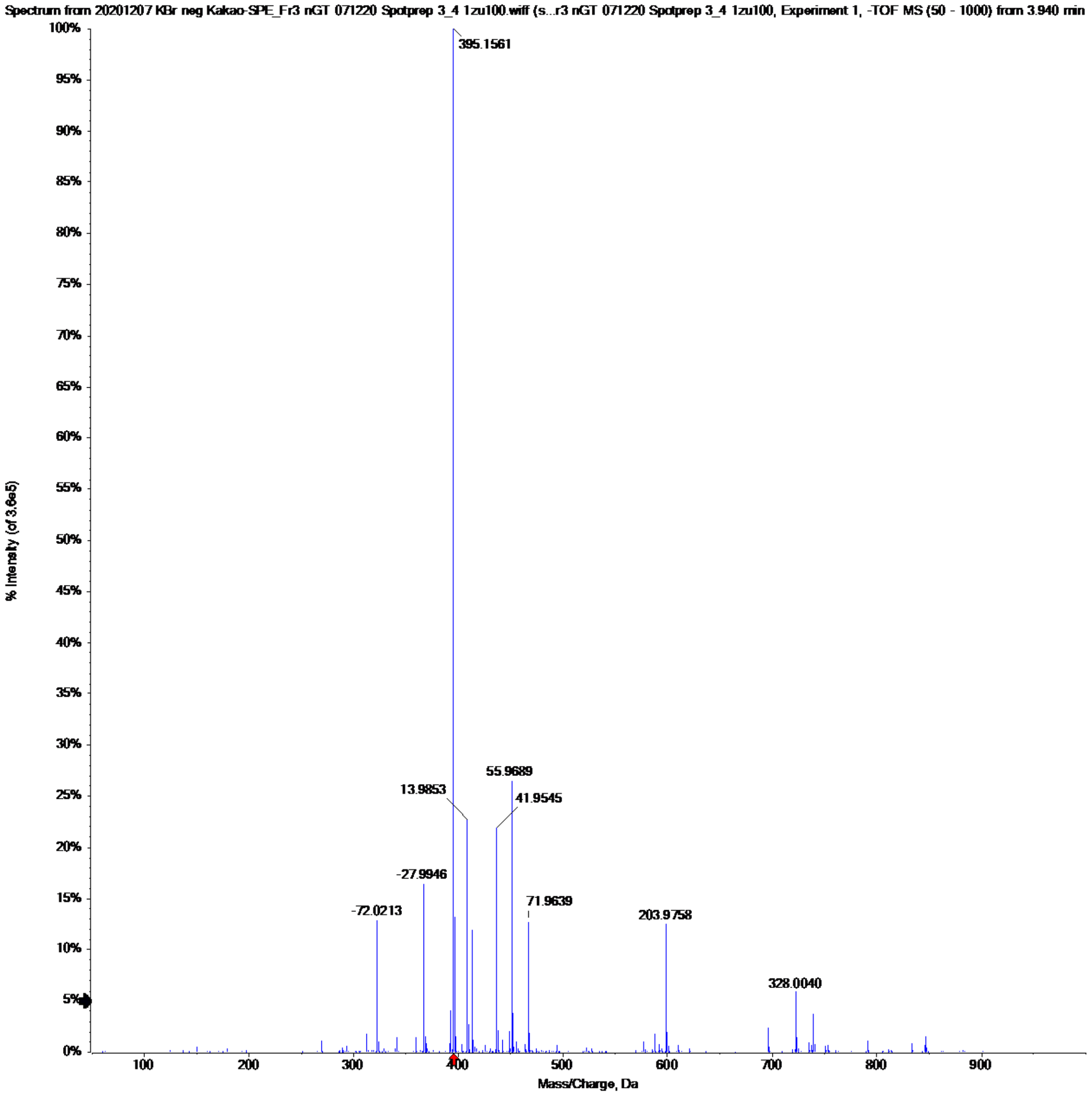

Figure S42: qToF-MS spectrum (survey scan) of second isomer (3.9 min), measured in SPE/ HPLC enriched acetone/water-extract of raw cocoa beans.

HOJA sulfat

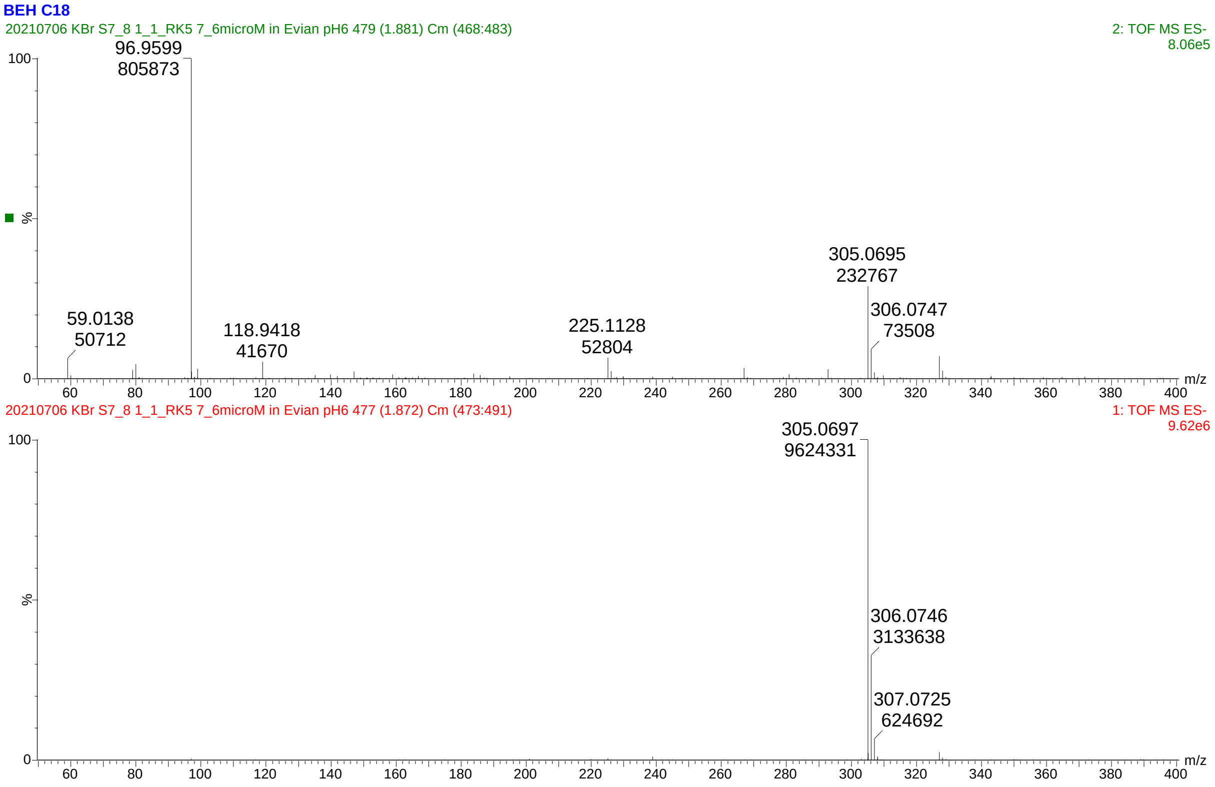

Figure S43: ToF-MS^E spectra of HOJA sulfate standard solution with high CE (top) and low CE (bottom).

NP007735: HMG gluc D

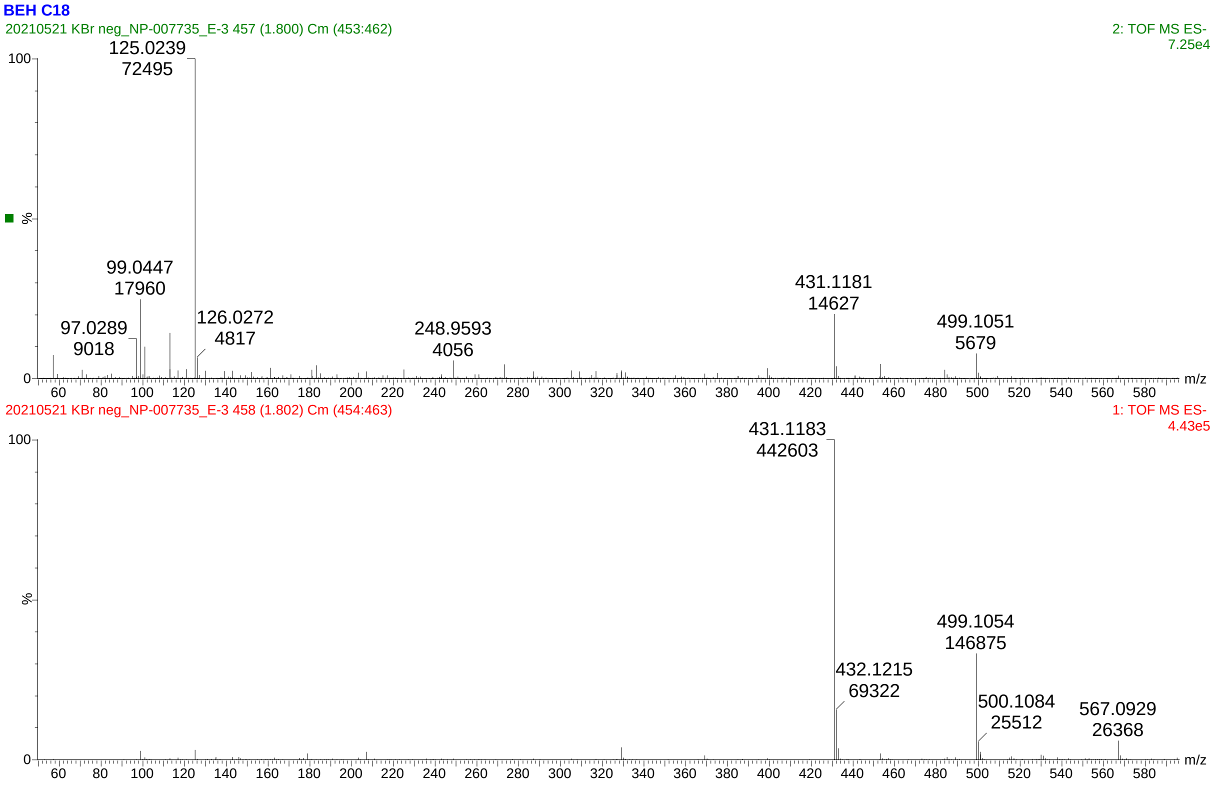

Figure S44: ToF-MS^E spectra of HMG gluc D standard solution with high CE (top) and low CE (bottom).

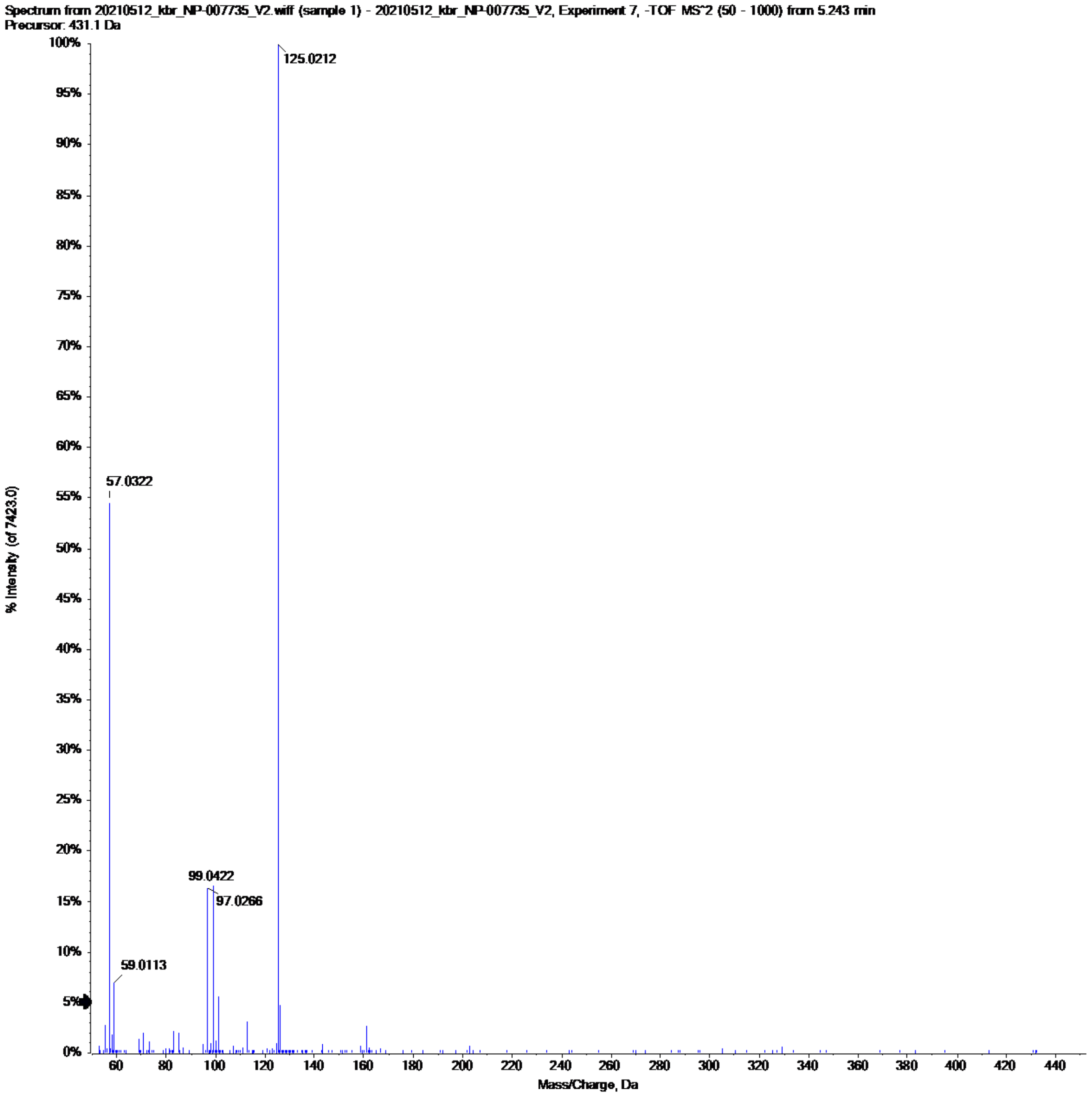

Figure S45: qToF-MS/MS spectrum of standard solution.

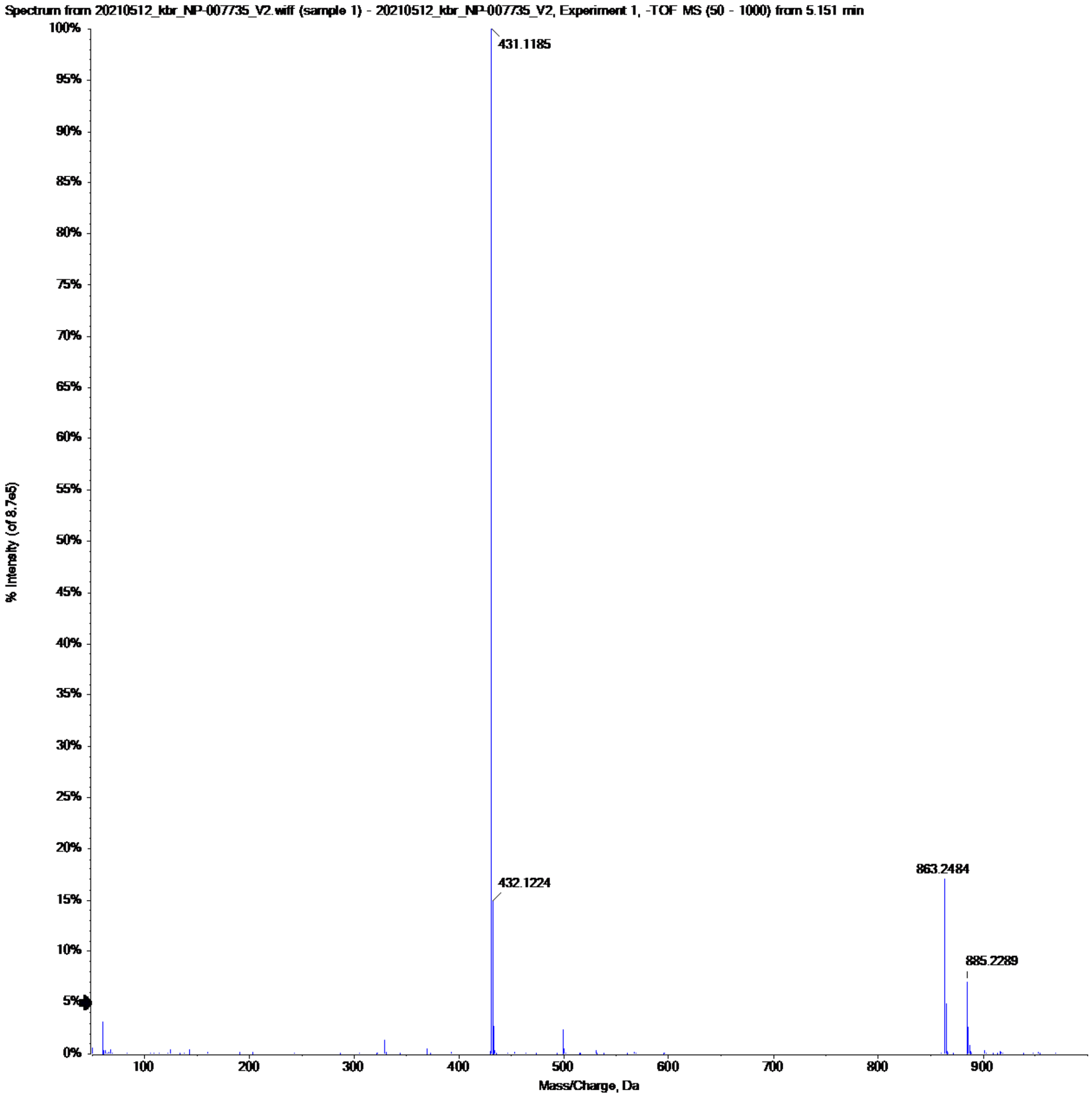

Figure S46: qToF-MS spectrum (survey scan) of standard solution.

NP014823: HMG gluc E

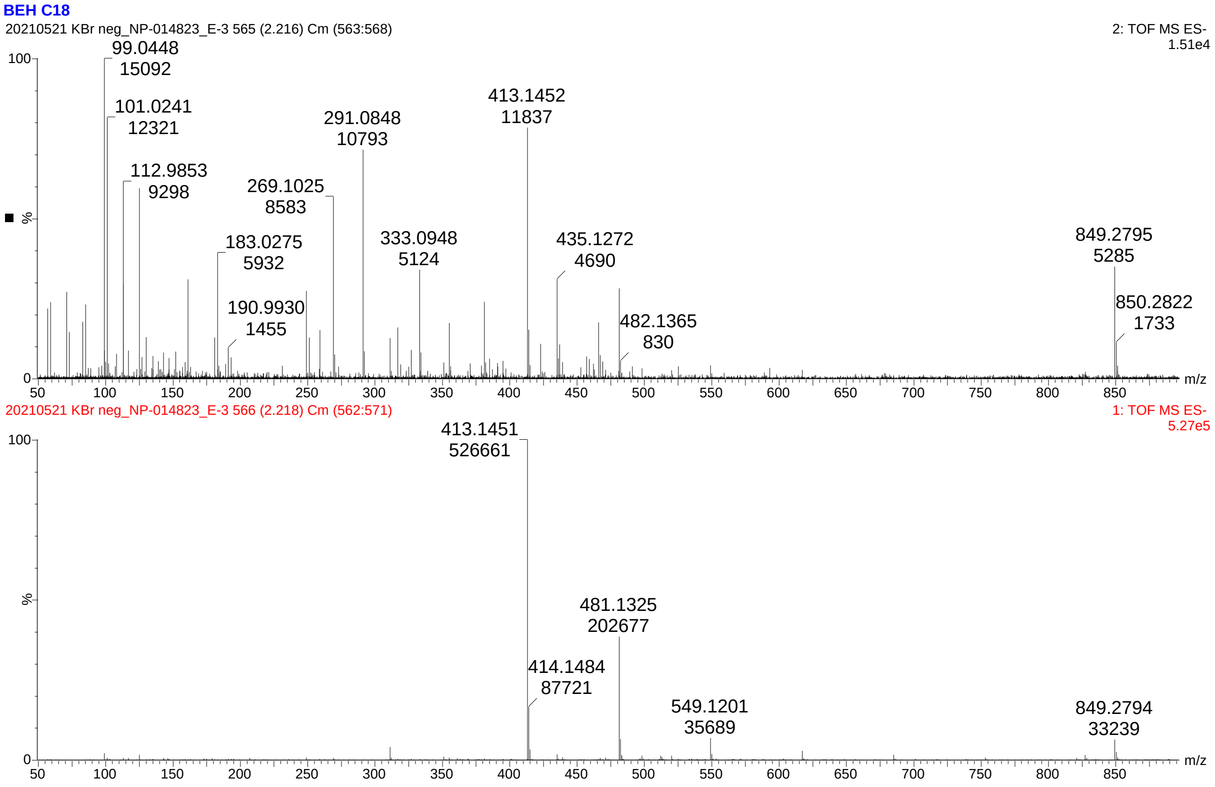

Figure S47: ToF-MS^E spectra of HMG gluc E standard solution with high CE (top) and low CE (bottom).

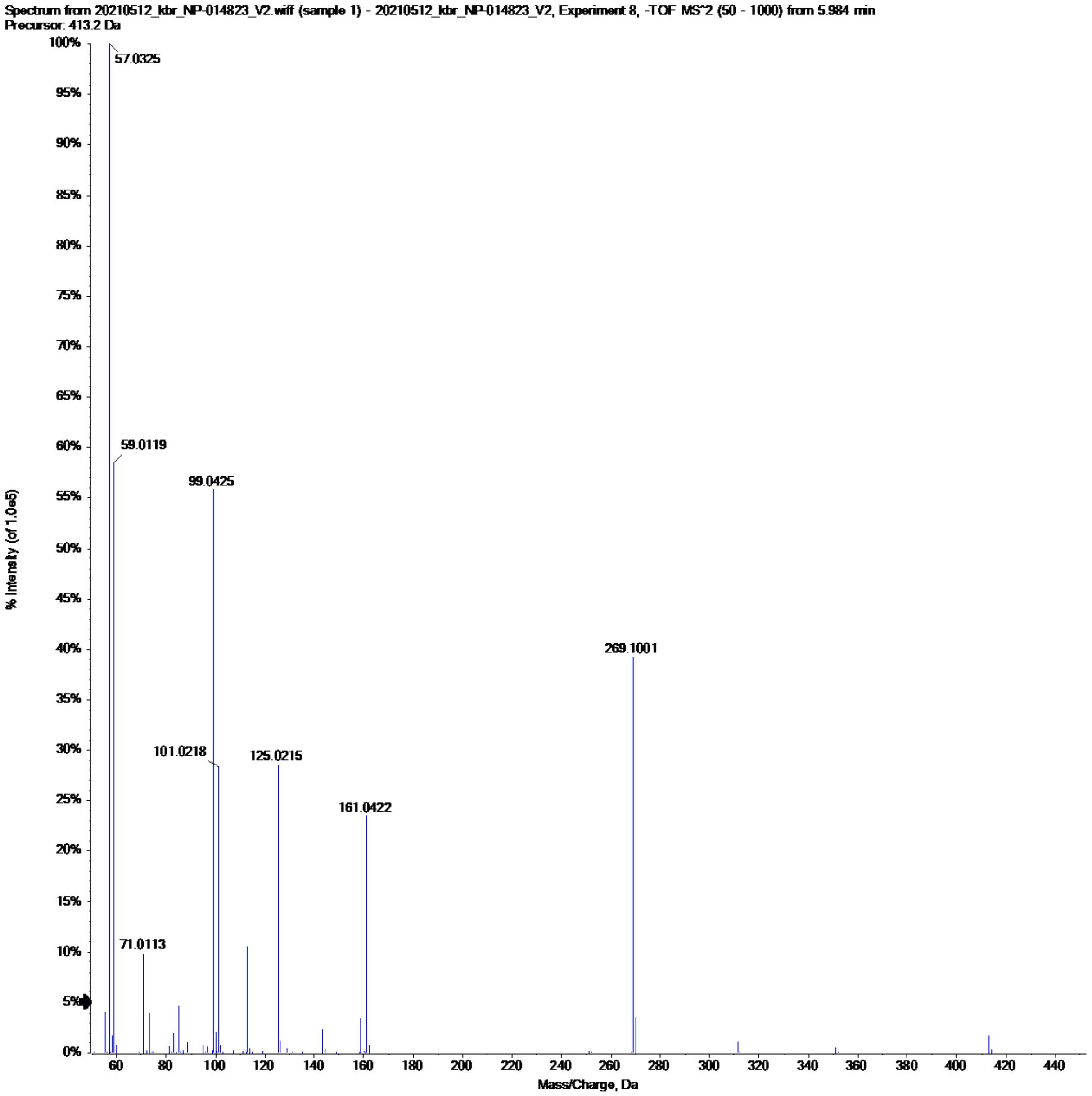

Figure S48: qToF-MS/MS spectrum of standard solution.

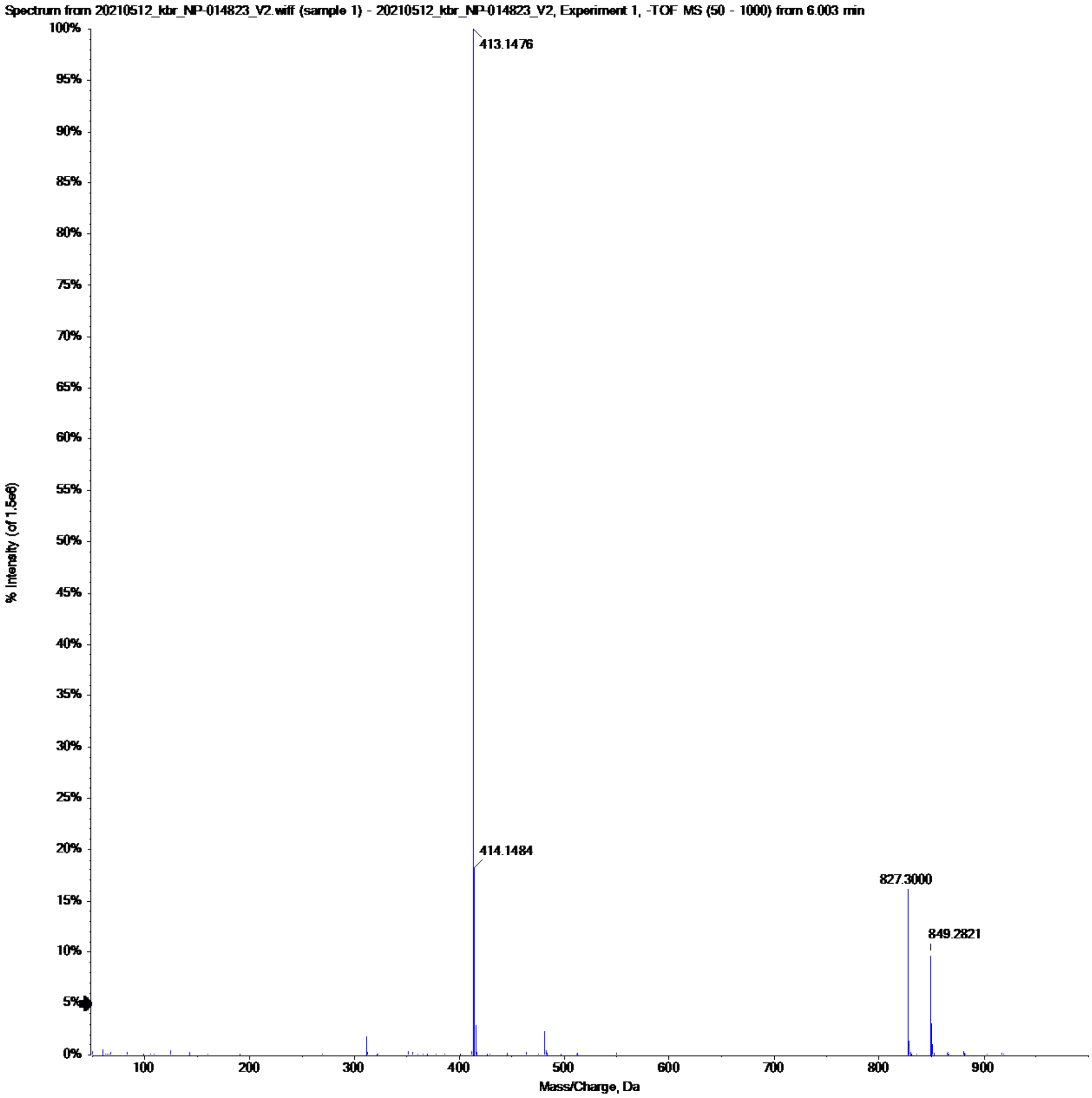

Figure S49: qToF-MS spectrum (survey scan) of standard solution.

NP015777: HMG gluc F

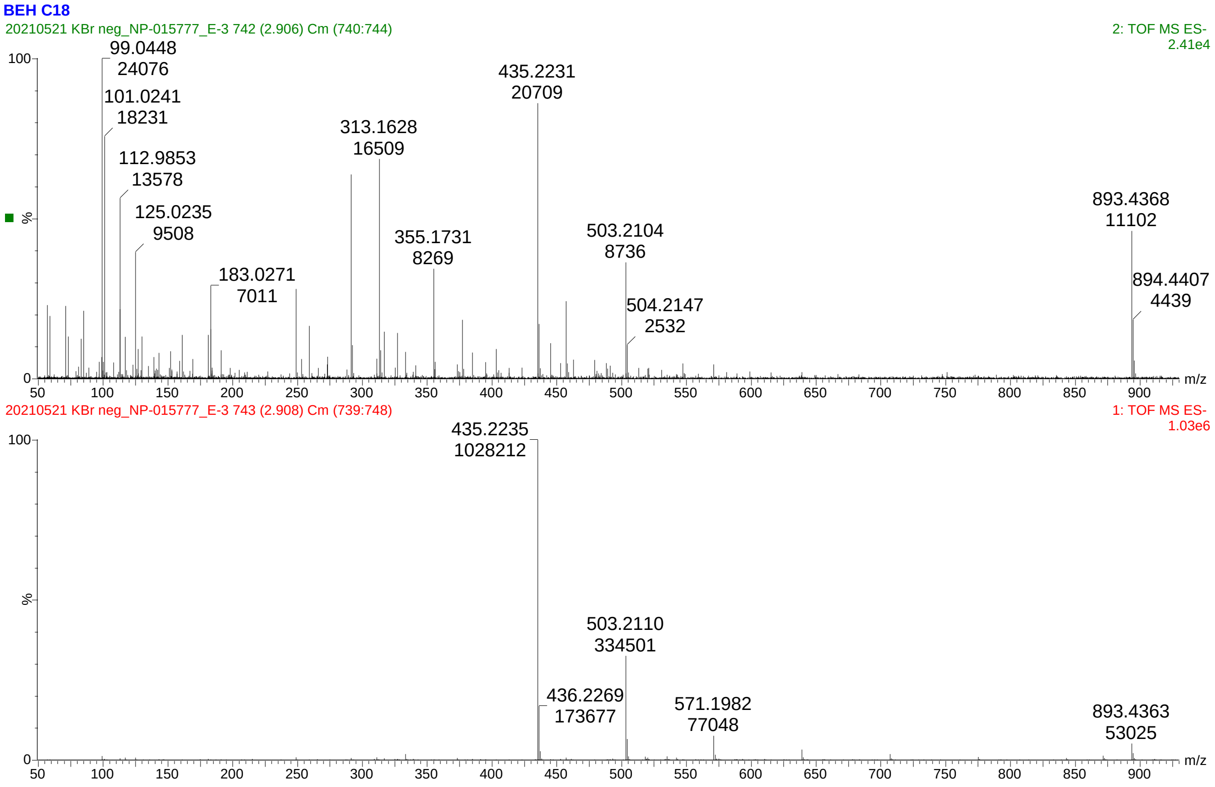

Figure S50: ToF-MS^E spectra of HMG gluc F standard solution with high CE (top) and low CE (bottom).

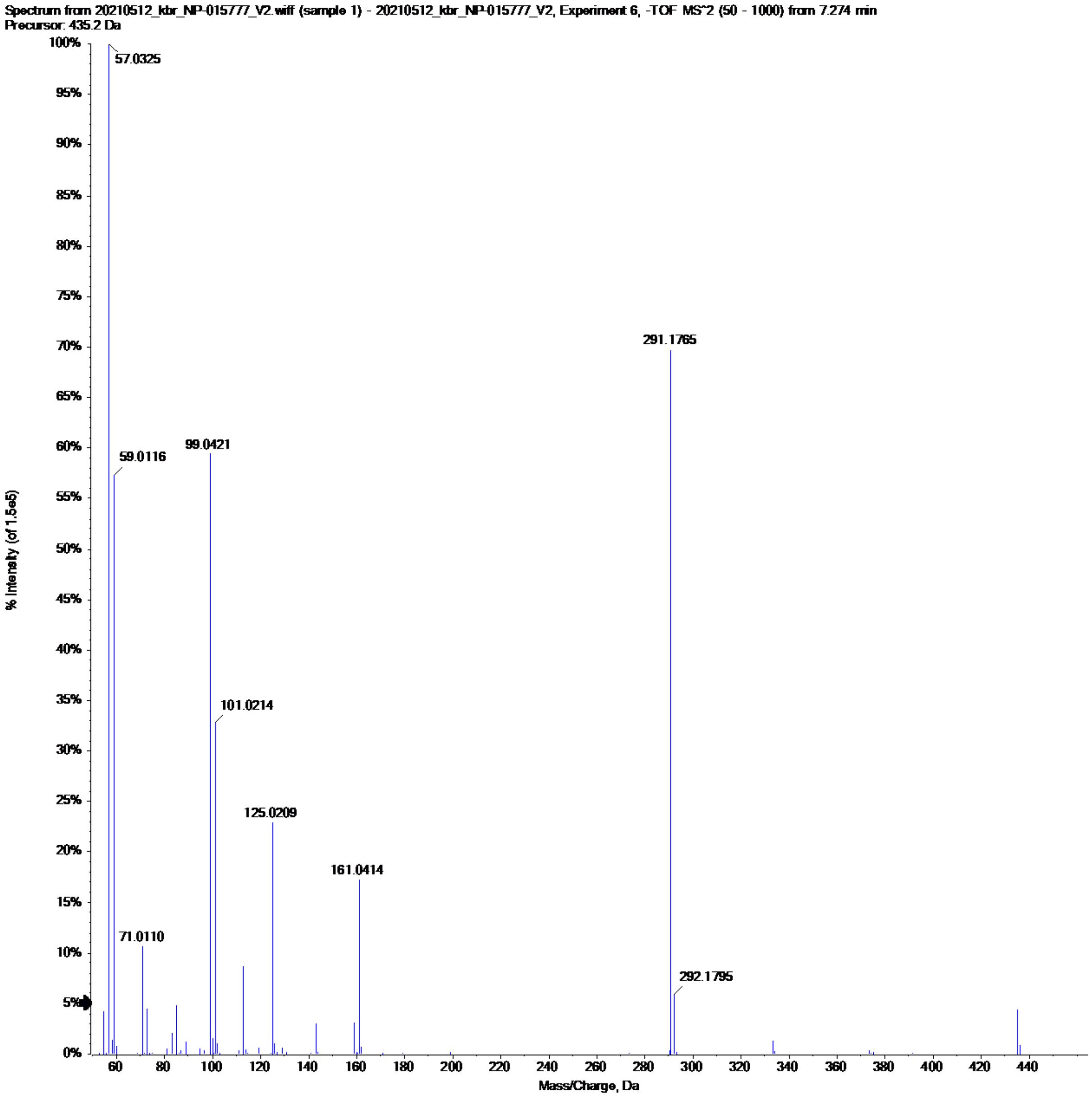

Figure S51: qToF-MS/MS spectrum of standard solution.

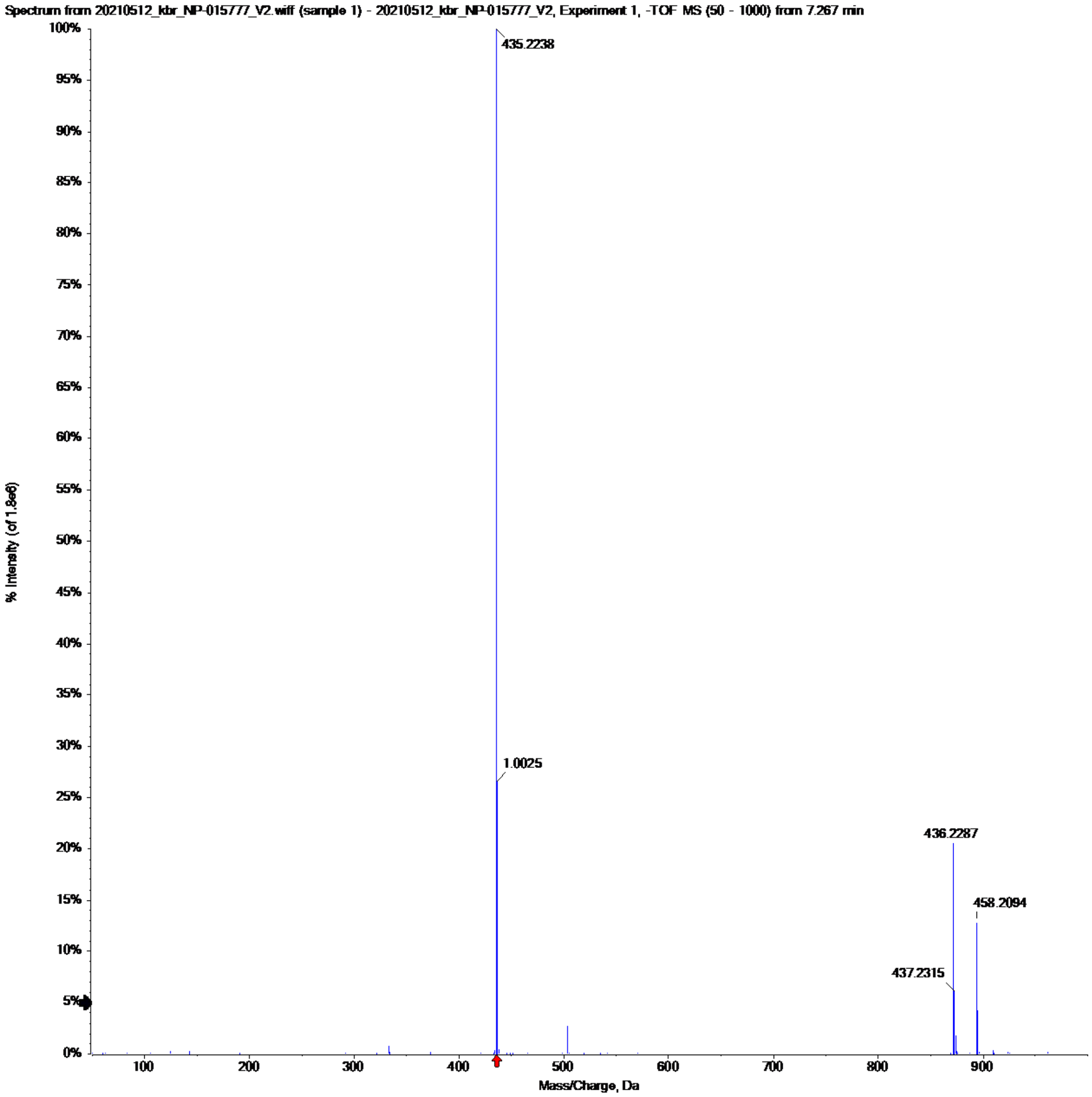

Figure S52: qToF-MS spectrum (survey scan) of standard solution.

NP018595: HMG gluc G

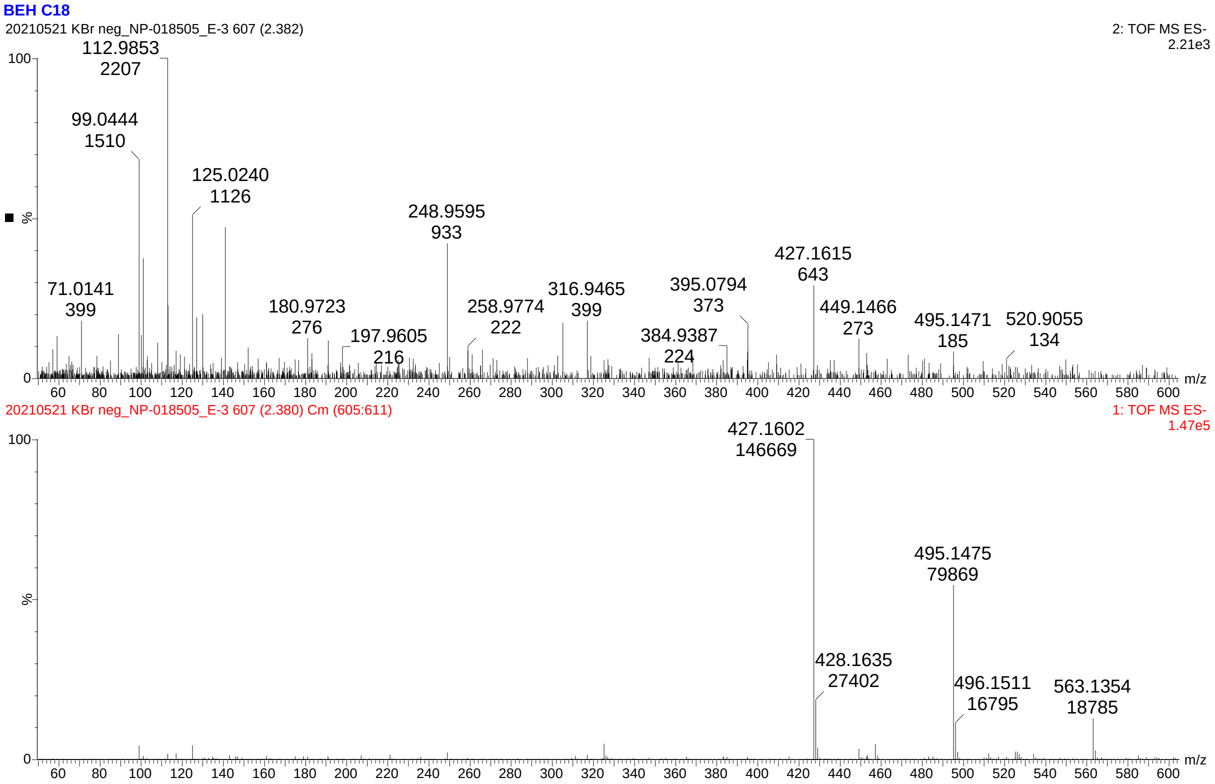

Figure S53: ToF-MS^E spectra of HMG gluc G standard solution with high CE (top) and low CE (bottom).

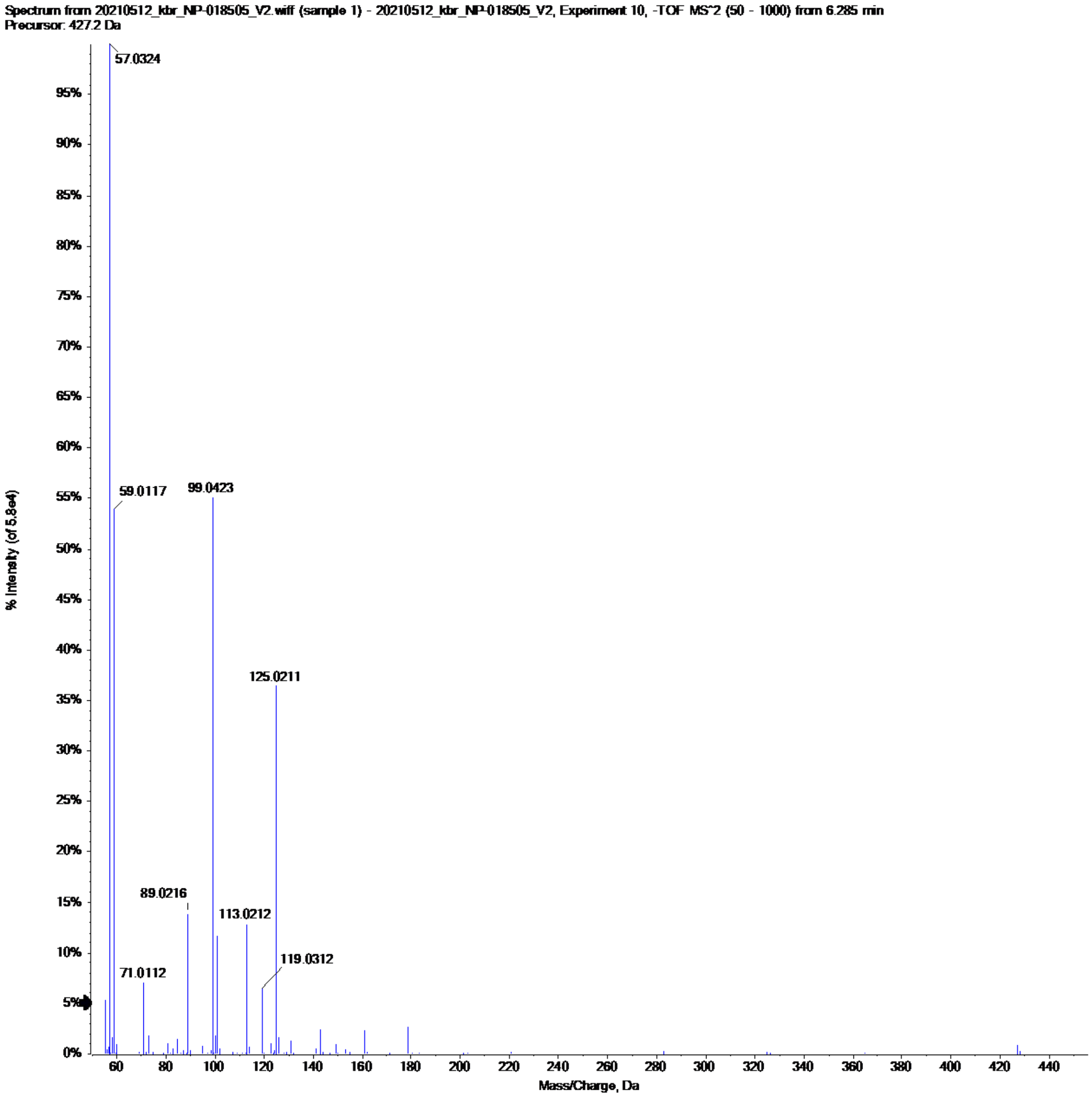

Figure S54: qToF-MS/MS spectrum of standard solution.

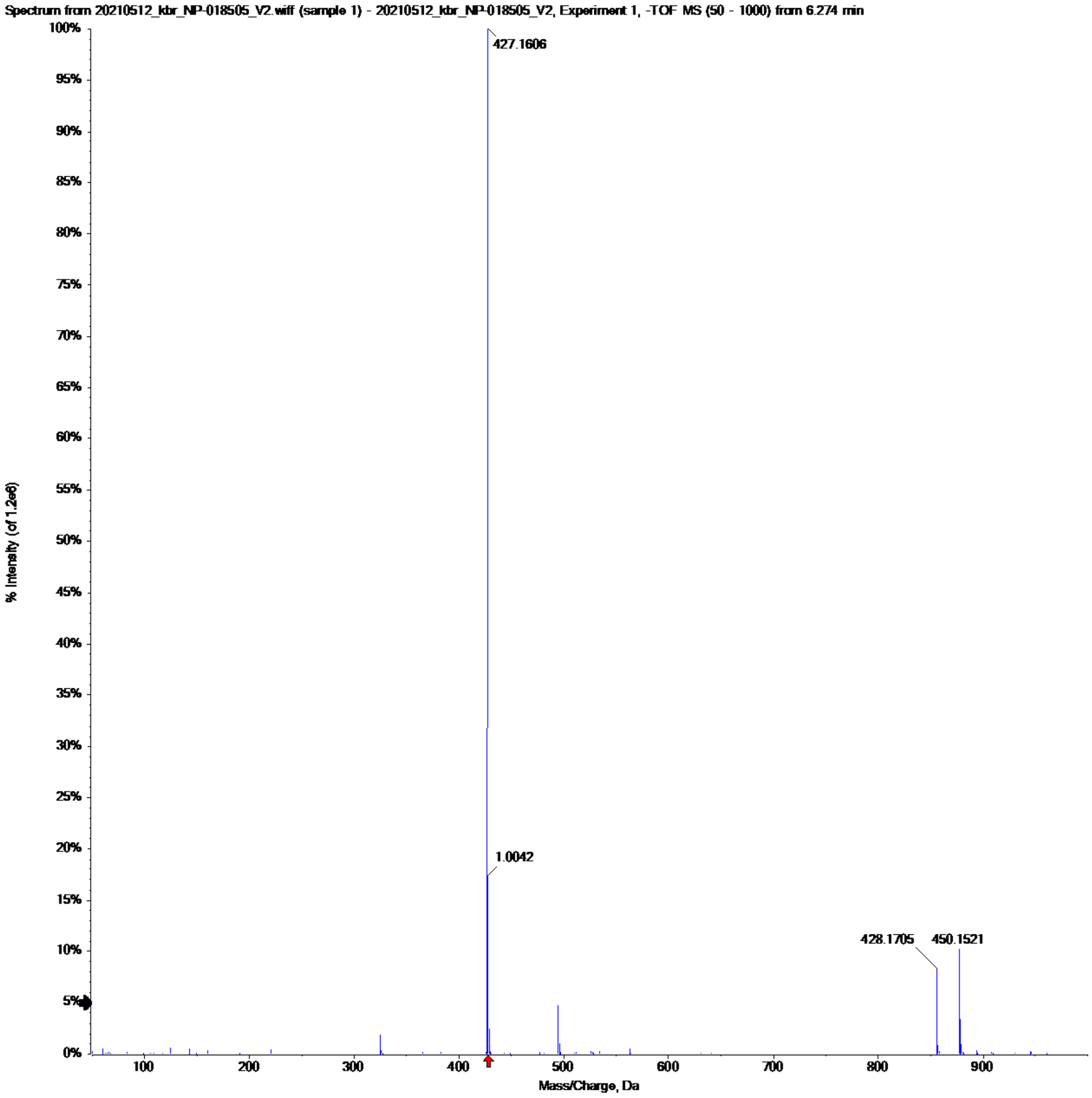

Figure S55: qToF-MS spectrum (survey scan) of standard solution.

NP021228: HMG gluc H

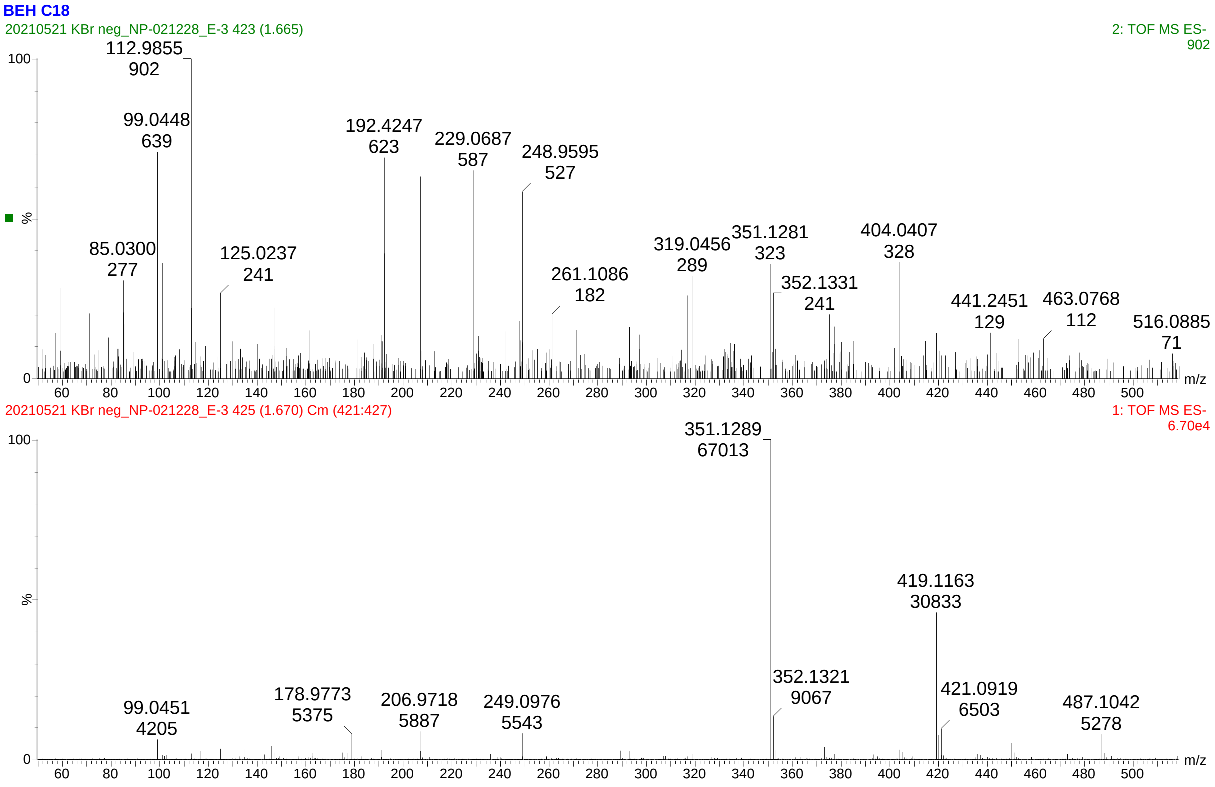

Figure S56: ToF-MS^{E} spectra of HMG gluc H standard solution with high CE (top) and low CE (bottom).

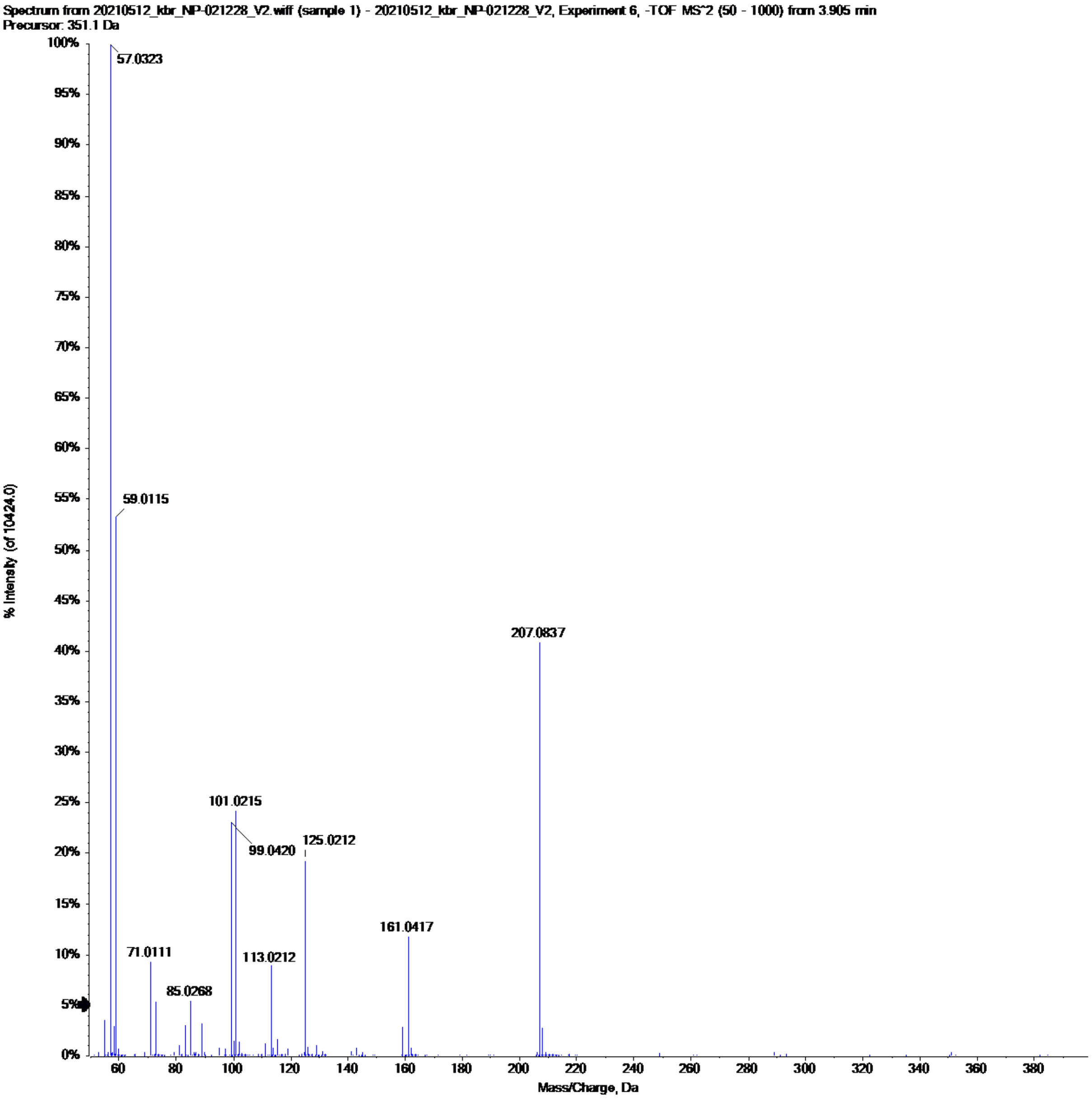

Figure S57: qToF-MS/MS spectrum of standard solution.

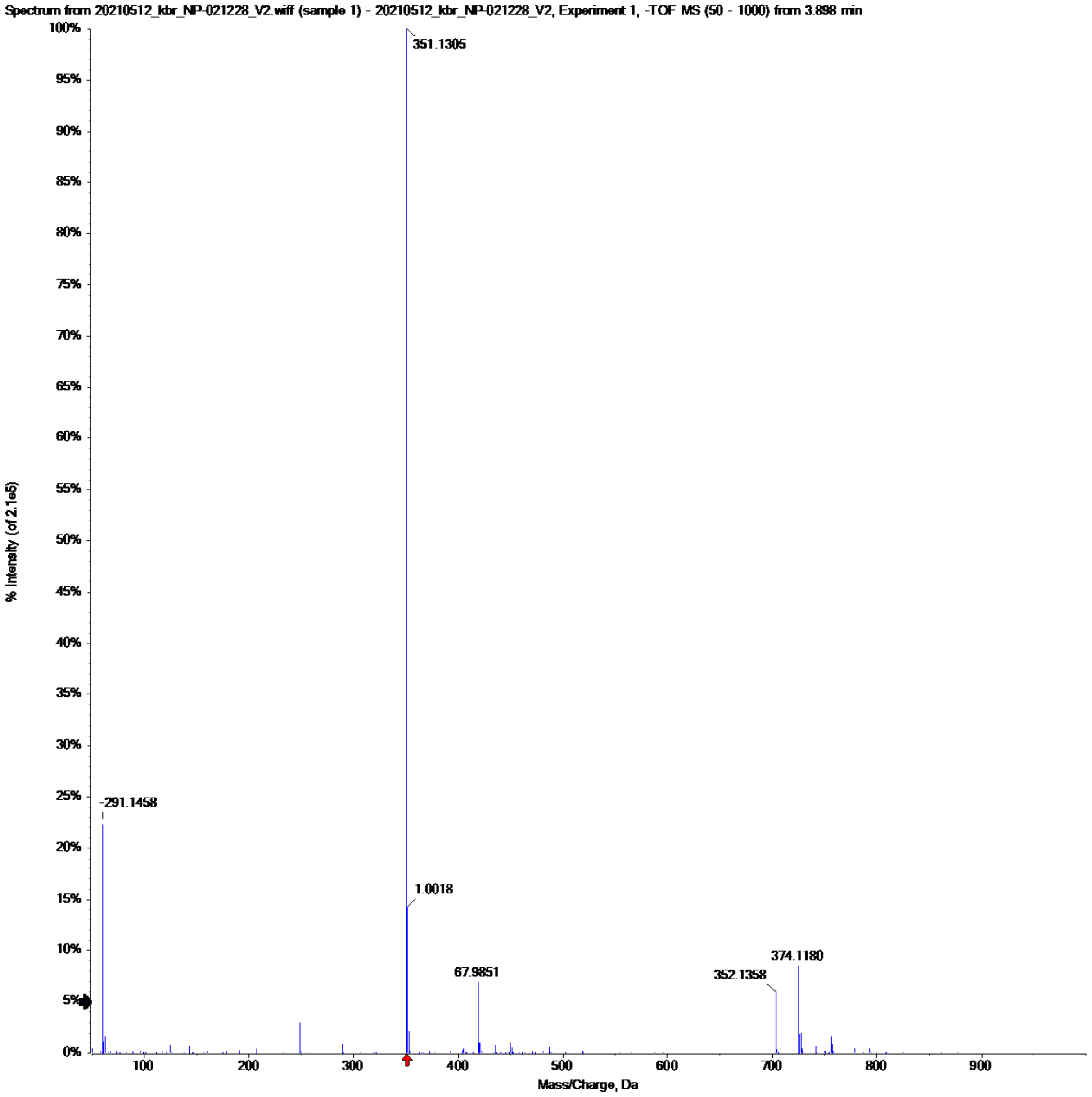

Figure S58: qToF-MS spectrum (survey scan) of standard solution.

NP022515: HMG gluc I

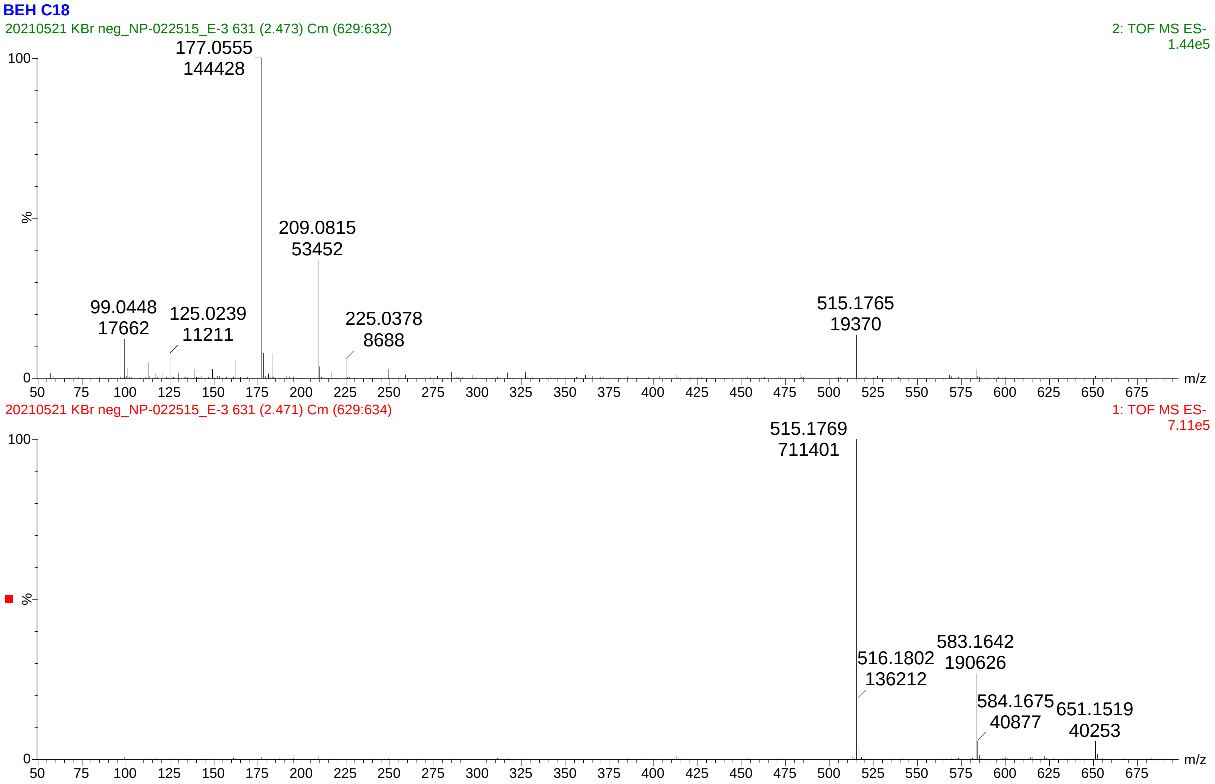

Figure S59: ToF-MS^E spectra of HMG gluc I standard solution with high CE (top) and low CE (bottom).

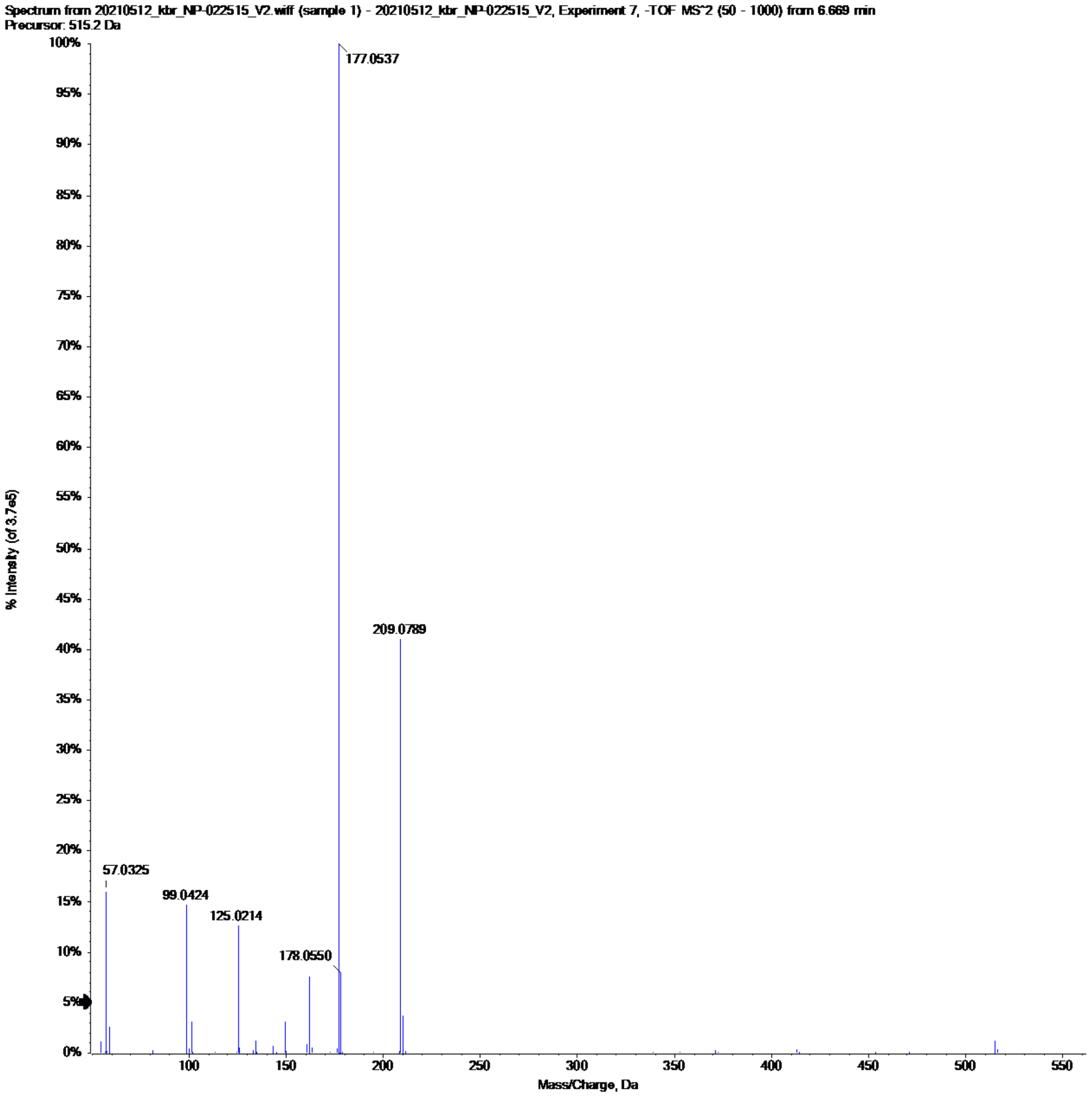

Figure S60: qToF-MS/MS spectrum of standard solution.

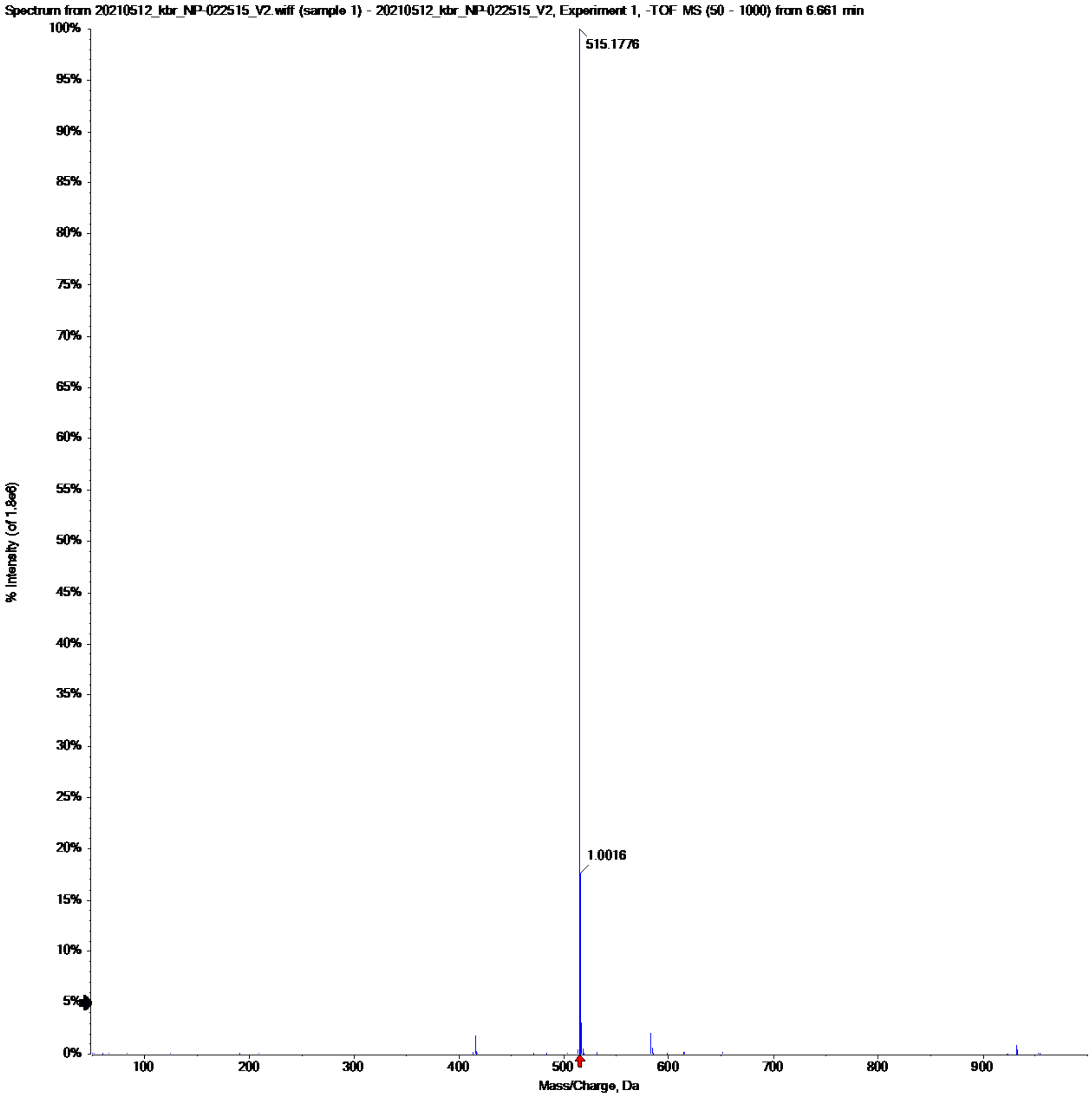

Figure S61: qToF-MS spectrum (survey scan) of standard solution.

NP022828: HMG gluc J

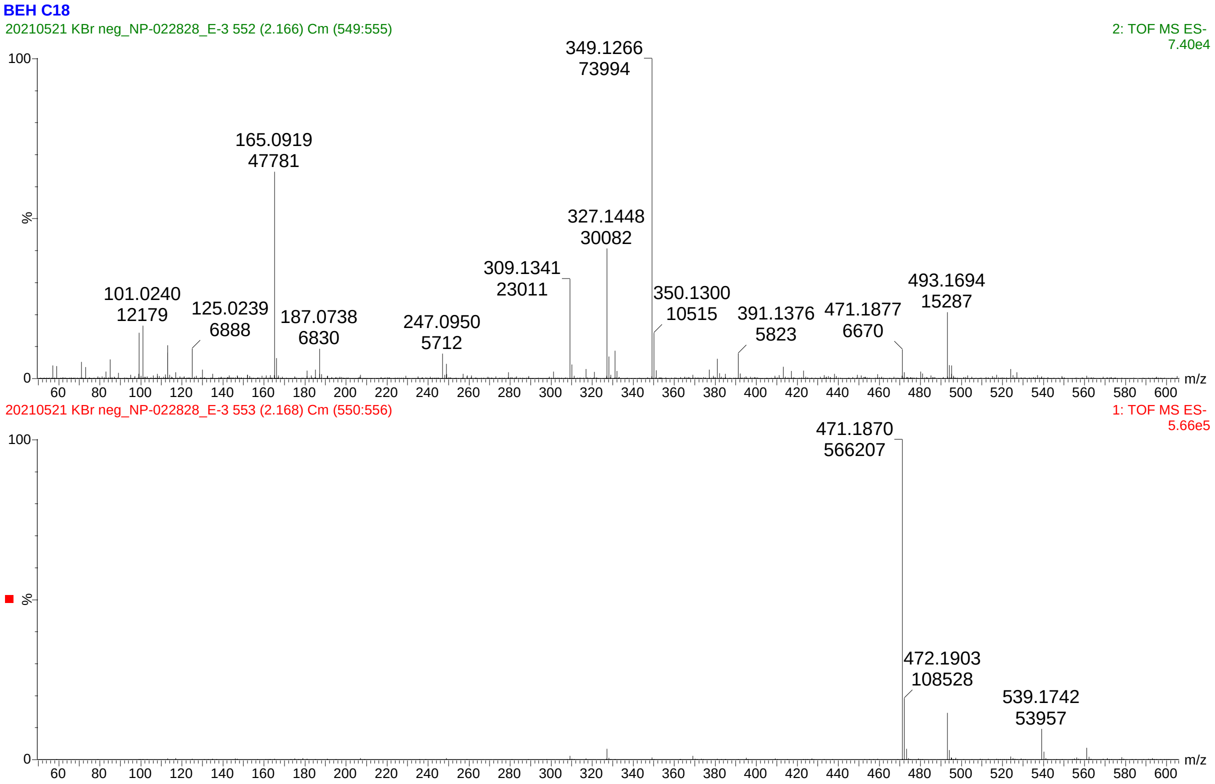

Figure S62: ToF-MS^E spectra of HMG gluc J standard solution with high CE (top) and low CE (bottom).

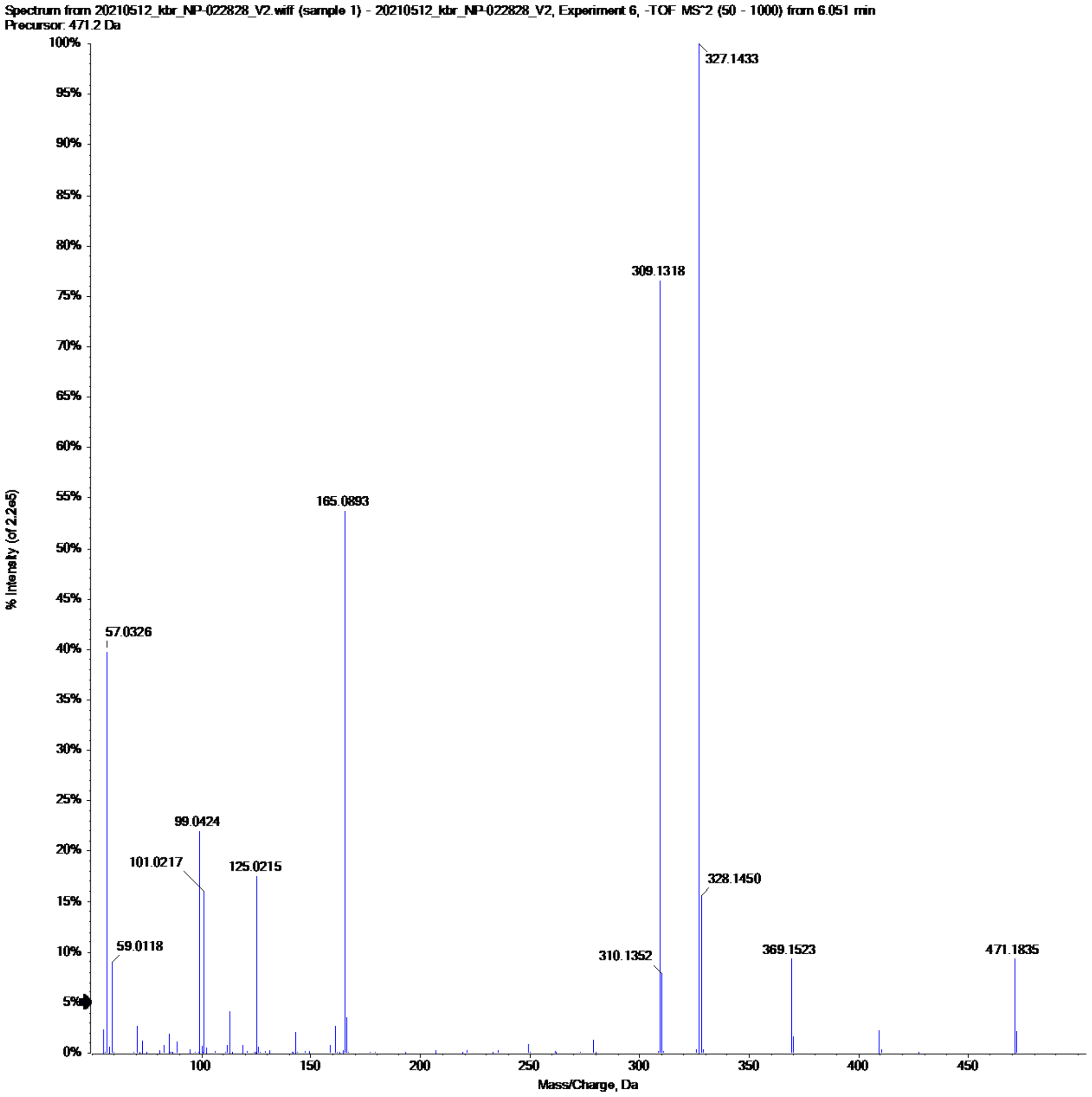

Figure S63: qToF-MS/MS spectrum of standard solution.

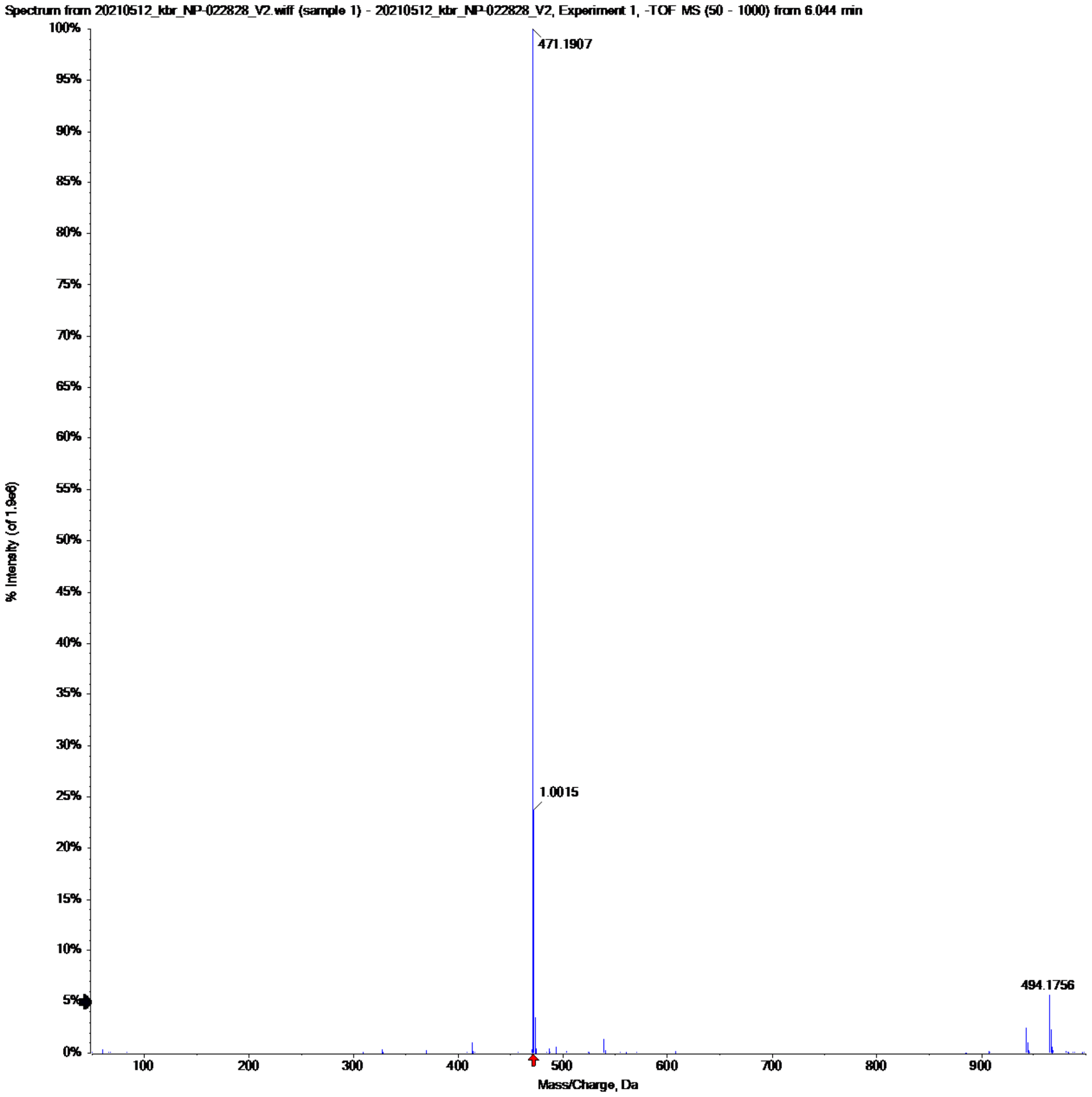

Figure S64: qToF-MS spectrum (survey scan) of standard solution.

NP023425: HMG gluc K

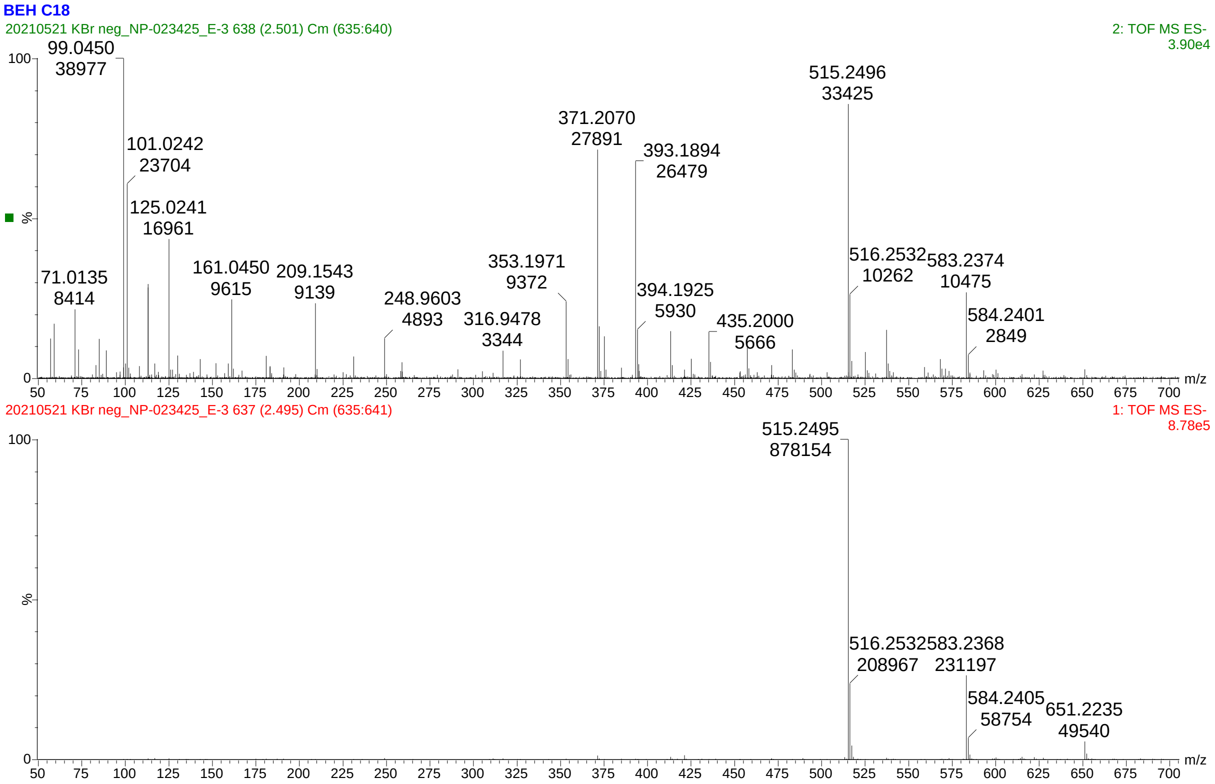

Figure S65: ToF-MS^E spectra of HMG gluc K standard solution with high CE (top) and low CE (bottom).

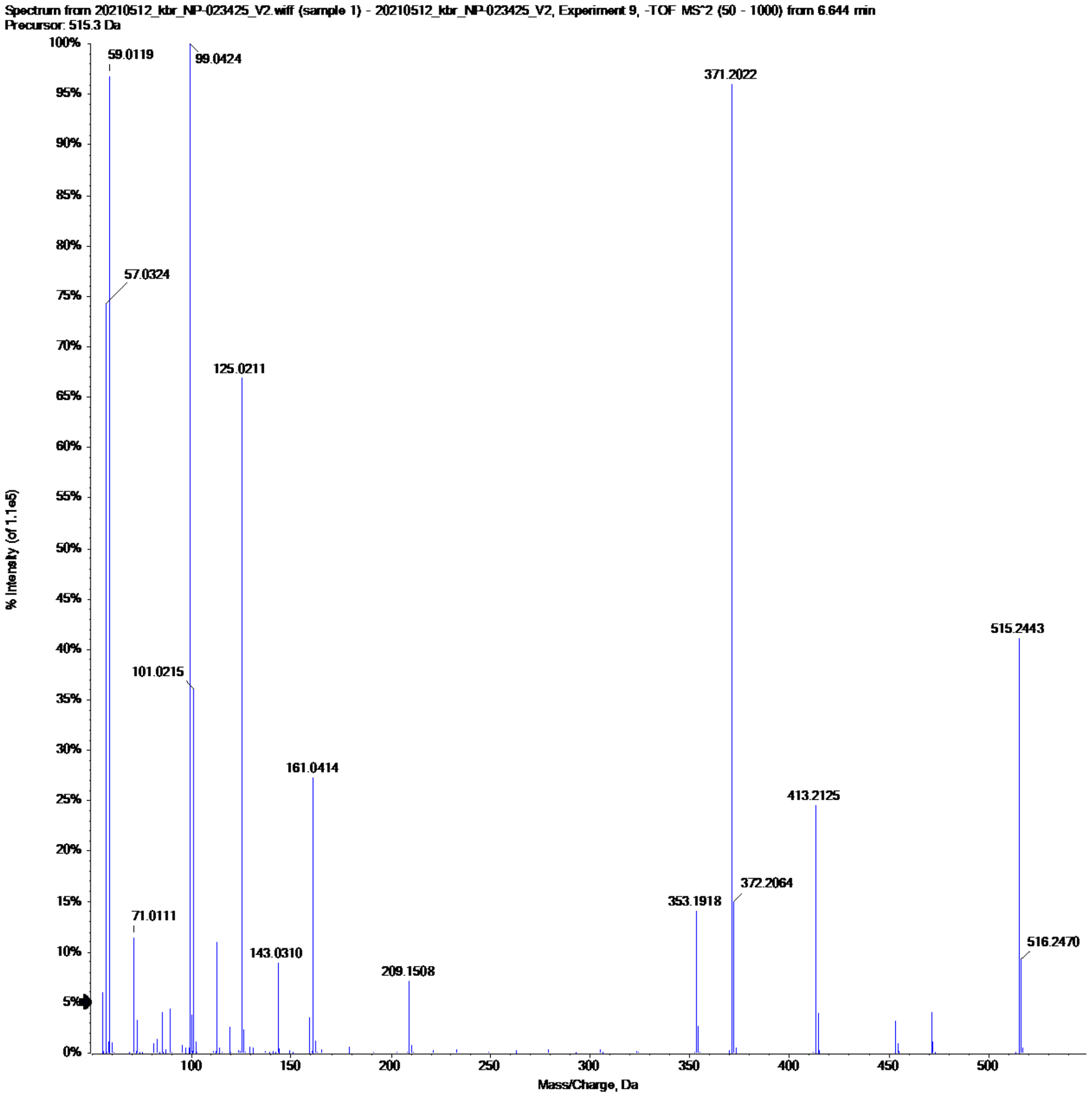

Figure S66: qToF-MS/MS spectrum of standard solution.

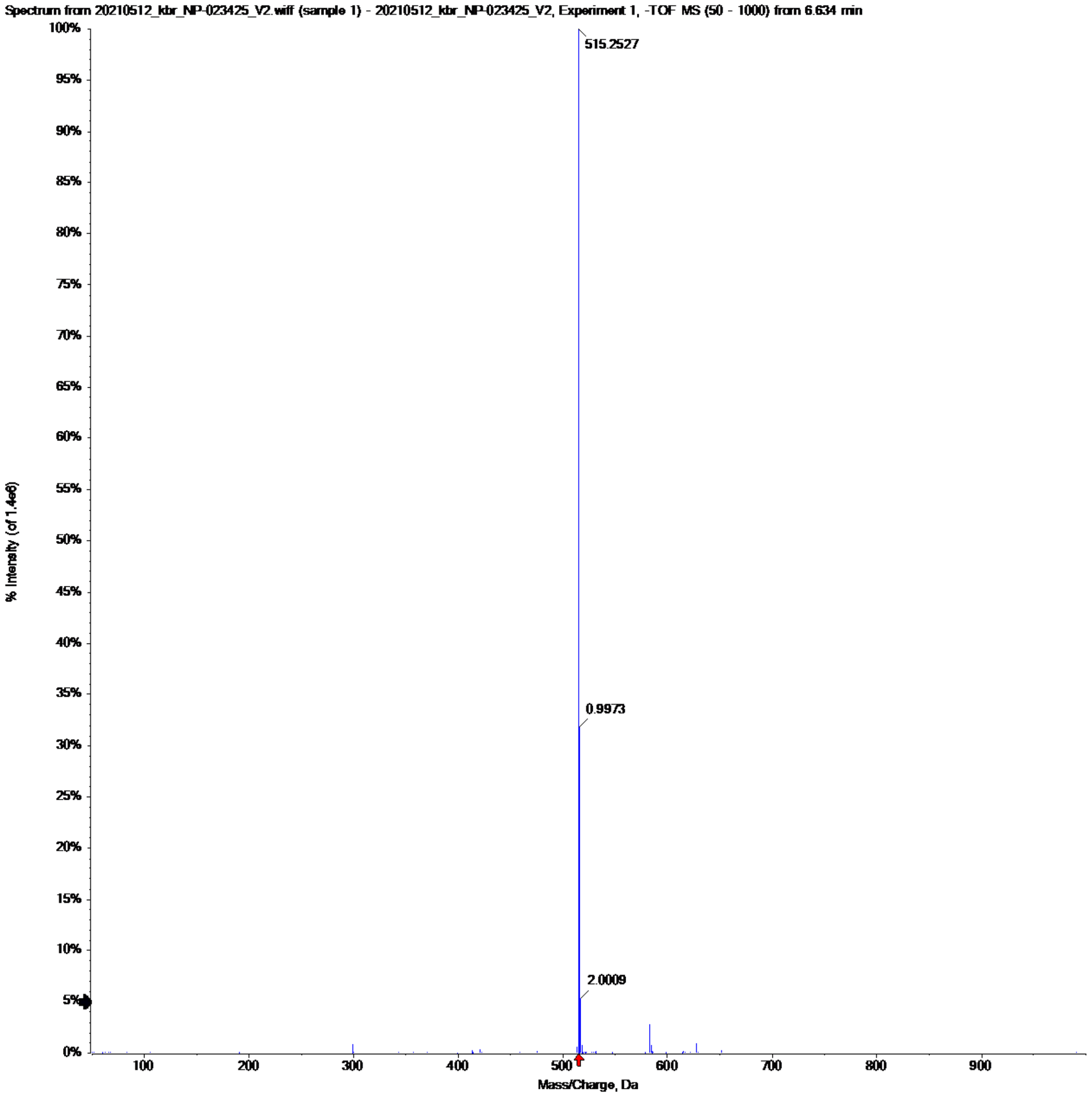

Figure S67: qToF-MS spectrum (survey scan) of standard solution.

NP023820: HMG gluc L

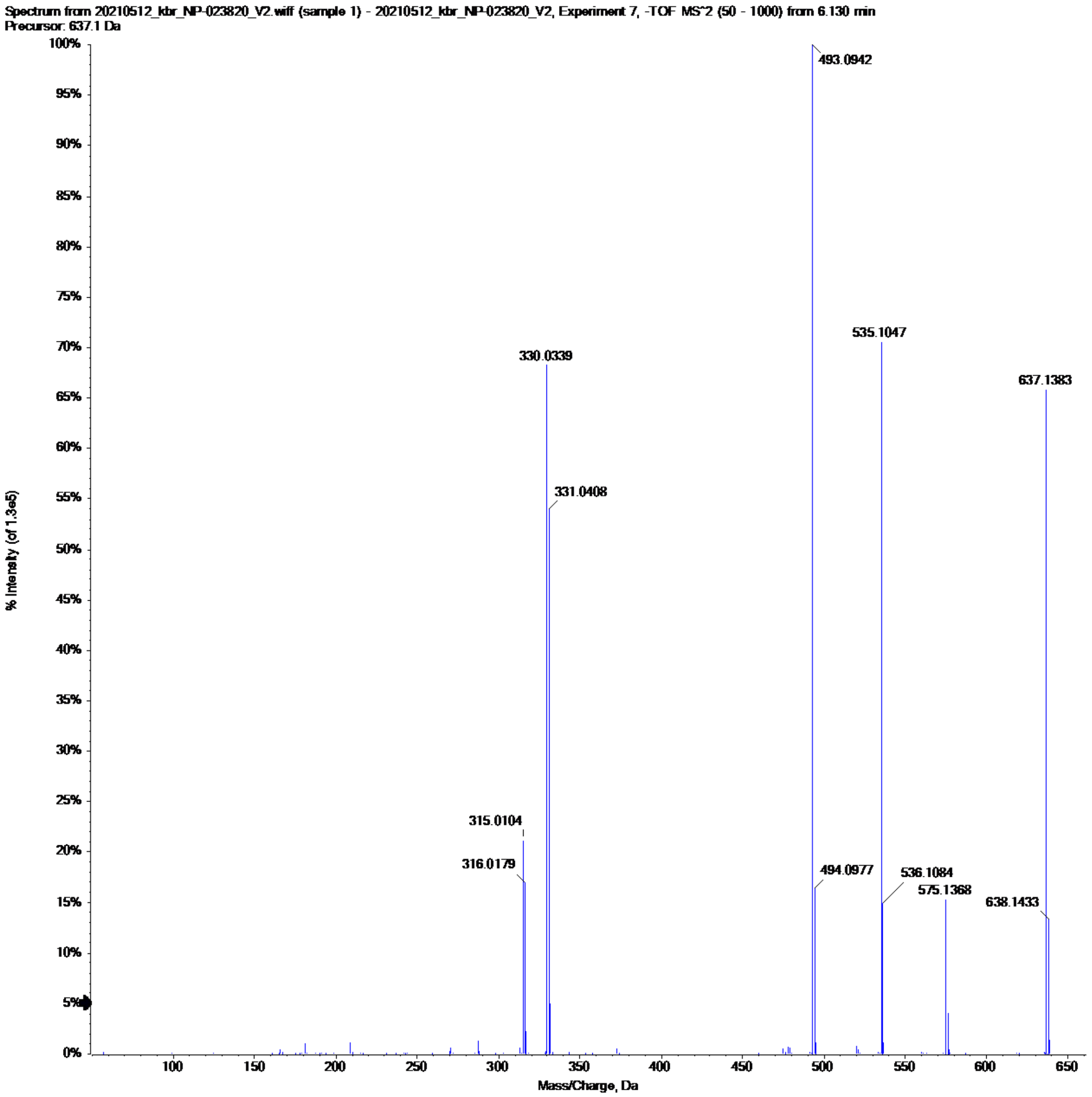

Figure S68: qToF-MS/MS spectrum of standard solution.

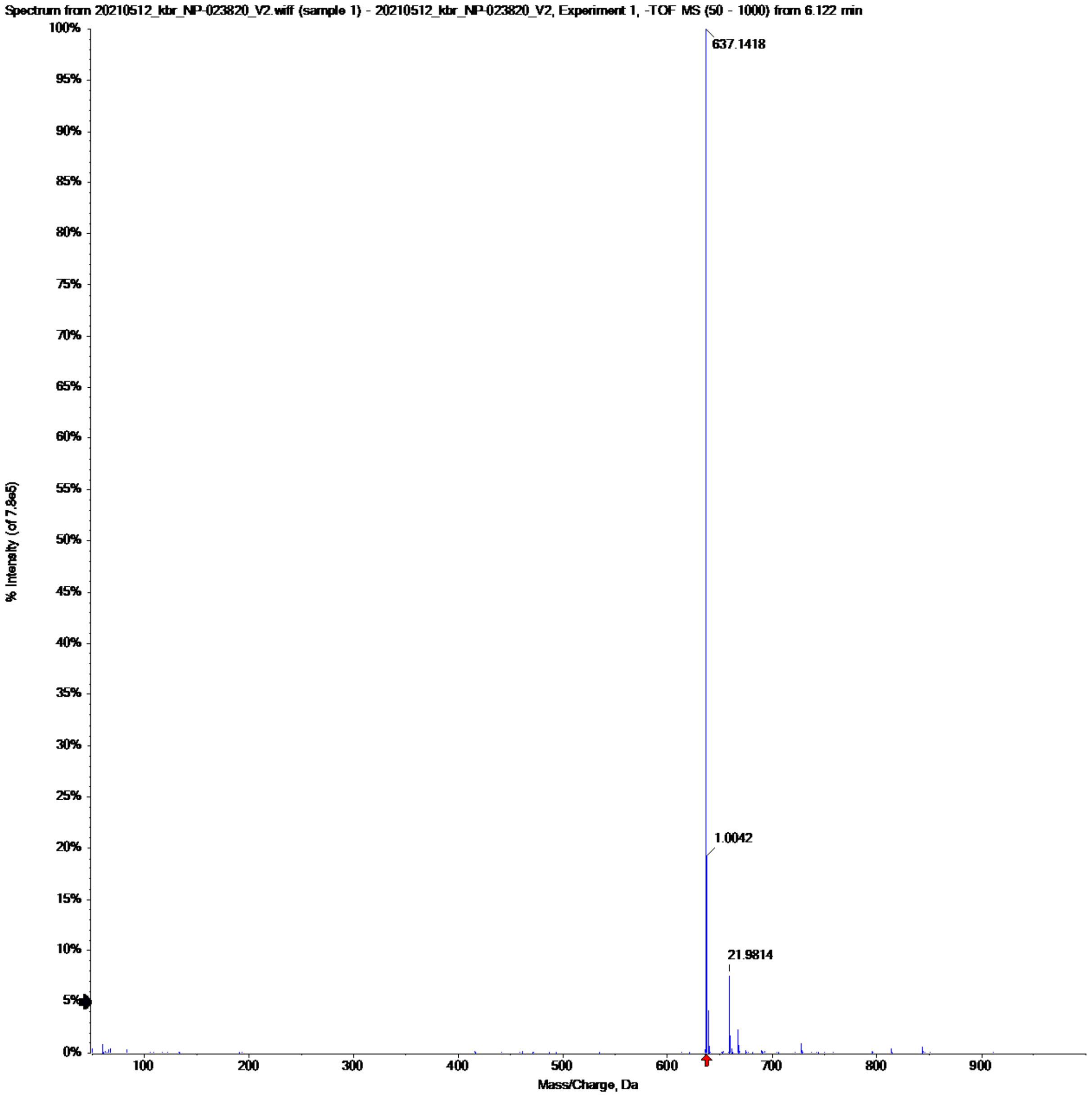

Figure S69: qToF-MS spectrum (survey scan) of standard solution.

NP023821: HMG gluc M

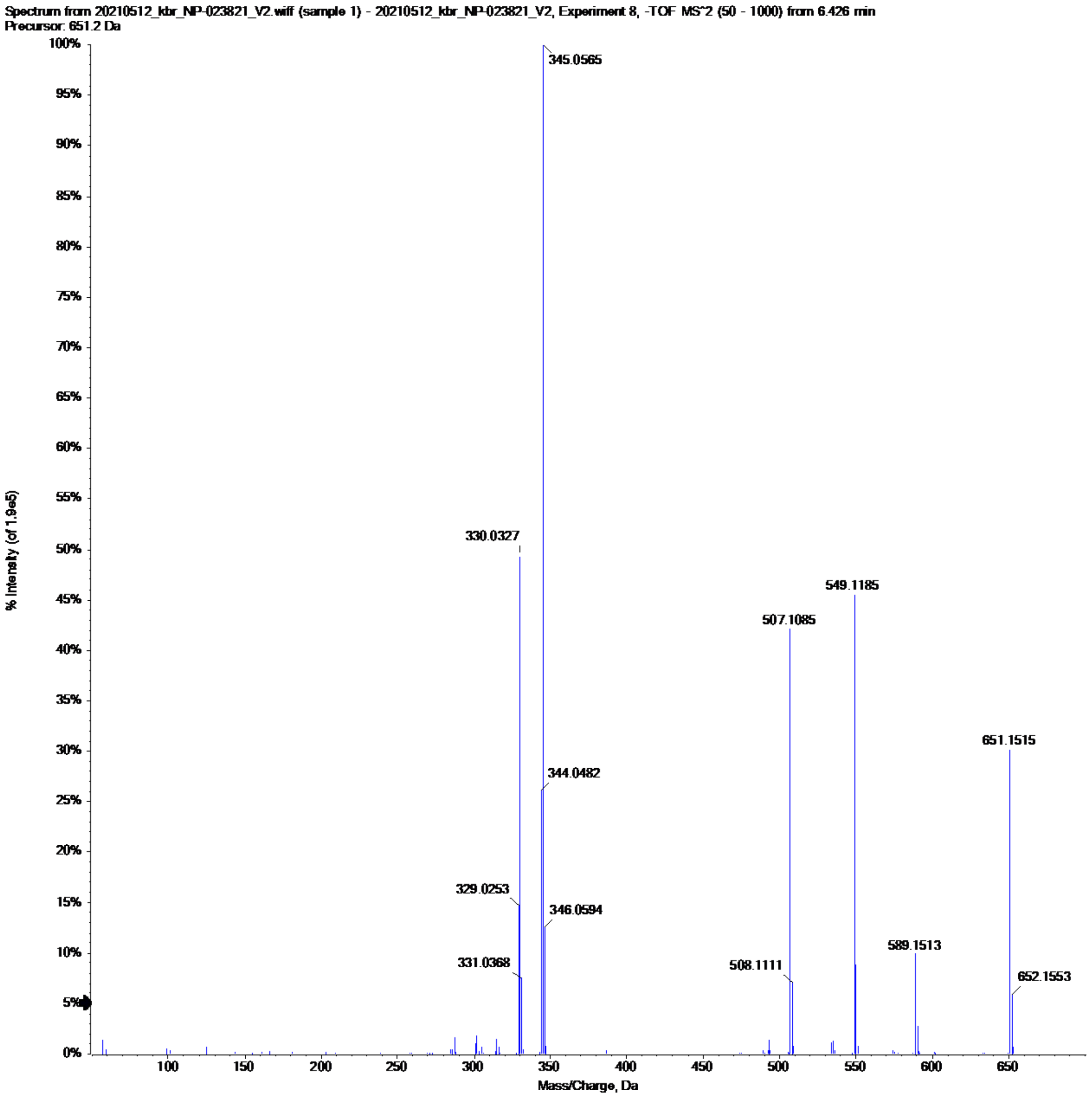

Figure S70: qToF-MS/MS spectrum of standard solution.

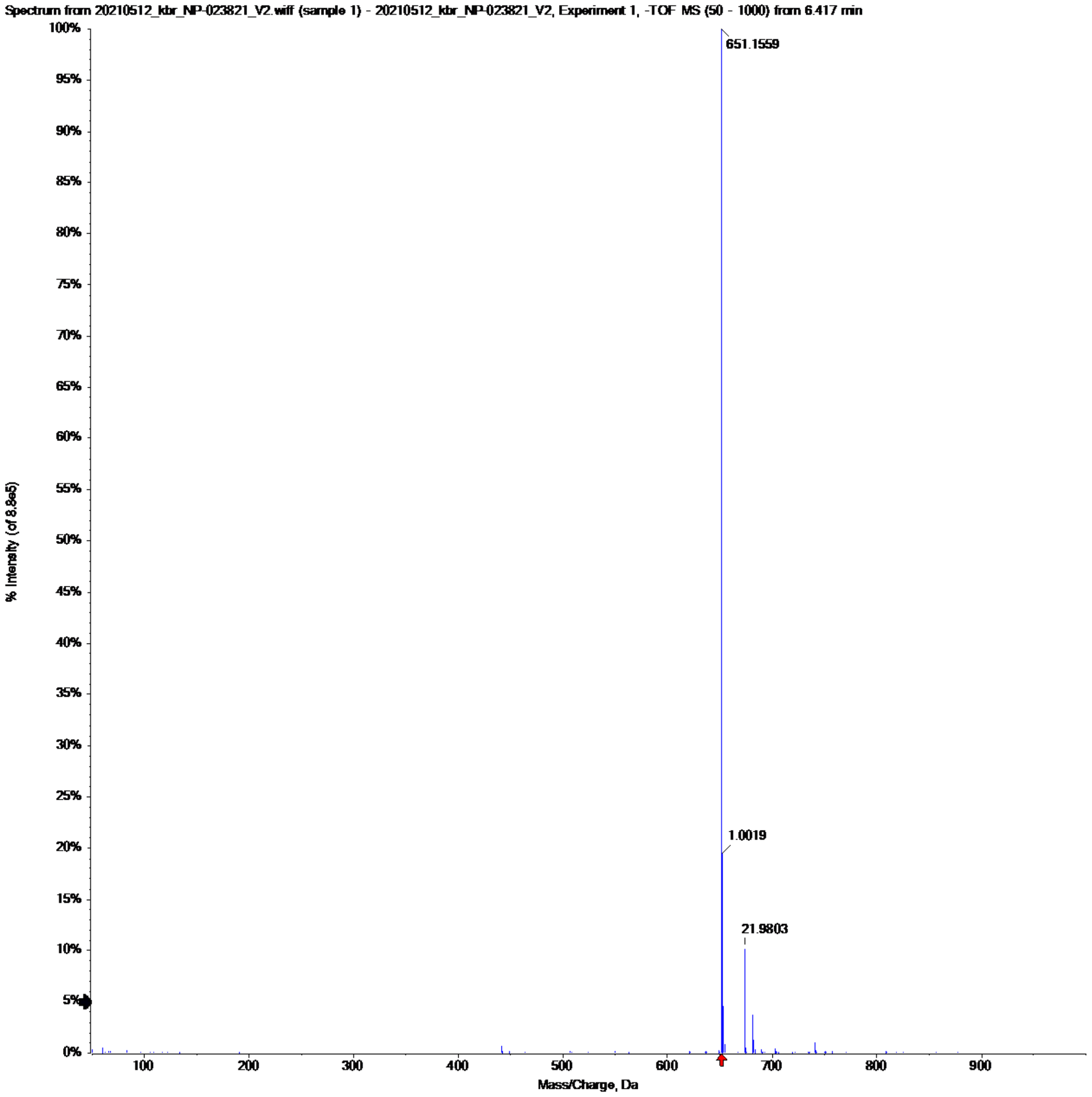

Figure S71: qToF-MS spectrum (survey scan) of standard solution.

NP024048: HMG gluc N

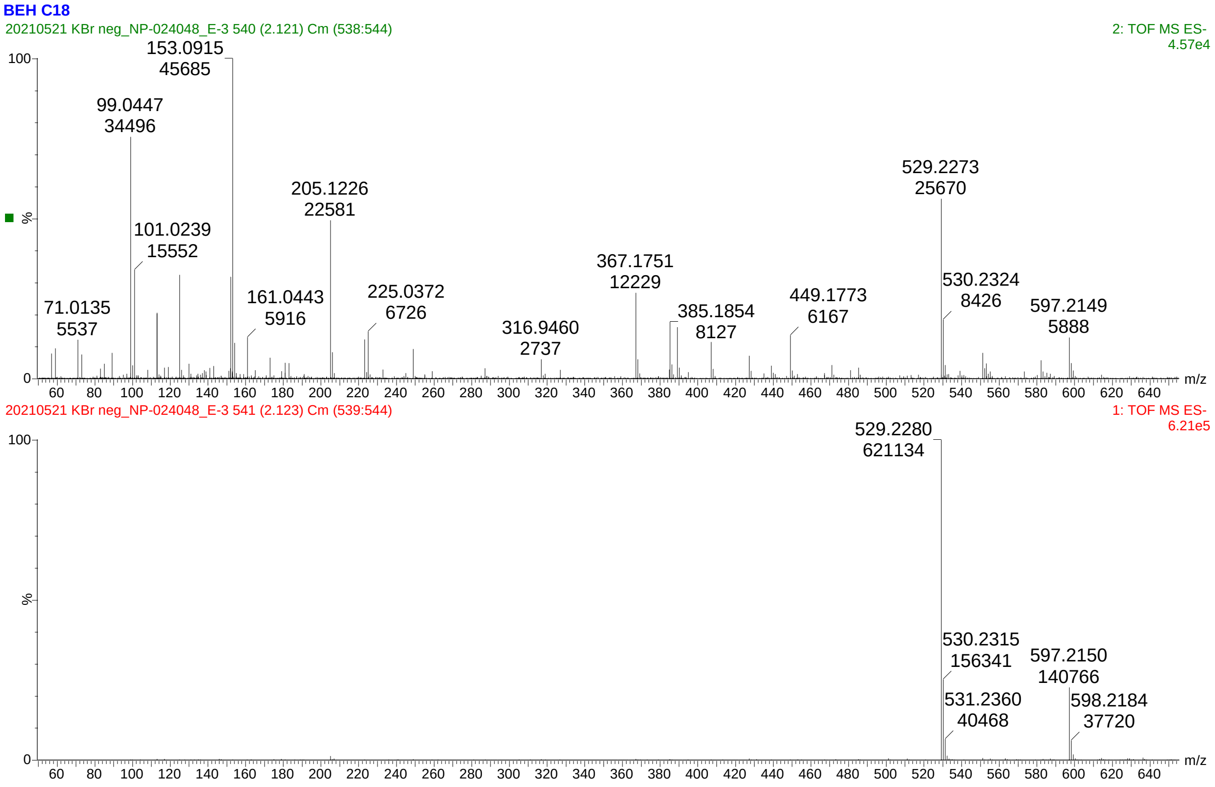

Figure S72: ToF-MS^E spectra of HMG gluc N standard solution with high CE (top) and low CE (bottom).

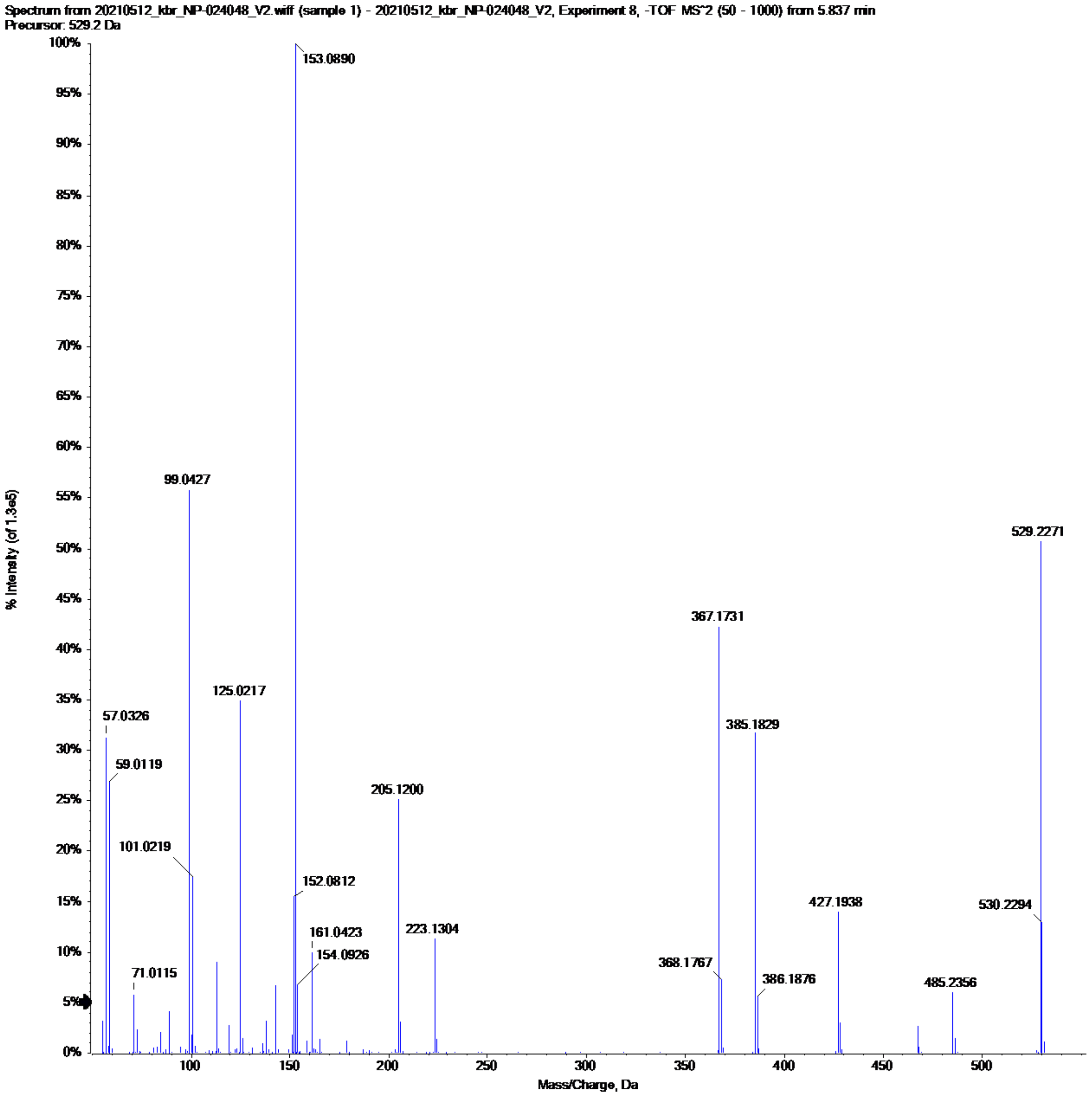

Figure S73: qToF-MS/MS spectrum of standard solution.

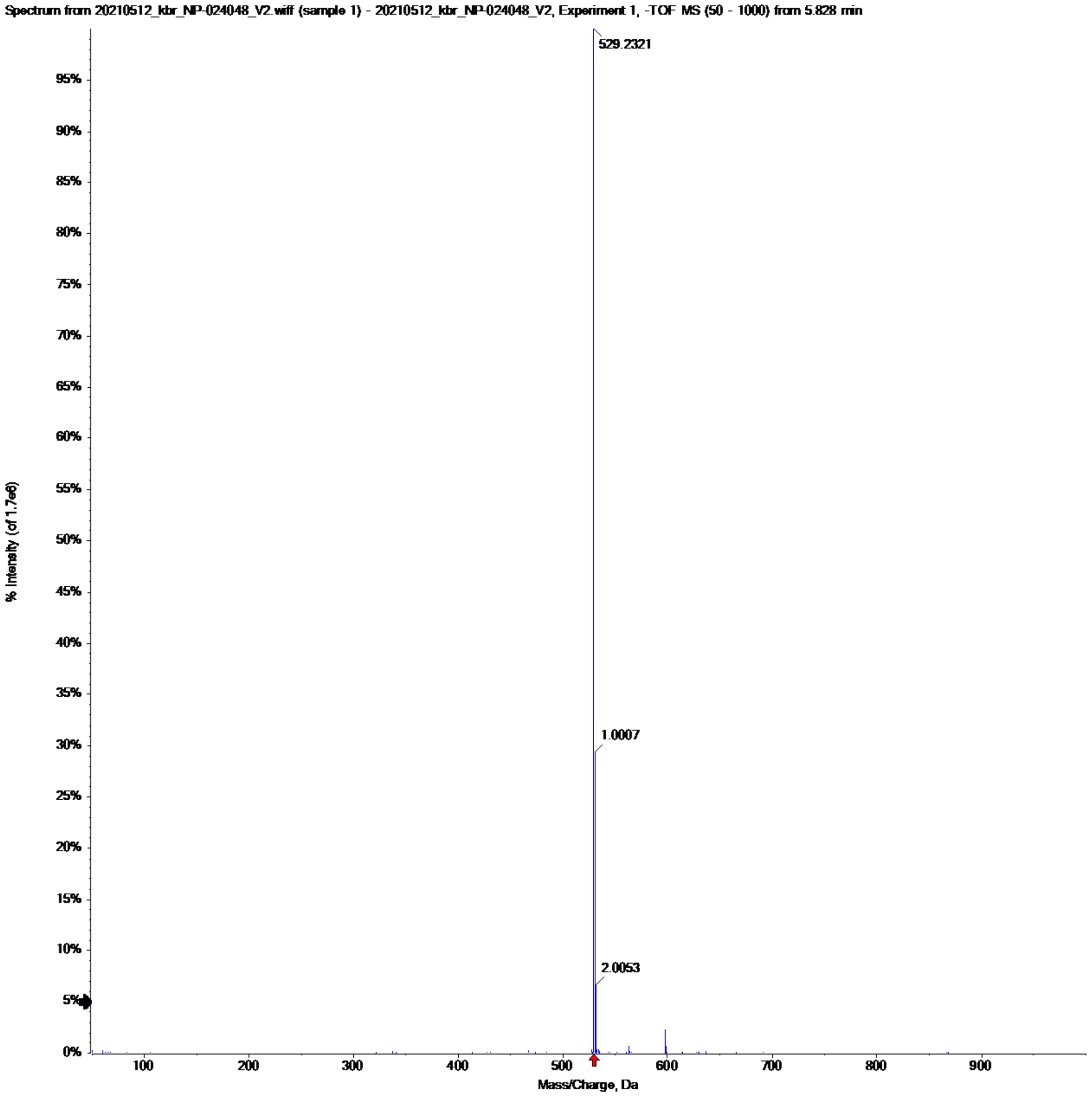

Figure S74: qToF-MS spectrum (survey scan) of standard solution.

NP023236: HMG gluc O

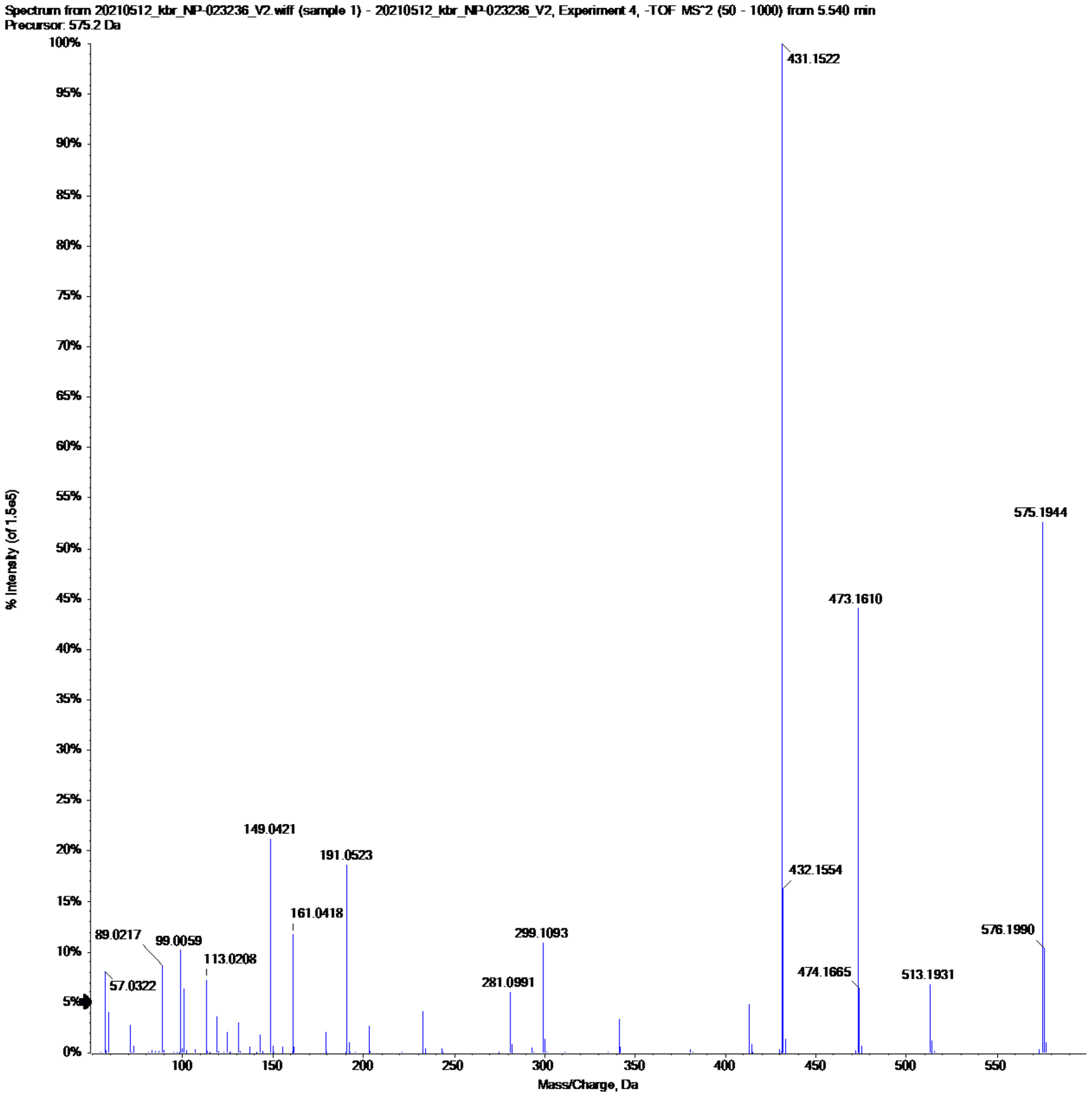

Figure S75: qToF-MS/MS spectrum of standard solution.

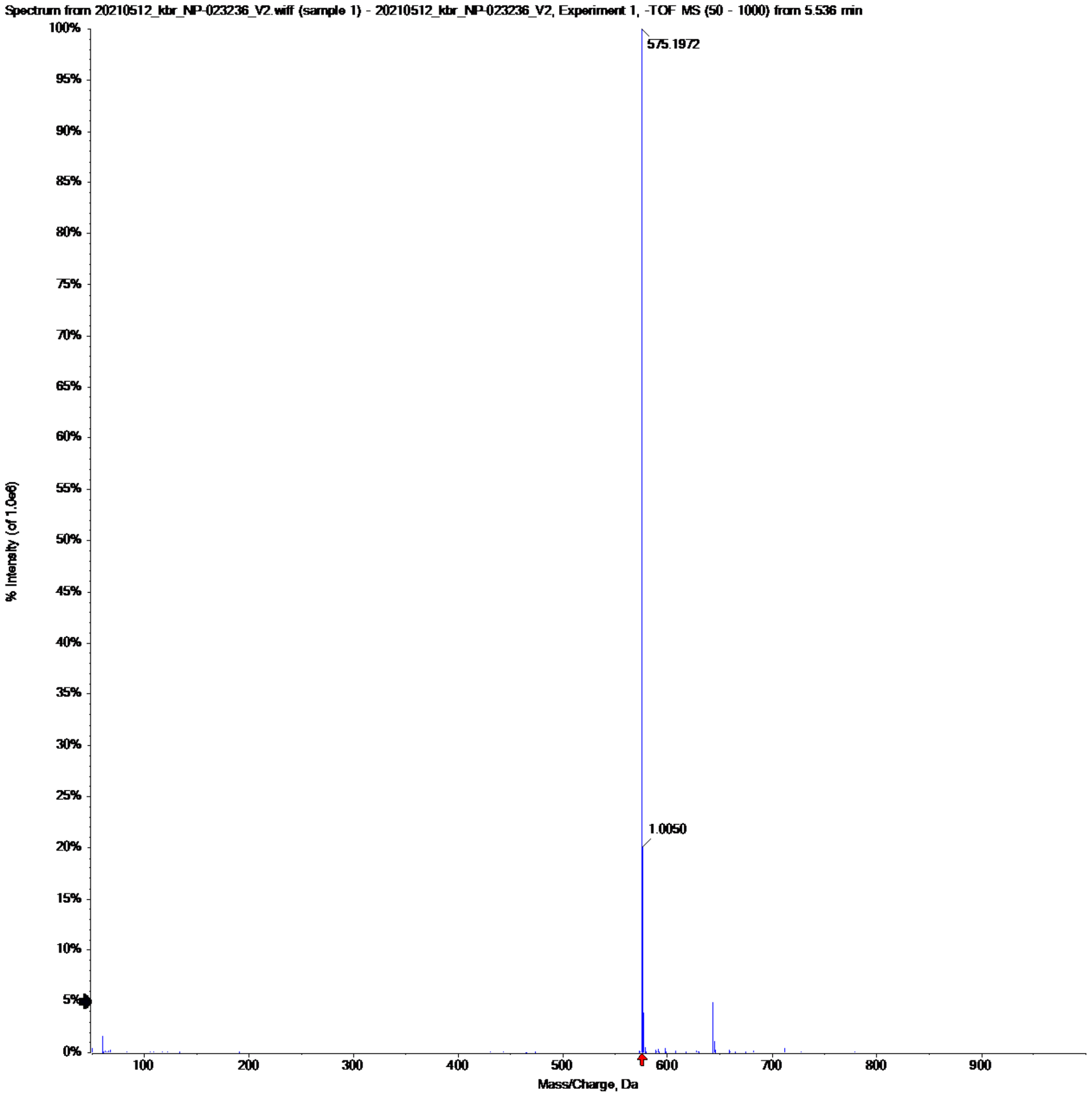

Figure S76: qToF-MS spectrum (survey scan) of standard solution.

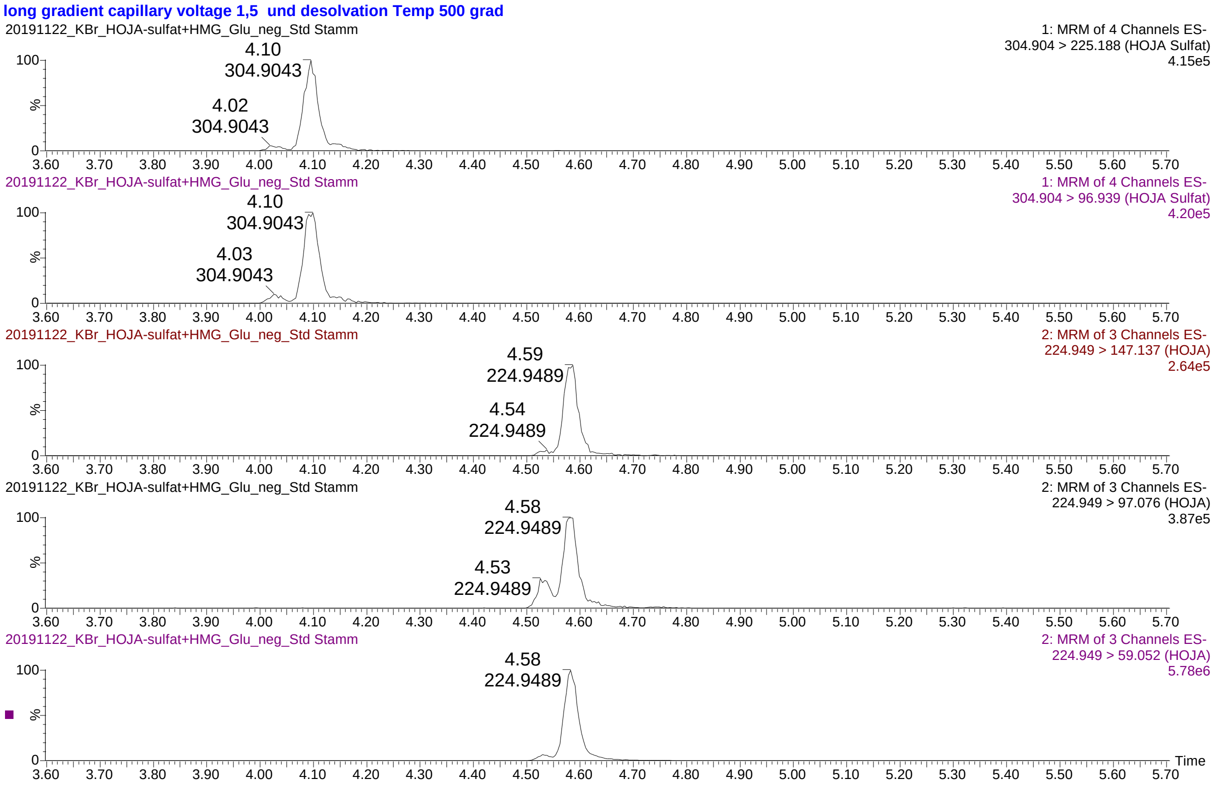

Figure S77: MRM traces of UPLC-MS/MS of HOJA sulfate (1st and 2nd from top) and of HOJA (3rd, 4th and 5th from top) of standard solution.

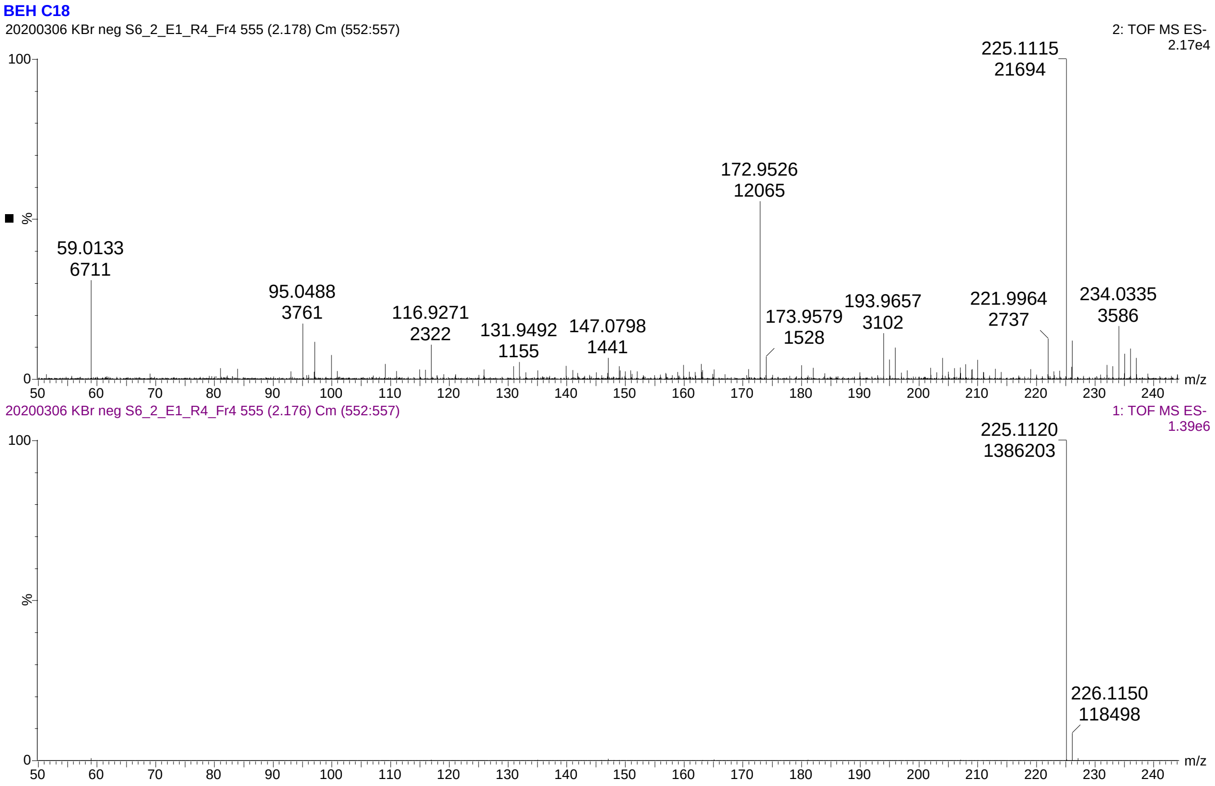

Figure S78: ToF-MS^E spectra of HOJA standard solution with high CE (top) and low CE (bottom).

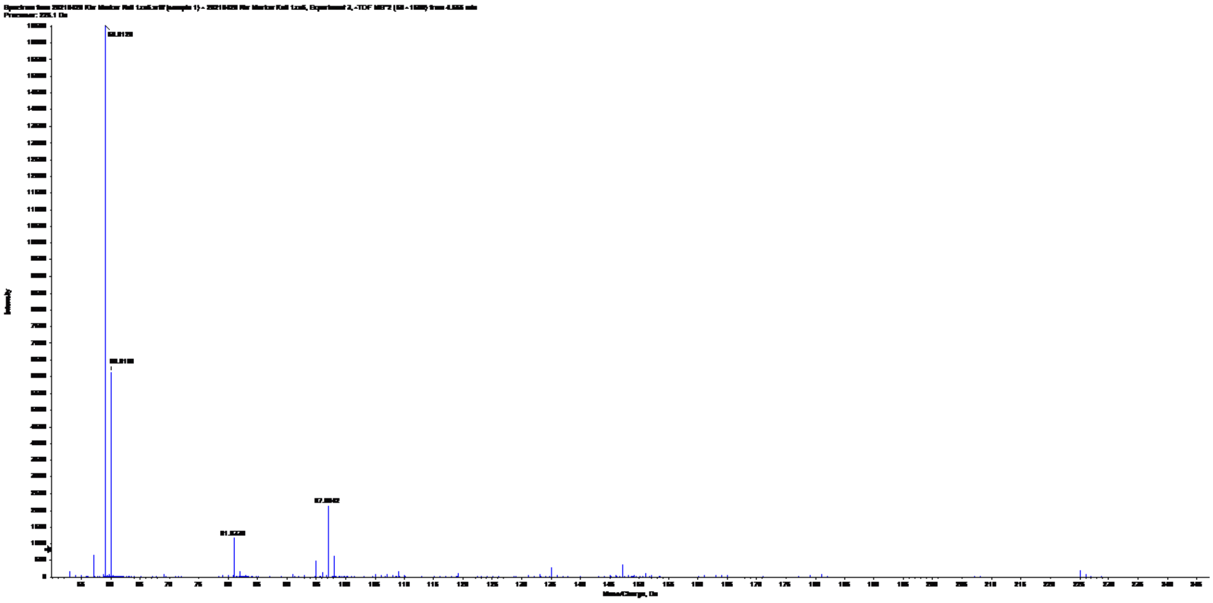

Figure S79: qToF-MS/MS spectrum of standard solution.

(+)-catechin

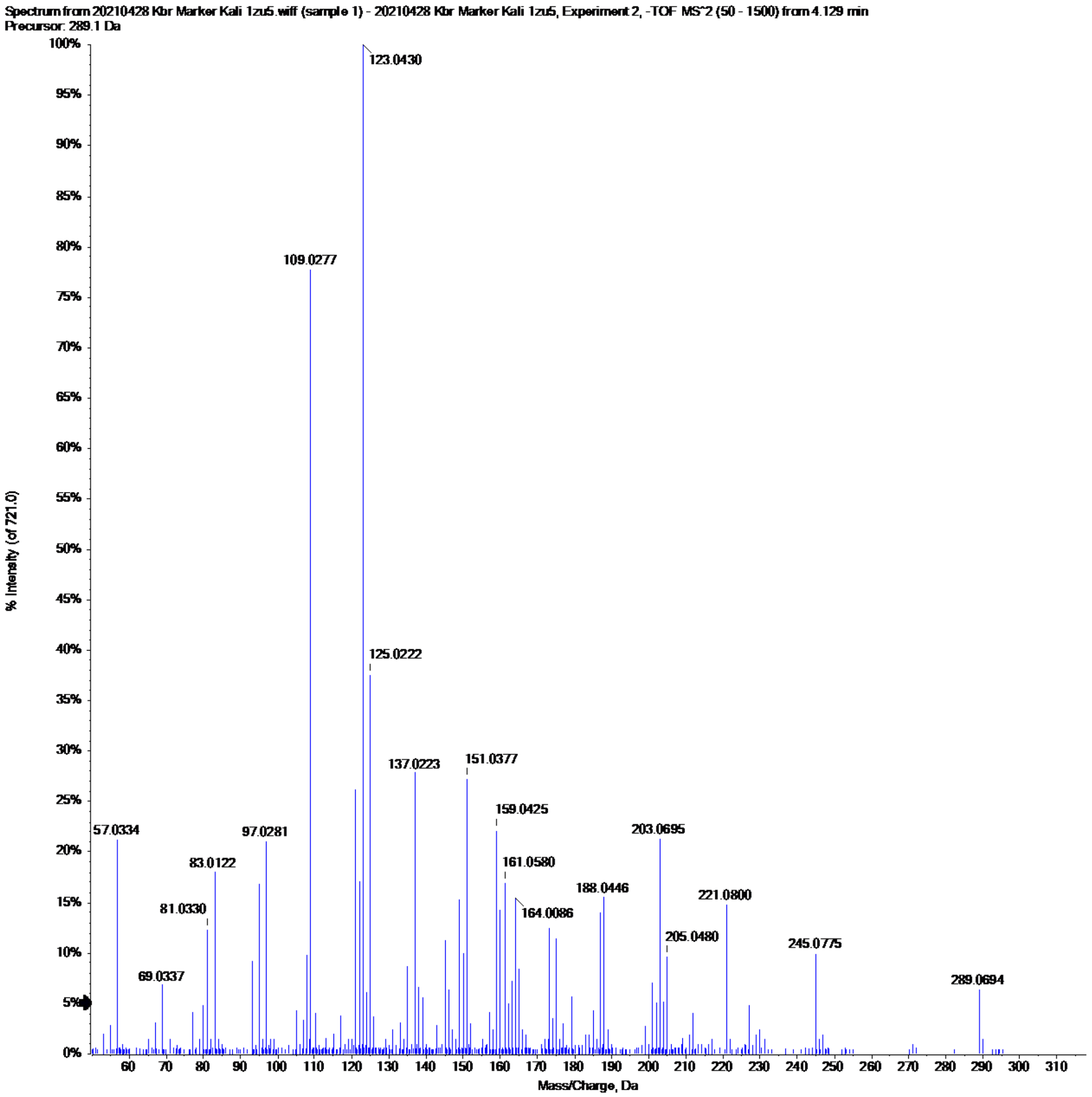

Figure S80: qToF-MS/MS spectrum of standard solution.

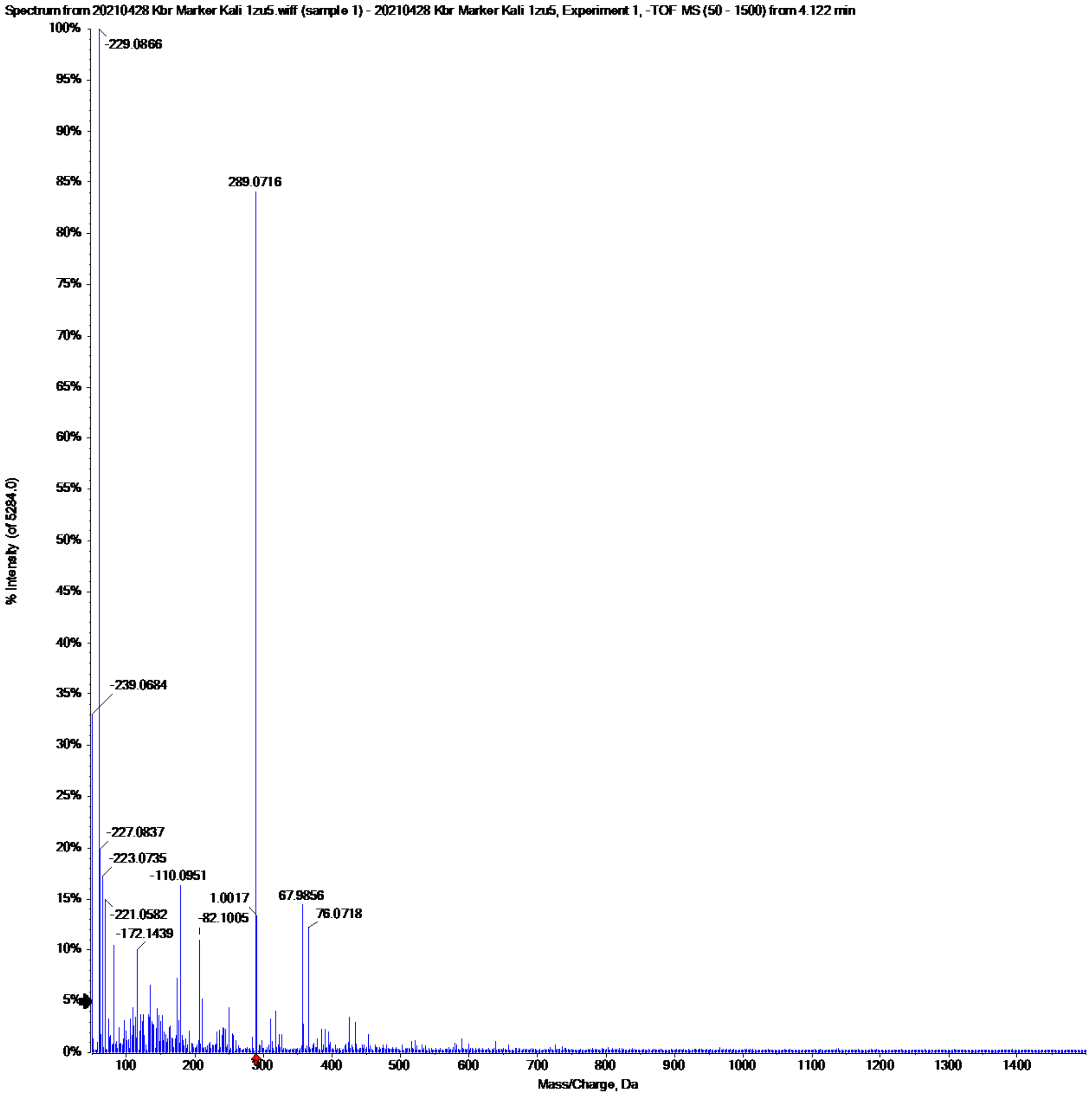

Figure S81: qToF-MS spectrum (survey scan) of standard solution.

(-)-epicatechin

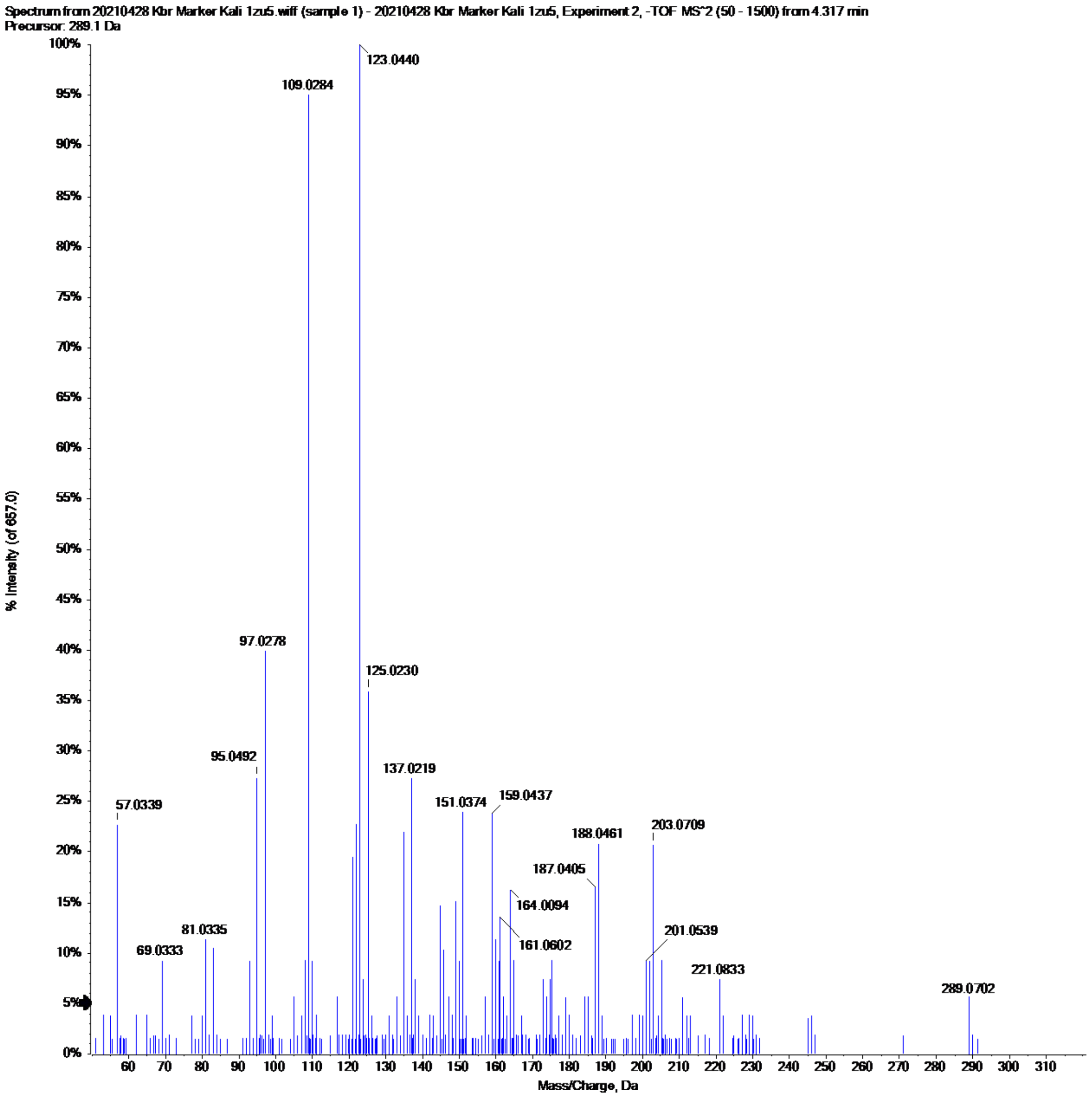

Figure S82: qToF-MS/MS spectrum of standard solution.

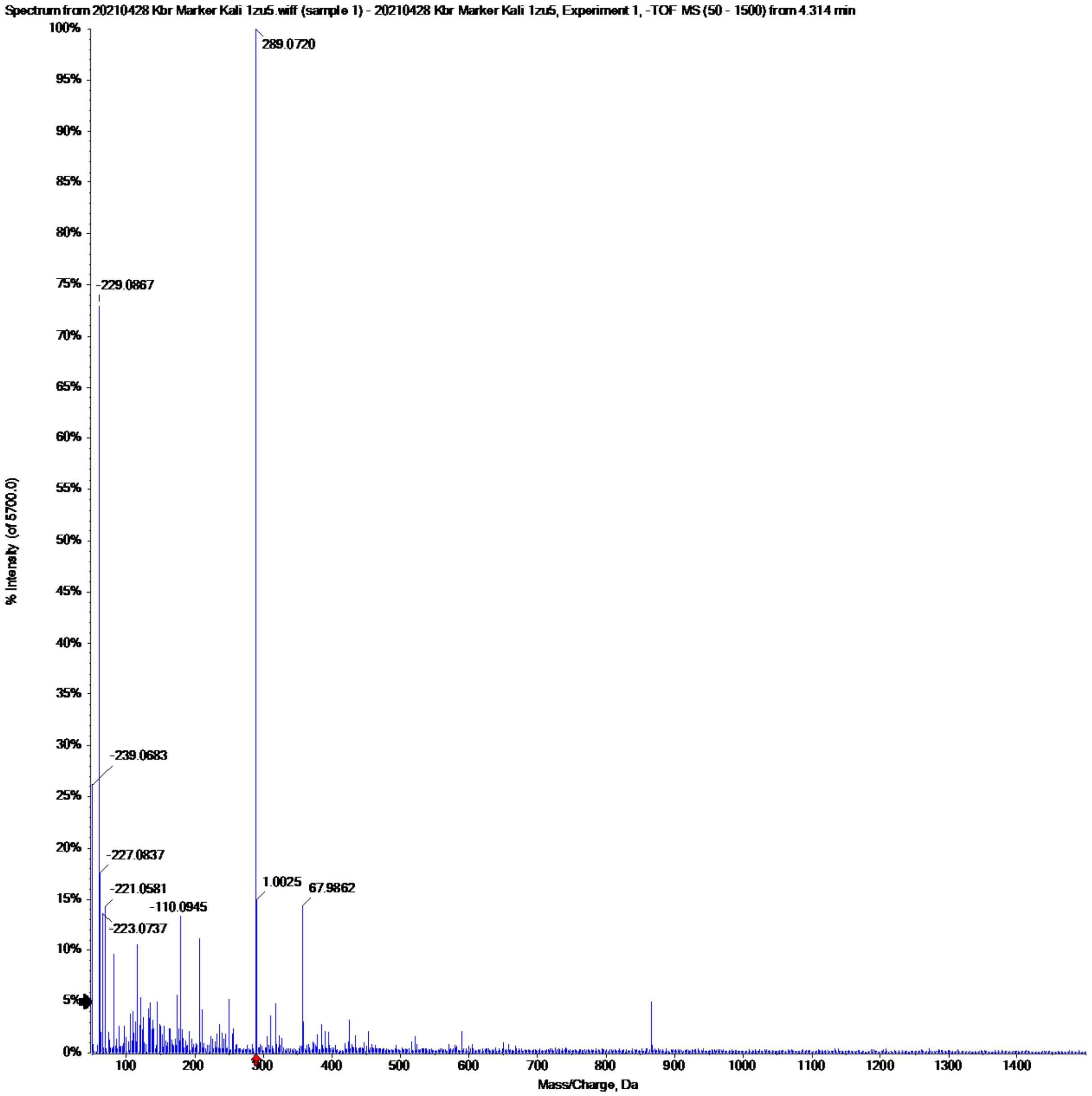

Figure S83: qToF-MS spectrum (survey scan) of standard solution.

9,12,13-(10*E*)-THOA

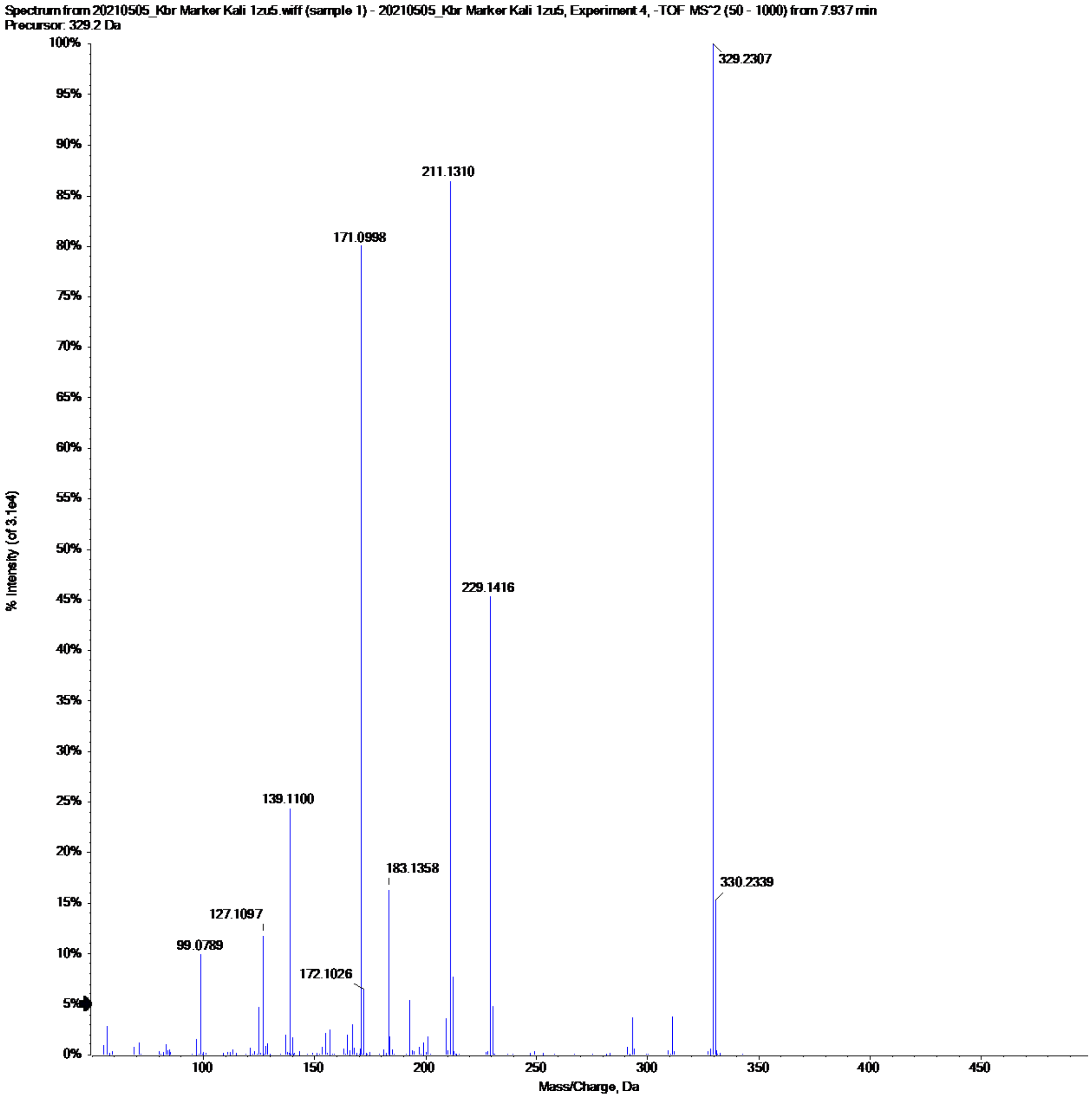

Figure S 84: qToF-MS spectrum (survey scan) of standard solution.

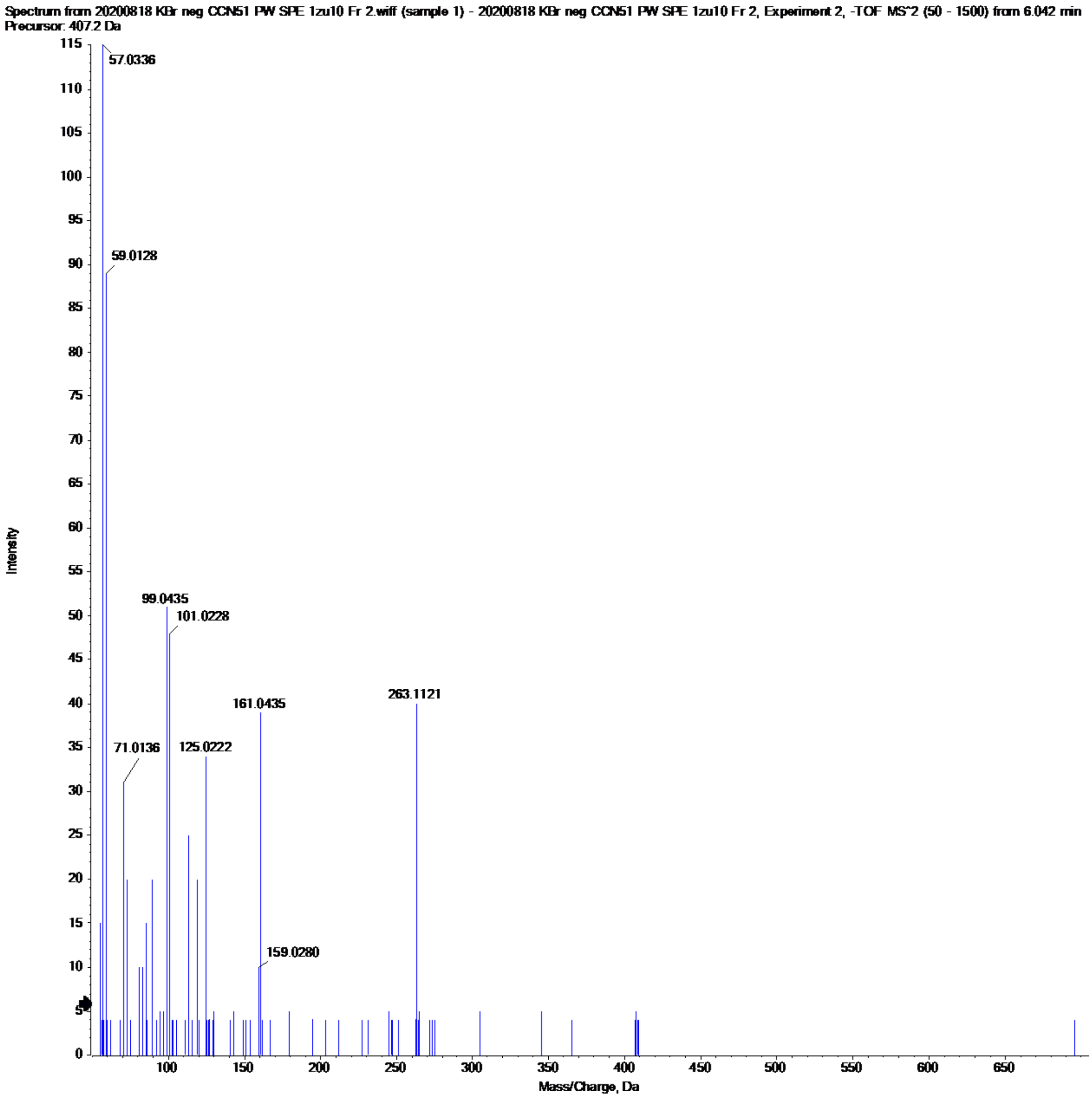

Figure S 85: qToF-MS/MS spectrum of proposed marker No. 4 (6.04 min) with precursors m/z 407.1545, measured in SPE/ HPLC enriched acetone/water-extract of raw cocoa beans.

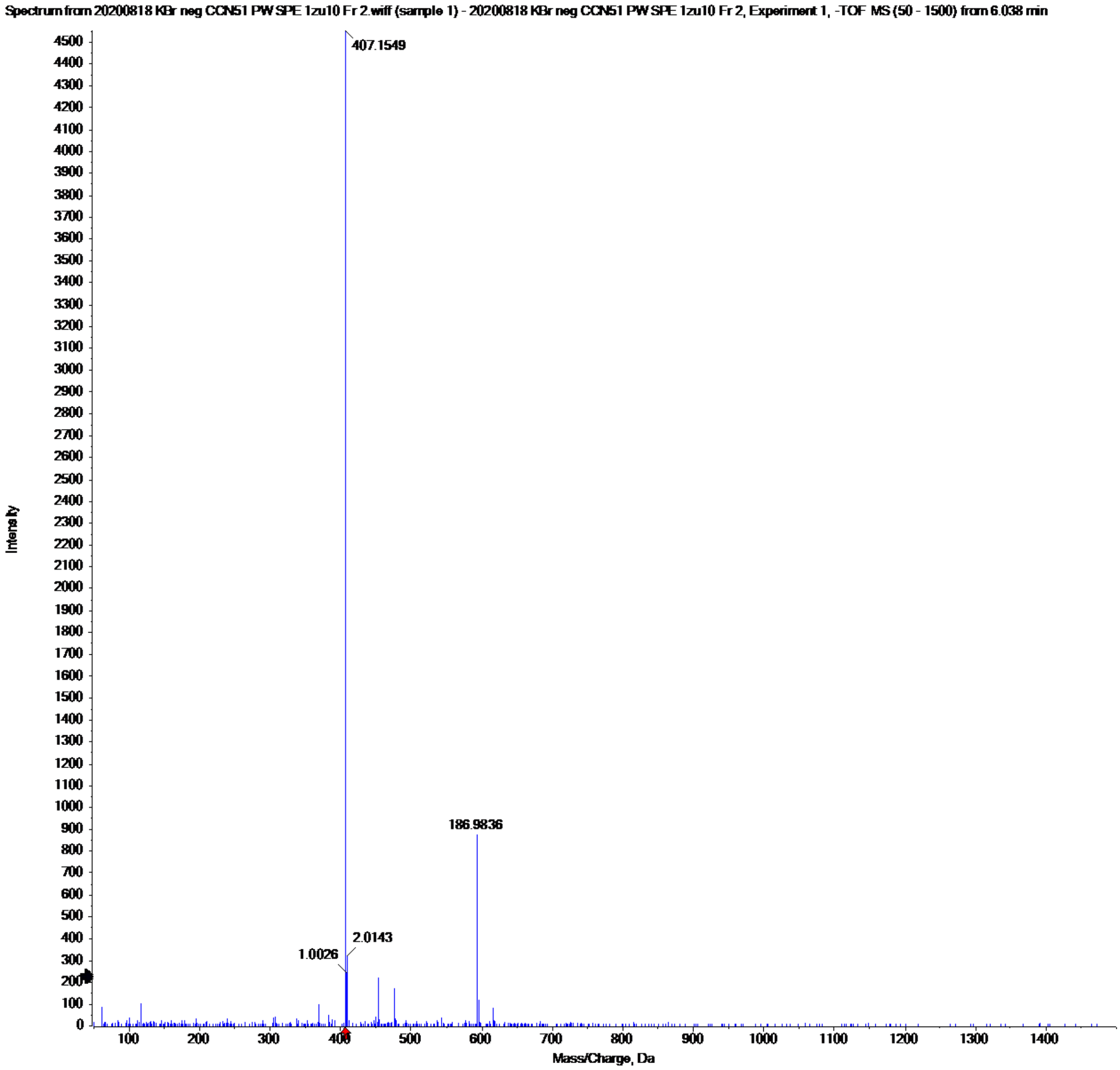

Figure S 86: qToF-MS spectrum (survey scan) of proposed marker No. 4 (6.04 min) with precursors m/z 407.1545, measured in SPE/ HPLC enriched acetone/water-extract of raw cocoa beans.

Marker candidate No. 1, Isomer 1

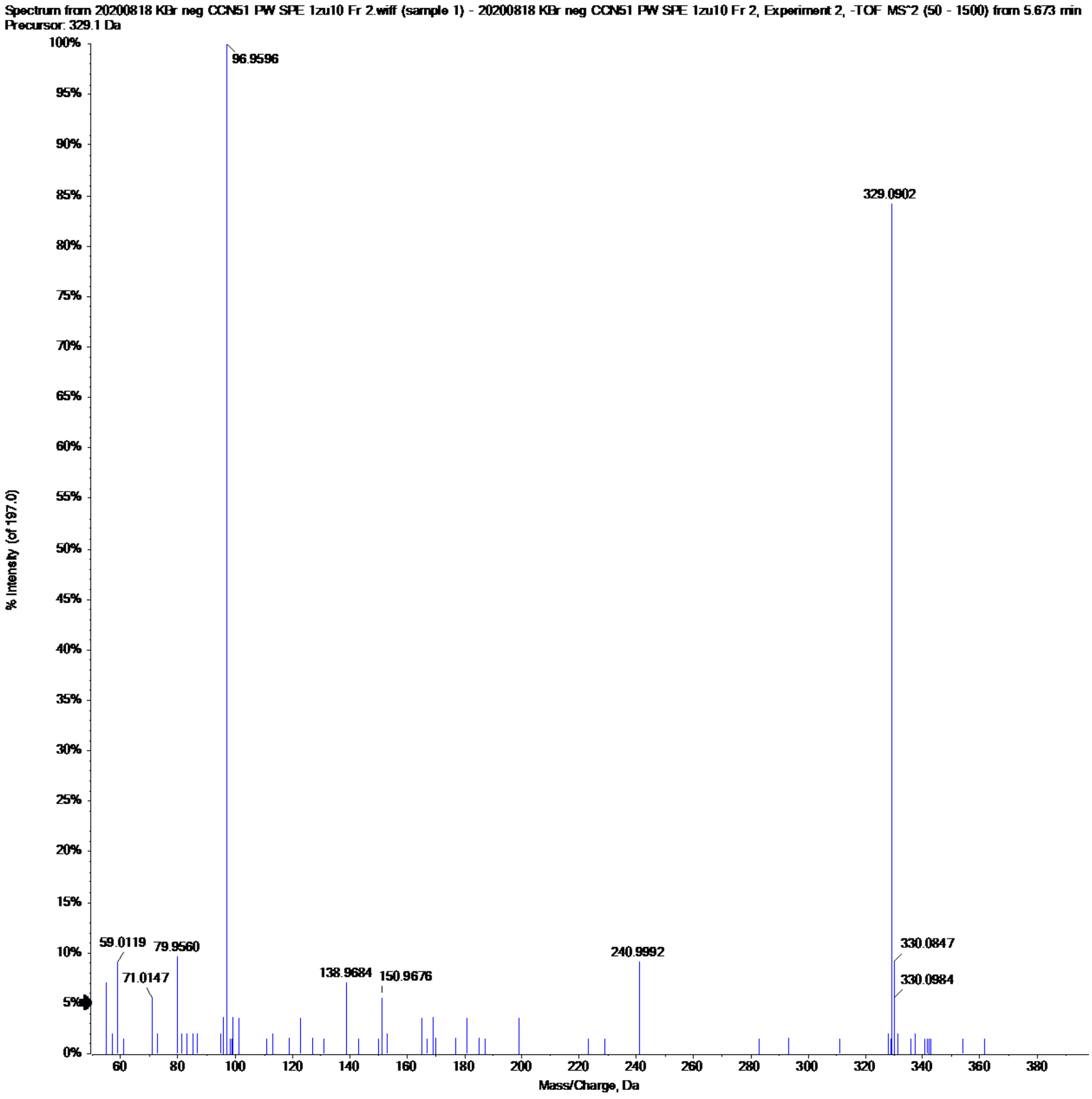

Figure S 87: qToF-MS/MS spectrum of proposed marker No. 1 (5.67 min) with precursors m/z 329.0915, measured in SPE/ HPLC enriched acetone/water-extract of raw cocoa beans.

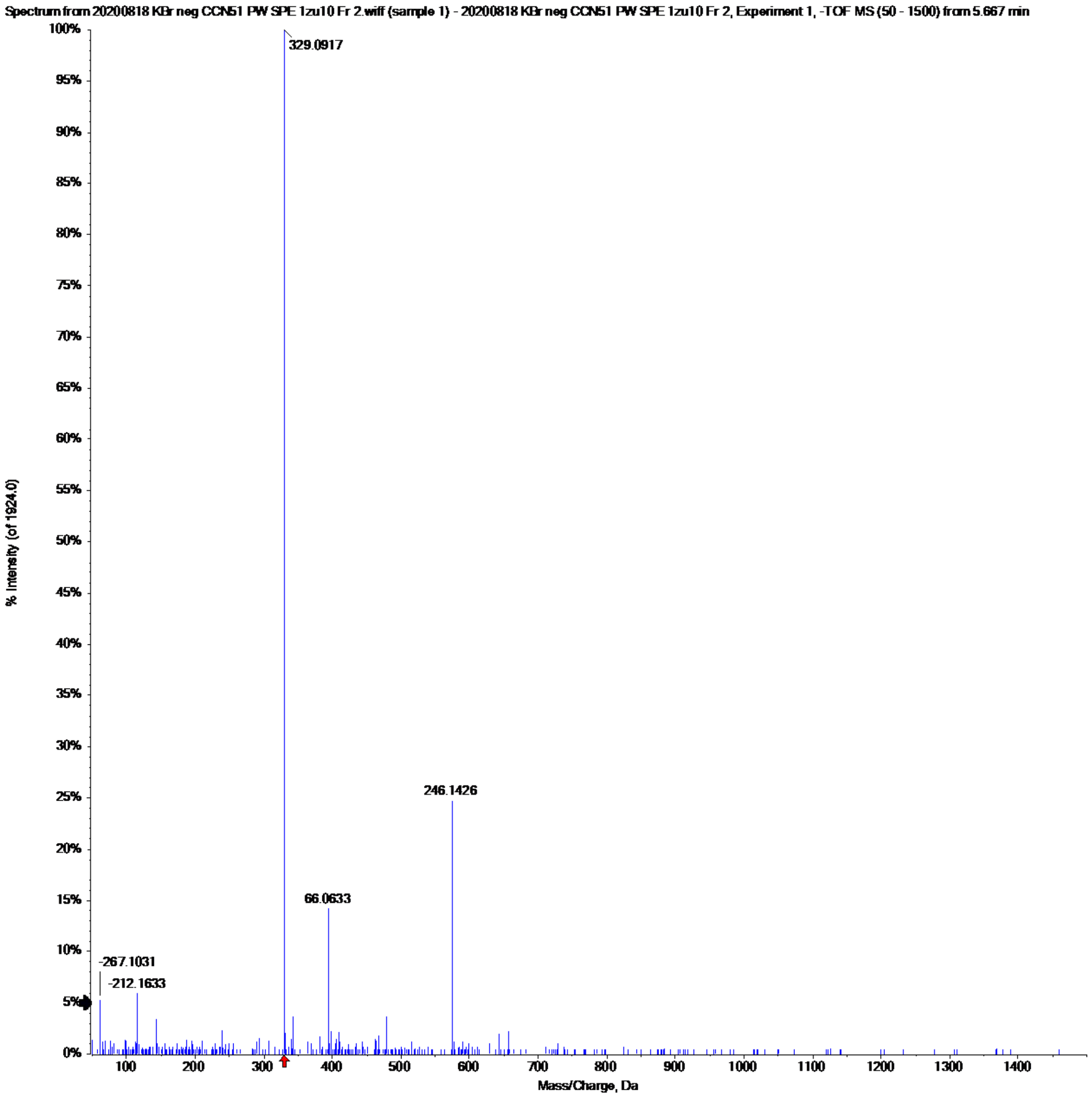

Figure S 88: qToF-MS spectrum (survey scan) of proposed marker No. 1 (5.87 min) with precursors m/z 329.0915, measured in SPE/ HPLC enriched acetone/water-extract of raw cocoa beans.

Marker candidate No. 1, Isomer 2

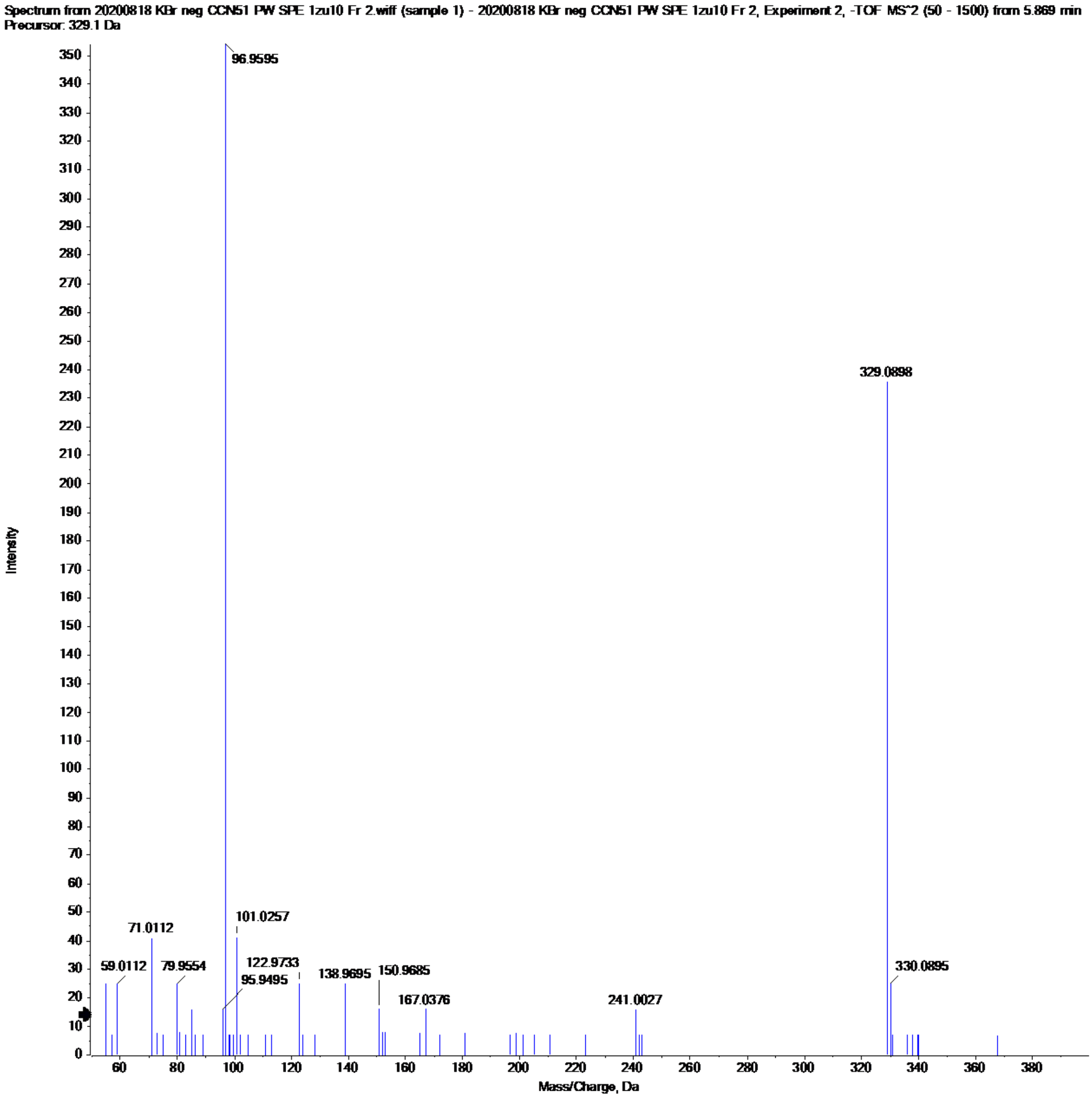

Figure S 89: qToF-MS/MS spectrum of proposed marker No. 1 (5.87 min) with precursors m/z 329.0915, measured in SPE/ HPLC enriched acetone/water-extract of raw cocoa beans.

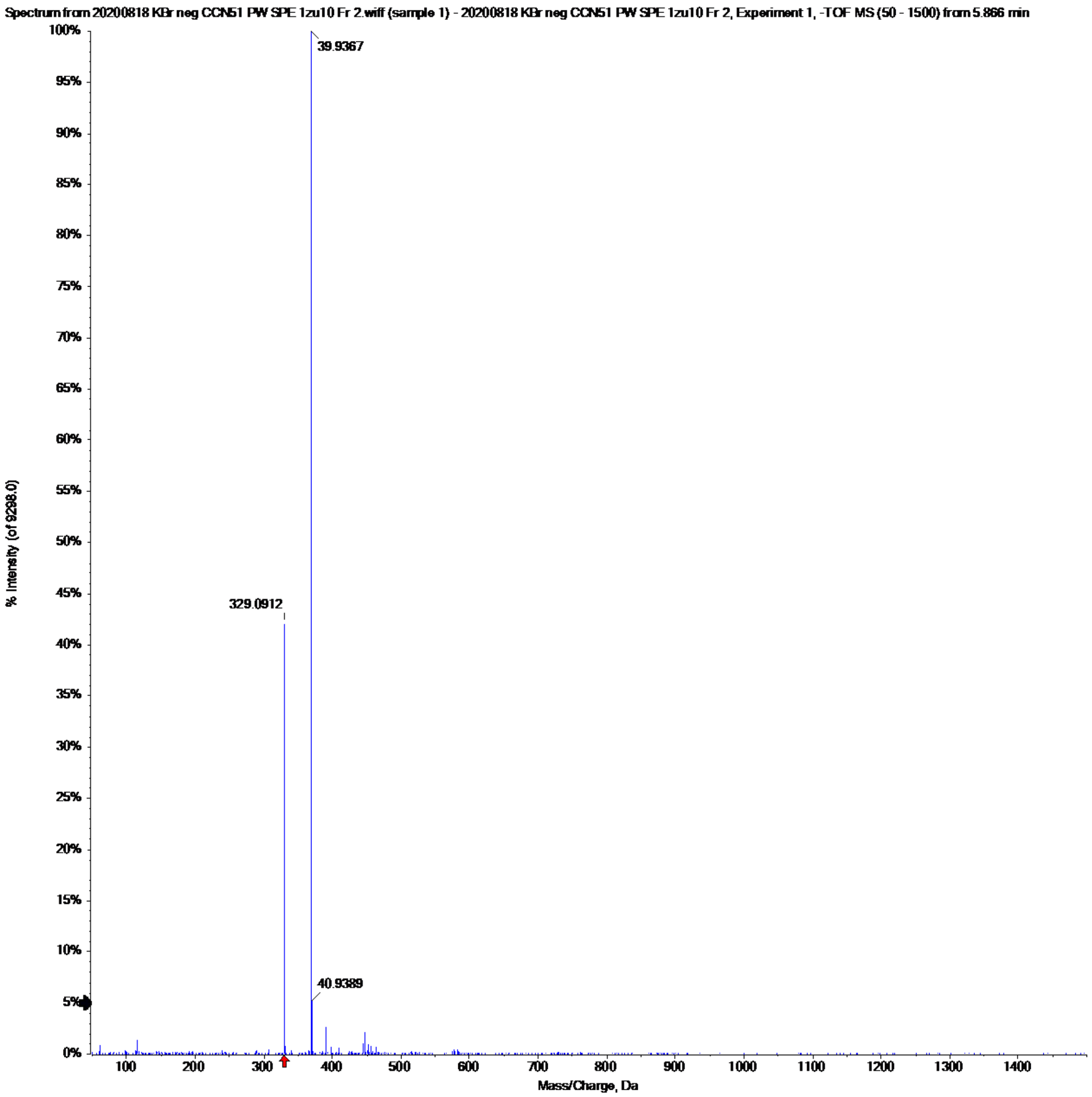

Figure S 90: qToF-MS spectrum (survey scan) of proposed marker No. 1 (5.87 min) with precursors m/z 329.0915, measured in SPE/ HPLC enriched acetone/water-extract of raw cocoa beans.
